# Molecular and genetic diversity in the metastatic process of melanoma

**DOI:** 10.1002/path.4318

**Published:** 2014-01-27

**Authors:** Katja Harbst, Martin Lauss, Helena Cirenajwis, Christof Winter, Jillian Howlin, Therese Törngren, Anders Kvist, Björn Nodin, Eleonor Olsson, Jari Häkkinen, Karin Jirström, Johan Staaf, Lotta Lundgren, Håkan Olsson, Christian Ingvar, Sofia K Gruvberger-Saal, Lao H Saal, Göran Jönsson

**Affiliations:** 1 Department of Oncology, Clinical Sciences, Lund UniversitySweden; 2 CREATE Health Strategic Centre for Clinical Cancer Research, Lund UniversitySweden; 3 Department of Pathology, Lund University and Skåne University HospitalLund, Sweden; 4 Department of Oncology, Skåne University HospitalLund, Sweden; 5 Department of Surgery, Lund University and Skåne University HospitalLund, Sweden

**Keywords:** melanoma, gene expression, deep sequencing, *BRAF*, *NRAS*

## Abstract

Diversity between metastatic melanoma tumours in individual patients is known; however, the molecular and genetic differences remain unclear. To examine the molecular and genetic differences between metastatic tumours, we performed gene-expression profiling of 63 melanoma tumours obtained from 28 patients (two or three tumours/patient), followed by analysis of their mutational landscape, using targeted deep sequencing of 1697 cancer genes and DNA copy number analysis. Gene-expression signatures revealed discordant phenotypes between tumour lesions within a patient in 50% of the cases. In 18 of 22 patients (where matched normal tissue was available), we found that the multiple lesions within a patient were genetically divergent, with one or more melanoma tumours harbouring 'private' somatic mutations. In one case, the distant subcutaneous metastasis of one patient occurring 3 months after an earlier regional lymph node metastasis had acquired 37 new coding sequence mutations, including mutations in *PTEN* and *CDH1*. However, *BRAF* and *NRAS* mutations, when present in the first metastasis, were always preserved in subsequent metastases. The patterns of nucleotide substitutions found in this study indicate an influence of UV radiation but possibly also DNA alkylating agents. Our results clearly demonstrate that metastatic melanoma is a molecularly highly heterogeneous disease that continues to progress throughout its clinical course. The private aberrations observed on a background of shared aberrations within a patient provide evidence of continued evolution of individual tumours following divergence from a common parental clone, and might have implications for personalized medicine strategies in melanoma treatment. Published by John Wiley & Sons, Ltd. www.pathsoc.org.uk

## Introduction

Metastatic melanoma is an aggressive disease, notorious for its resistance to conventional therapy. Although development of targeted therapy in melanomas carrying *BRAF* mutations has revolutionized treatment, a significant number of patients with *BRAF* V600E metastatic melanoma experience recurrence within a few months upon treatment with the BRAF inhibitor vemurafenib 1 or the MEK inhibitor trametinib 2. Treatment resistance could be explained by tumour heterogeneity, i.e. the existence of, or selection for, molecularly distinct subclones with metastatic capability. Supporting this hypothesis, intratumour heterogeneity has been reported in a vemurafenib-resistant subcutaneous melanoma metastasis, which contained a subclone with a *de novo NRAS* mutation 3. Colombino *et al* 4 have reported *BRAF* mutant metastases presumably seeded by a *BRAF* wild-type primary tumour and, more intriguingly, *BRAF* wild-type metastases in the presence of a *BRAF* mutant primary. Together, these findings indicate that some tumours may exhibit profound heterogeneity that contributes to the aggressive clinical course and eventual treatment resistance of melanoma. Indeed, large-scale sequencing studies of different solid cancers, including melanoma, have revealed extensive genetic heterogeneity between individual tumours [5–7]. In contrast to the prevailing theory of metastatic spread originating from the primary tumour at an advanced stage of the disease, recent evidence suggests the parallel development of the primary tumour and metastasis 8, or parallel development of multiple metastases in the same patient 9,10.

We recently identified four molecular subtypes of melanoma tumours using gene expression profiling characterized by differential expression of immune response genes, microphthalmia-associated transcription factor (*MITF*)-regulated genes and proliferation-related genes. These subtypes were named ‘pigmentation’ (*MITF*-high), ‘proliferative’ (*MITF*-low), ‘high-immune’ and ‘normal-like’; 11,12. In the present study, we applied this subtype classification to multiple melanoma tumours from 28 patients previously diagnosed with a single primary melanoma (multiple metastases in 27 patients, and a primary tumour and metastasis in one patient). We identified 14 patients whose multiple tumours had divergent gene expression phenotypes. We further investigated the mutational landscape of the melanoma tumours from 22 of the 28 patients, using targeted gene sequencing and analysis of DNA copy number variation. Melanoma metastases from the same patient had a core of mutations and DNA copy number changes (herein termed ‘shared’ events). However, in all but four patients, mutations specific to one of the tumours within a patient were identified (‘private’ mutations). Our findings highlight the molecular and genetic diversity of metastatic melanoma tumours and indicate the need to redesign personalized medicine strategies for metastatic melanoma.

## Material and methods

### Patients

Tissue from melanoma tumours (*n =* 266) and blood (*n =* 22) were obtained from the Department of Oncology at Lund University. The samples were collected from melanoma patients referred to the Department of Surgery, Skåne University Hospital, for surgical removal of a melanoma tumour. From this cohort, 27 patients with multiple metastases (≥ 2) and one patient with a primary tumour and a subsequent metastasis were identified. With the exception of melanomas from one patient, all tumours were intermittently sun-induced melanomas or melanomas with an unknown primary (*n =* 5). Clinical characteristics of the 28 patients are shown in Table1. The study was approved by the ethics committee of the Lund University (Diary No. 101/2013).

**Table 1:** Molecular subtype and clinical characteristics of the 63 metastases

Age at dx	Primary tumour					Metastasis 1			Metastasis 2			Metastasis 3		
	Breslow (mm)	Clark	Type	Site	Date of dx	Type	Subtype	Date of dx	Type	Subtype	Date of dx	Type	Subtype	Date of dx
	*Patient 1*													
28	1.52	3	SSM$	Trunk	198907	Regional lymph node	Pigm	200103	Regional lymph node^#^	Prol	200106	Distant subcutaneous*	Prol	200109
	*Patient 2*													
46	4	4	NMM$	Trunk	199609	Regional lymph node	Pigm	199802	In transit lymph node^#^	Pigm	199807			
	*Patient 3*													
67	4	4	NMM$	Trunk	200603	In transit lymph node	Pigm	200608	Regional lymph node	High-imm	200611			
	*Patient 4*													
42	4	4	NMM$	Trunk	200711	Regional lymph node	Pigm	200802	Regional lymph node	Pigm	200904	Regional lymph node	Pigm	201011
	*Patient 5*													
63	2	N/A	N/A	Upper extremity	200006	Regional lymph node	Pigm	200804	Regional lymph node	Pigm	200903			
	*Patient 6*													
46	1.7	3	SSM	Trunk	200603	Regional lymph node	Pigm	200804	Distant lymph node	High-imm	200902			
	*Patient 7*													
60	1.7	3	NMM$	Lower extremity	200702	In transit lymph node	Pigm	200903	In transit lymph node	Pigm	201012			
	*Patient 8*													
NA	Unknown primary					In transit lymph node	Pigm	200810	In transit lymph node	Pigm	201005	In transit lymph node	Norm	201110
	*Patient 9*													
NA	Unknown primary					Lymph node	Pigm	200904	Distant	Norm	201012			
	*Patient 10*													
74	3.3	4	NMM€	Trunk	200903	Regional lymph node	High-imm	200907	Regional lymph node	High-imm	201003	Regional lymph node*#	Norm	201105
	*Patient 11*													
NA	Unknown primary					Subcutaneous met	Pigm	200907	Lymph node	Pigm	200910			
	*Patient 12*													
NA	Unknown primary					Lymph node	Prol	201007	Lymph node	Prol	201011	Subcutaneous*	Prol	201110
	*Patient 13*													
42	3.4	3	NMM€	Trunk	200710	In transit subcutaneous	High-imm	201011	In transit subcutaneous	High-imm	201102			
	*Patient 14*													
77	3.5	3	SSM	Trunk	199601	Regional lymph node	High-imm	199609	Local recurrence	Pigm	199611	Local recurrence	High-imm	199702
	*Patient 15*													
80	12	3	NMM	Upper extremity	199409	Primary tumour	Prol	199409	Regional lymph node	Prol	199412			
	*Patient 16*													
61	2	3	N/A	Trunk	198908	Regional lymph node	Pigm	199411	Distant met	Pigm	199604			
	*Patient 17*													
64	1.65	3	SSM	Lower extremity	198507	Distant subcutaneous	High-imm	199705	Distant met	Prol	199904			
	*Patient 18*													
76	2.35	4	N/A	Lower extremity	199212	In transit subcutaneous	High-imm	199710	In transit subcutaneous	Pigm	199803			
	*Patient 19*													
31	1.4	4	SSM	Lower extremity	199405	Local recurrence	Pigm	199711	Regional lymph node	Prol	199806			
	*Patient 20*													
47	N/A	N/A	Acral	Nail	199711	Regional lymph node	Pigm	199712	Regional lymph node	Pigm	200407			
	*Patient 21*													
31	3.05	4	NMM	Upper extremity	199608	Distant subcutaneous	High-imm	199903	Distant subcutaneous	High-imm	200010			
	*Patient 22*													
63	2.4	4	SSM	Trunk	199801	Regional lymph node	Prol	200008	Regional lymph node	Prol	200104			
	*Patient 23*													
61	7	4	NMM	Trunk	200011	Regional lymph node	High-imm	200108	Regional lymph node	Pigm	200109			
	*Patient 24*													
NA	Unknown primary					Distant met	High-imm	200503	*>Lymph node	Pigm	201111			
	*Patient 25*													
50	6	4	NMM$	Lower extremity	200001	Regional lymph node	High-imm	200504	Regional lymph node	Prol	200605			
	*Patient 26*													
73	11	4	NMM€	Lower extremity	200504	Regional lymph node	Prol	200505	Regional lymph node	Prol	200606			
	*Patient 27*													
75	3.4	4	SSM	Upper extremity	199605	Regional lymph node	Pigm	200511	Regional lymph node	High-imm	200612			
	*Patient 28*													
192911	1.7	4	SSM	Lower extremity	200312	Regional lymph node	Prol	200606	Regional lymph node	Prol	200611			

$ Primary ulcerated.

€ Primary not ulcerated.

# Interferon treatment prior to detection of investigated lesion.

* ^*^Chemotherapy treatment prior to detection of investigated lesion.

dx, diagnosis.

### Gene expression profiling

RNA from tumours (*n =* 266) was subjected to microarray analysis. Tumours were classified into the four gene expression subtypes (pigmentation, proliferative, high-immune and normal-like), as previously described 11.

### Next-generation sequencing

For targeted-capture deep sequencing (see supplementary material, Supplementary materials and methods, and Table S1, for sequencing data metrics), 1697 cancer genes were selected based on literature-documented association to cancer and the Catalogue of Somatic Mutations in Cancer (COSMIC) database (http://www.sanger.ac.uk/genetics/CGP/cosmic/; see supplementary material, Table S2). For details on targeted-capture deep sequencing, low-coverage whole-genome sequencing and DNA copy number analysis, see Supplementary materials and methods.

## Results

### Gene expression profiling reveals molecular diversity in multiple melanoma metastases following a single primary tumour

**Figure 1:**
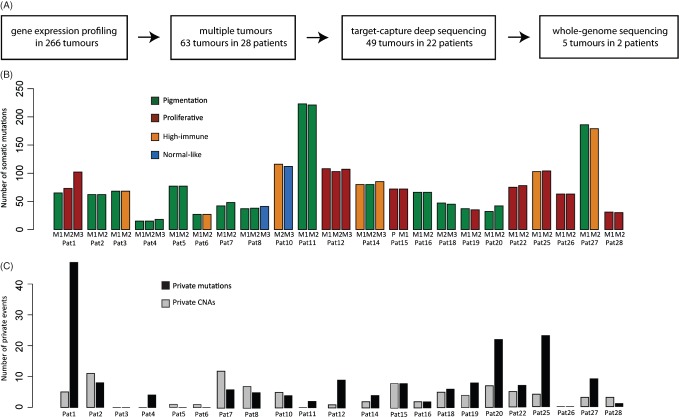
Study design depicting the molecular and genetic analysis of melanoma metastatic tumours. (A) Flow chart of the main methods used in the study. (B) Bar plot showing numbers of somatic mutations in multiple metastases from 22 patients. Each melanoma tumour was classified into the four gene expression phenotypes: pigmentation, green; proliferative, red; high-immune, orange; or normal-like, blue, and coloured accordingly. (C) Bar plot showing numbers of private copy number aberrations (CNAs) and mutations per patient. Private mutations within each patient were defined as being present in only one of the analysed tumours in the patient

We attempted to characterize the molecular footprint of metastatic progression in malignant melanoma by a sequence of analyses on metastatic melanomas from patients with a single primary tumour (Figure 1A). Using microarray analysis, we analysed the gene-expression profiling of 266 melanoma tumours and identified 28 patients from whom at least two melanoma lesions diagnosed at different time points had been sampled (62 metastases in total; Table1). We applied our previously described gene-expression signature 11,12 to the 62 melanoma lesions identified. In 14 of 28 patients (50%), melanoma metastatic tumours from the same patient belonged to different molecular subtypes (Table1). Moreover, in four of these 14 patients, the later-occurring metastases had a proliferative phenotype, while the earlier tumours exhibited a pigmentation/high-immune/normal-like phenotype (patients 1, 17, 19 and 25). Notably, these proliferative-classified late melanoma metastases had a more advanced disease stage than the corresponding earlier tumour. For patient 25, both lesions were regional lymph node metastases; however, the first lesion was removed when the patient was diagnosed with regional metastatic disease, while the second lesion was removed when the patient was diagnosed with a generalized disease.

We never observed a change of molecular phenotype from the proliferative class in an earlier tumour to a different phenotypic class in a later metastasis, which indicates that the proliferative phenotype is associated with a late stage of the disease. For example, a local recurrence and a regional metastasis were collected for patient 19. The local recurrence was classified into the pigmentation phenotype, whereas the corresponding lymph node metastasis, diagnosed 4 years later, was classified into the proliferative phenotype. Patient 17 presented with a relatively thin primary melanoma (Breslow 1.65 mm) and 12 years later developed an in-transit metastasis that was classified as high-immune phenotype. A distant intestinal metastasis, diagnosed 2 years later, was classified into the proliferative phenotype.

To confirm the observation of a tumour phenotype switch, we also performed unsupervised hierarchical clustering, using the 75% most variable genes across the entire dataset. Overall, 12 of 14 with no change in gene expression phenotype clustered as closest neighbours in the hierarchical dendrogram, and the results were significantly associated with the subtype (centroid) classification shown in Table1 (*p =* 0.049, Fisher's exact test; see supplementary material, Figure S1).

### Somatic mutations and copy number changes accompanying melanoma metastatic progression

Target enrichment and deep sequencing of all exons in 1697 genes (see supplementary material, Table S2) was performed on DNA samples from 49 tumours and matched blood from 22 patients included in the gene-expression analysis. Mean on-target sequence coverage of the tumour and normal DNA was 198x–594x (see supplementary material, Table S1), with 93–97% of targeted bases at 10x or higher coverage. Using VarScan somatic software 13, we detected 978 somatically acquired synonymous and non-synonymous single-nucleotide variants (SNVs), 21 indels in the coding sequence and 11 splice site mutations. We used CONTRA 14 to identify exon-specific copy number aberrations (CNAs; see supplementary material, Figure S2, Table S3).

**Figure 2:**
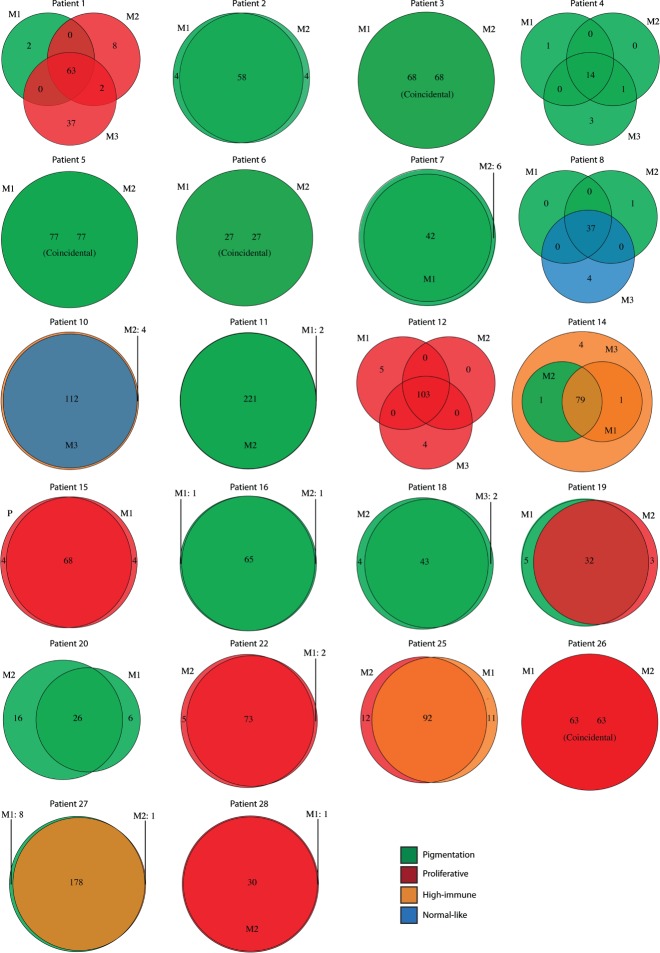
The mutational landscape of metastatic melanoma tumours: number of mutations (including shared and private somatic mutations) in the tumours of 22 patients depicted as Venn diagrams. The circle colours represent gene expression phenotypes: pigmentation, green; proliferative, red; high-immune, orange; or normal-like, blue. M1, metastatic lesion 1; M2, metastatic lesion 2; M3, metastatic lesion 3

First, we investigated the number of somatically acquired mutations in relation to progression of the disease in the 22 patients (Figures 1B, C, 2). Private mutations affected genes such as *CTNNB1*, *FBXW7*, *GRIN2A*, *TSC2*, *RUNX1* and *PTCH1* (for a list of identified mutations, see supplementary material, Tables S4–S25). *BRAF* and *NRAS* mutations, when present in the first metastasis, were always preserved in subsequent metastases. Patients with a tumour phenotype change to the proliferative class (*n =* 3) had a higher frequency of private mutations and CNAs (median 27, range 12–52) compared to patients without this phenotype change (*n =* 19) (median 9, range 0–29) (*p =* 0.03, Mann–Whitney test).

We investigated DNA CNAs in the tumours and found common losses on chromosome 9p and 10q corroborating previous studies on melanoma 15. The single acral melanoma harboured *CDK4* amplification. Next, we searched for CNAs in genes known to be affected by CNAs in melanoma (*BRAF*, *KIT*, *MITF*, *CCND1*, *CDK4*, *PTEN*, *CDKN2A* and *AKT3*). None of the tumours showed focal amplifications of *AKT3*, *MDM2*, *KIT* or *MITF*. Two patients showed *CDK4* focal amplification and one patient had *CCND1* focal amplification; however, these were preserved in all three patients during progression. Frequent deletions and copy number gains were found in *CDKN2A*, *PTEN* and *BRAF*, with the majority of changes being preserved during progression. Differences were found in patient 1, where only the first metastasis had a heterozygous deletion of *CDKN2A*. A change in gene expression phenotype was observed in this patient, where the first metastasis was classified in the pigmentation phenotype and the two latter metastases were classified in the proliferative class. Furthermore, in patient 27 the second metastasis had acquired additional *BRAF* gene copies. A change in gene expression phenotype occurred in this patient as well, where first metastasis was classified as pigmentation and second metastasis as high-immune response (see supplementary material, Figure S3).

### Detailed analysis of somatic CNAs and chromosomal rearrangements in patients 1 and 2

**Figure 3:**
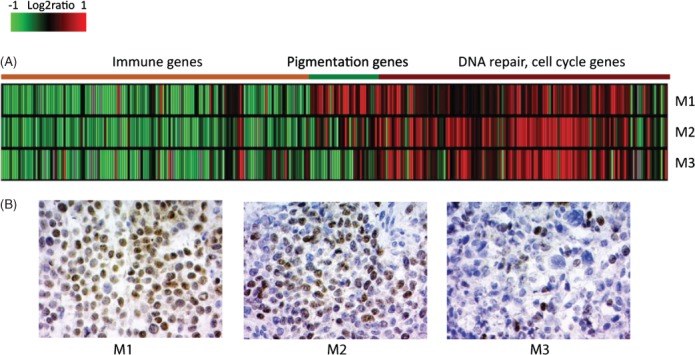
Molecular heterogeneity in patient 1. (A) Expression heat map of the subtype-specific genes in the melanoma metastases: red, over-expressed genes; green, down-regulated genes. (B) Microphthalmia-associated transcription factor (MITF) immunohistochemistry of metastatic tumour sections from patient 1. Cell nuclei are shown in blue. M1, metastatic lesion 1; M2, metastatic lesion 2; M3, metastatic lesion 3

**Figure 4:**
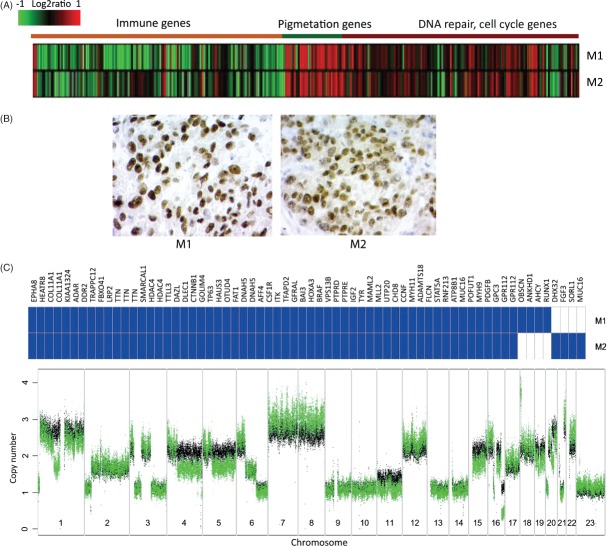
Molecular and genetic heterogeneity in patient 2. (A) Expression heat map of subtype-specific genes in the melanoma metastases. (B) Microphthalmia-associated transcription factor (MITF) immunohistochemistry in both metastases. (C) Mutated genes (blue) identified in metastases (upper panel). Genome-wide copy number profiles of M1 (black) and M2 (green) are superimposed onto each other and illustrate differences between the two metastases (lower panel)

Two patients (1 and 2) were selected for a more detailed investigation of the molecular footprint of metastatic progression. Patient 1 was selected on the basis of discordant subtype classification and a high number of private mutations (Figures 1B, 2). The three metastases displayed gradual loss of expression of pigmentation genes from M1 to M3, as shown by gene-expression analysis and MITF immunohistochemical (IHC) staining (Figure 3A, B). The gradual loss of MITF protein expression was reflected by a decrease in the number of MITF-positive melanoma cells in the tumour, with the least number of MITF-positive cells observed in M3. In patient 2, we found a low number of private mutations and both available metastases were classified into the pigmentation subtype, corroborated by MITF IHC (Figure 4A, B).

To enrich our analysis for genomic rearrangements and CNAs, we performed low-coverage whole-genome sequencing of the five metastases in these two patients and matched lymphocyte DNA. Mean haploid genome sequence coverage was 1.9x–4.2x in these tumours (physical coverage 7.4x–13x). The metastases from each patient showed similar DNA copy number profiles (see supplementary material, Figure S4A), which indicates shared clonality of the tumours. We also observed a number of private aberrations. In patient 1, M2 and M3 shared many CNAs absent in M1, and vice versa, consistent with our previous results for these tumours 10. The genomes of M2 and M3 diverged from that of M1 in 23 regions of differential copy number. Only one CNA, an 850 kbp gain at chromosome 3q13.31, was exclusive to M2, while none was specific to M3 (see supplementary material, Table S3, Figure S4A).

We also derived interchromosomal rearrangements, using BreakDancer 16. To narrow down the candidate list, the positions of breakpoints were integrated with CNAs to isolate copy number change-supported interchromosomal rearrangements. This approach considerably decreased the number of candidate rearrangements suggested by BreakDancer, at the price of eliminating possible copy-number neutral rearrangements. We identified and validated by Sanger sequencing two interchromosomal rearrangements in patient 1 (see supplementary material, Figure S4). A genomic rearrangement involving chromosomes 7 and 15 was found in all three metastases, while a rearrangement between chromosomes 3 and 4 was exclusive to M1 (see supplementary material, Figure S4B). Moreover, the tumour genomes of all metastases from patient 1 were estimated as diploid by genome alteration print (GAP) analysis 17. Together, these findings indicate common clonal origin of the metastases, while providing evidence for ongoing evolution of the cells forming the individual tumours. We identified and validated two interchromosomal rearrangements, both observed in M1 and M2 from patient 2: one between chromosomes 1 and 20, and another between chromosomes 2 and 20 (see supplementary material, Figure S4A). Although M1 and M2 from patient 2 were both estimated as triploid tumours by GAP analysis, they displayed only a few private mutations and did not change molecular phenotype; we identified many DNA CNA differences on chromosomes 2, 6, 7, 8, 12, 16, 17, 18, 20, 21 and 22, which indicated a continued evolution of the tumour genome (Figure 4C).

### Somatic mutation patterns in metastatic progression in patients 1 and 2

**Figure 5:**
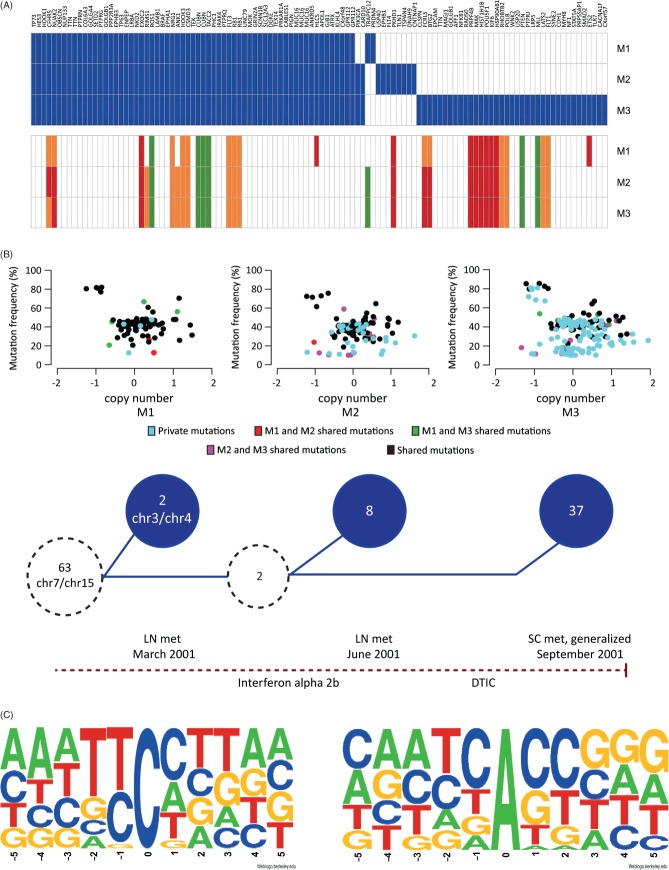
Genetic heterogeneity in patient 1. (A) Mutated genes (upper panel, blue) and corresponding copy number (lower panel) in the three metastases from patient 1. Copy number: white = 2, green = 1, orange = 3, red = 4 inferred from GAP results. (B) Progression model: (upper panel) Somatic mutations (exonic, splice-site and intronic) plotted by log_2_ copy number (*x* axis) versus variant allele frequency (*y* axis); colours are indicated in the legend; (lower panel) the common precursor clone (dotted circle on the far left) contained 63 exonic or splice site mutations and a rearrangement involving chromosomes 7 and 15, all found in all three metastases. M1 harboured two private mutations and another genomic rearrangement, between chromosomes 3 and 4. Hypothetical common predecessor of M2 and M3 (smaller dotted circle) developed from the precursor clone and contained two additional mutations. Finally, M2 acquired eight additional private exonic mutations, and M3 37 additional private mutations. Red dotted line represents time, with diagnoses and treatments indicated; LN, lymph node; SC, subcutaneous; met, metastasis; DTIC, dacarbazine. (C) Sequence context of shared (all three metastases, left) and M3 private (right) mutations demonstrates clear pattern differences

The tumours from patient 1 shared 63 somatic exonic or splice site mutations (Figure 5A; see also supplementary material, Supplementary materials and methods). Among the shared alterations, notably, *RB1* harboured two mutations: a tandem GG > AA base substitution converting the tryptophan 516 into an immediate stop in exon 17, and a splice site mutation. Both mutations occurred in a gained chromosomal region with copy number 3 in all three metastases. The variant allele frequencies in M1, M2 and M3 (nonsense, 30% in each; splice site, 55%, 55% and 65%, respectively) is consistent with inactivation of all three alleles, in a classic tumour suppressor inactivation manner.

In addition to this core of mutations, M1 carried two private exonic mutations, a synonymous substitution in *PRDM4* and *TRAPPC12*. M2 and M3 shared two mutations, including an oncogenic substitution in *PIK3CA*. In addition, M2 had eight private exonic mutations and M3 harboured 37 (Figure 5A; see also supplementary material, Supplementary materials and methods). Based on these findings, we present a progression model of patient 1, which is depicted in Figure 5B. The shared mutations appear at heterozygous allele frequency at diploid copy number (average 39–43%; see supplementary material, Figure S5) in the major clone of the tumours. However, the private mutations in M2 and M3 both appear in this group and at lower allele frequencies at diploid copy number, suggesting that additional subclone(s) evolved during progression (Figure 5B).

Of the 37 mutations private to M3, *PTEN* harboured a splice site mutation at 80% allele frequency that, together with chromosome 10 loss, likely contributed to the lower transcript level of *PTEN* observed in this tumour (see supplementary material, Table S26). While the presence of whole chromosome 10 hemizygous deletion in all three metastases implicates it as an early event in development of the precursor clone, the high allele frequency for the M3 private *PTEN* mutation points to it being a defining hit in the establishment of M3. Other notable mutations exclusively found in M3 were a frame-shift deletion in the DNA double-strand break (DSB) repair gene *RAD50* and a *CDH1* splice site mutation.

For patient 2, both metastases belonged to the pigmentation phenotype. They shared 58 mutations, including *BRAF* V600E, and contained few private mutations (M1, 4; M2, 4) (Figure 4C; see also supplementary material, Supplementary materials and methods). For example, a missense mutation in *RUNX1*, which encodes a transcription factor frequently mutated in leukaemia, was found only in M1.

The within-patient shared mutations in all 22 patients were predominantly of the C:G → T:A type (range 64–94%, median 89%). This transition has been repeatedly reported to be the predominant type of base substitution in diverse types of cancer 18. The sites of the shared C:G → T:A mutations were usually preceded by another pyrimidine at the 5′ position (Figure 5C), pointing towards UV-induced DNA damage as the mechanism of mutation 19, in line with previous studies in melanoma 6,7. In patient 1, 94% (63/67) of the shared exon or splicing SNVs were of this type. Notably, the base substitution pattern of SNVs private to M3 deviated from this pattern: A:T → G:C transitions constituted 80% (24/30) of the M3 private SNVs (significant enrichment, as compared to shared mutations; Fisher's test, *p <* 0.0001; and to M1/M2 private mutations, *p =* 0.014). We investigated the close-range sequence context of these sites, by comparing base composition within a 10-position window around the mutation site to that of 500 randomly picked coding A/T sites. The base composition of the +1 and +2 positions was significantly different from that of the random sites (Fisher's test, Benjamini–Hochberg adjusted *p =* 0.007 and *p =* 0.009, respectively). This non-random pattern suggests an as-yet unknown mutation mechanism that needs further investigation. The private mutations found in the other patients did not display the A:T → G:C pattern, but instead displayed the classical UV-induced pattern C:G → T:A.

## Discussion

Using molecular and genetic analysis of multiple metastatic melanoma lesions from 28 patients, we provide evidence of a heterogeneous gene-expression pattern between different metastatic lesions and, in 82% of patients, occurrence of private mutations. We found that known driver mutations, such as oncogenic *BRAF* and *NRAS* mutations, were always somatically preserved, whereas others (such as *PTEN* and *CTNNB1*) were somatically acquired during progression. Our findings highlight molecular and genetic diversity in the metastatic process that might hinder personalized genomic strategies in melanoma, based on single tumour biopsies.

From a comprehensive set of 266 melanoma tumours subjected to gene-expression profiling, we identified 28 patients with at least two melanoma lesions diagnosed at different time points. Classification of tumours into our previously described phenotypes, based on gene-expression profiles 11,12, showed that 50% of these patients had metastases belonging to different phenotypes. We observed that tumours of the proliferative phenotype were of a more advanced stage than the corresponding earlier tumour, indicating that the described molecular phenotypes might reflect a progression model of melanoma. However, this notion needs to be verified in larger cohorts. We did not identify any patients with metastases belonging to different phenotypes that had the first metastatic tumour classified into the proliferative phenotype. Interestingly, we found decreased expression of melanocyte differentiation markers in proliferative classified tumours, which indicate that a dedifferentiation process might be involved in the switch from MITF-high phenotype to a MITF-low (proliferative) phenotype (Figure 3B). Combined analysis of gene-expression data with results from targeted deep sequencing from patient 1 suggest a continued tumour evolution, exemplified by accumulation of additional mutations and by molecular phenotype switching in later metastases. Our findings further support the idea of phenotype switching previously described in experimental melanoma model systems 20,21. However, based on our findings, we cannot conclude that molecular phenotype switching is associated with an increased amount of somatically acquired mutations during tumour progression.

Further supporting our data, protein expression studies have shown extensive heterogeneity in a primary melanoma 22 and intra-tumour heterogeneity has also been shown in renal cell carcinoma using whole-exome sequencing 8. On the extreme end of tumour evolution is the late-occurring metastasis three (M3) in patient 1, with 37 private coding sequence mutations. This high mutation rate was not reflected at the level of general genomic instability, as the copy number profile of M3 is almost identical to that of M2 from patient 1. This lack of correlation between the numbers of mutations and CNAs within a tumour has been reported previously 23. However, we only investigated a part of the genome (5.5 Mb) for mutations and, although improbable, it is possible that some of the tumours preferentially acquired mutations in other genes. Furthermore, the mutation frequency of the newly acquired mutations in M2 and M3 from patient 1 was found at a wide range (10–80%), indicating the evolution of both major and minor clones. Indeed, we found a *PTEN* mutation at allele frequency of 80%, indicating its presence in the major clone in M3 of patient 1.

The presence of the strong UVR mutagenesis signature in all tumours was consistent with previous studies in melanoma 6,7,23. By contrast, the majority (24/30) of the patient 1, M3 private base substitutions were of the A:T → G:C type. Although the aetiology of this substitution to our knowledge remains unknown, the over-representation and the non-random distribution of the bases at the +1 and +2 positions speaks against a stochastic process and in favour of the action of an internal or external agent. Whether this effect is mediated by the alkylating agent dacarbazine (DTIC), which was used to treat the patient between the M2 and M3 diagnoses, remains to be investigated. Of note, DTIC has been described to mainly induce C:G → T:A transitions 24,25. This mutational signature might also be attributed to ADAR-like DNA deaminases 26 or MLH1-deficiency 27. Further studies are required to determine the significance and context of this mutational signature.

In summary, we have provided evidence for a complex metastatic process of melanoma. The genetic and molecular diversity observed in metastatic melanoma tumours challenge personalized genomic strategies that are based on single tumour biopsies. In melanoma, research on resistance to novel therapeutic options (such as *BRAF* inhibition) has resulted in identification of several molecular alterations that confer resistance. Whether these mechanisms are acquired or somatically preserved remains to be determined. However, our results highlight the molecular and genetic heterogeneity of melanoma tumours and the capacity of melanoma cells to adapt to external agents. Identification of shared molecular patterns may contribute to more robust biomarkers, as well as treatment-predictive markers.

## Abbreviations

CN, copy number; CNA, copy number aberration; DSB, double-strand break; DTIC, dacarbazine; IHC, immunohistochemical; FDR, false-discovery rate; SNV, single-nucleotide variant; UVR, ultraviolet radiation; UVB, ultraviolet B radiation.

## Supplementary material on the internet

The following supplementary material may be found in the online version of this article:

Supplementary materials and methods

Figure S1. Hierarchical clustering of melanoma samples. (A) Unsupervised clustering using the 75% most variable genes. (B) Clustering using the centroid genes

Figure S2. Copy number profiles of the melanoma metastatic tumours, based on CONTRA analysis of the targeted deep-sequencing data

Figure S3. DNA copy number changes in melanoma-relevant genes across the patients

Figure S4. Various types of somatic alterations identified in the tumours of patients 1 and 2, using whole-genome sequencing

Figure S5. Variant allele frequencies of the shared and M3 private mutations in patient 1

Table S1. Sequencing data metrics

Table S2. Genes (*n =* 1697) in the target enrichment panel

Table S3. Regions of differential copy number between metastases in patient 1

Tables S4–25. Coding sequence (exonic and splice site) mutations in patients 1–28

Table S26. Processed microarray expression data on multiple metastases

Table S27. Raw microarray expression data

## Supporting Information

Supplementary materials and methods

Hierarchical clustering of melanoma samples. (A) Unsupervised clustering using the 75% most variable genes. (B) Clustering using the centroid genes (*n =* 500). Genes were taken from centroids defined in the Harbst *et al* study 1. Hierarchical clustering was performed using Pearson correlation and Ward as a distance measure

Copy number profiles of the melanoma metastatic tumours (different tumours of the same patient are indicated as M1, M2, etc.), based on CONTRA analysis of the targeted deep-sequencing data. Segmentation was performed using gain and loss analysis of DNA (GLAD). Segments defined by GLAD are shown in red

DNA copy number changes in melanoma-relevant genes across the patients. Nine genes were selected, based on previous studies showing frequent alterations in melanoma tumours

Various types of somatic alteration identified in the tumours of patients 1 and 2, using whole-genome sequencing. (A) Circos plots: copy number profile in the inner circle (blue). Interchromosomal rearrangements were validated by Sanger sequencing (purple lines). Mutated genes are coloured by mutation type, indicated in the legend. (B) Validation of the chromosome 3–4 rearrangement in patient 1. The rearrangement was supported by discordant read pair mappings (in green and yellow) and by coincidence of both break points with a copy number break. This rearrangement was confirmed by Sanger sequencing. The absolute copy numbers are based on results from GLAD analysis

Variant allele frequencies of the shared and M3 private mutations in patient 1. Only mutations in diploid regions were considered

Sequencing data metrics

Genes (*n =* 1697) in the target enrichment panel

Regions of differential copy number between metastases in patient 1

Coding sequence (exonic and splice site) mutations in patients 1–28

Processed microarray expression data on multiple metastases

Raw microarray expression data
