# Supplementary material for: Molecular and genetic diversity in the metastatic process of melanoma
Source: J Pathol. 2014 Jan 27;233(1):39–50. doi: 10.1002/path.4318 (PMC4359751; doi:10.1002/path.4318)
Supplement: Supplementary file 1 — Supplementary materials and methods [file path0233-0039-sd1.doc]

**<Supplementary material>**

+A: **Supplementary materials and methods**

+B: Detailed information on patients and samples

Tissue from melanoma metastases (*n =* 266) and blood (*n =* 22) were obtained from the Department of Oncology at Lund University (Table 1). Surgery was performed by a single experienced melanoma surgeon (CI). The most tumour-dense parts of the removed tissue were selected for molecular studies. Moreover, using sequence-derived allele data we checked each tumour sample for distinct separation in allele frequency plots. The study was approved by the local ethics committee of Lund University (Diary No. 191/2007).

Patient 1 was diagnosed with a 1.52 mm-thick melanoma on the back in July 1989. In March 2001, the patient developed a lymph node metastasis in the right fossa subclavicularis (M1) and started treatment with interferon*-α*2b. Despite the treatment, the patient developed another lymph node metastasis in the same area in June 2001 (M2). Interferon was replaced by Dacarbizine (DTIC). However, in September 2001 the disease became generalized; a subcutaneous metastasis was resected at this stage (M3). The patient died in October 2001.

Patient 2 was diagnosed with a 4 mm-thick melanoma on the back in September 1996. In December 1997, the patient developed a lymph node metastasis in the right axilla; this tumour was not available for this study. In February 1998, the patient developed a second metastasis in the axilla (M1) and was treated with interferon*-α*2b. In July 1998, a subcutaneous in-transit metastasis was diagnosed (M2). The patient died in February 1999.

+B: Extraction

Fresh-frozen metastases stored at –80^o^C were homogenized and subjected to DNA and RNA isolation, using an AllPrep kit (Qiagen, Hilden, Germany). Sections (3 × 10 μm) of paraffin blocks containing primary tumours were macro-dissected to exclude as much normal tissue as possible. DNA from primary tumours was extracted using a QIAamp DNA FFPE Tissue kit (Qiagen). DNA from blood was isolated using a DNeasy Blood and Tissue kit (Qiagen).

+B: Gene-expression profiling

Melanoma tumours (*n =* 266) were analysed using the whole-genome gene-expression assay (Illumina, CA, USA) on HT-12 v4 arrays. The tumours were classified into the four molecular subtypes, pigmentation, proliferative, high-immune and normal-like, as previously described . Normalized, log2 and mean-centred merged on gene symbol data of all multiple metastases is found in Table S26 and raw microarray data are found in Table S27.

+B: Targeted-capture deep sequencing

+C: **Design**

Genes (*n =* 1697) were selected based on the COSMIC database and literature-documented association to cancer (Table S1). Agilent SureSelect target enrichment design covered 5 527 405 bases by 120 bp-long tiling probes.

+C: **Library preparation**

2 μg DNA was sheared using Covaris with the following settings: duty cycle, 10; intensity, 5; cycles/burst, 200; time, 240 s. Adapters were ligated to the end-repaired, adenylated and phosphorylated fragments. Each sample was labelled with a unique six nucleotide-long barcode that, together with the seventh base (ligation-T), was a part of the adapter. The adapter-ligated fragments were PCR-amplified (six cycles), quantified using Qubit (Invitrogen, Eugene, OR, USA) and subjected to target enrichment (SureSelect, Agilent). A pool of two samples (300 ng each) was used as input to one capture reaction and hybridized following SureSelect Target Enrichment for the Illumina Paired-End Sequencing Library Protocol. The enriched libraries were PCR-amplified (14 cycles) and confirmed to be of mean fragment size 430 bp. Purification steps were done using column-based (Qiagen) methods. Size selection was done using SPRI beads (Agencourt). All libraries were pooled and sequenced on a HiSeq 2000 instrument (Illumina) in the paired-end mode (2 × 101 bp).

+C: **Data processing**

After Illumina inherent purity filtering, demultiplexing and removal of the barcode, reads were aligned to the human genome assembly hg19, using Novoalign (Novocraft Technologies). Data were subjected to local realignment using GATK to minimize false-positive calls due to misalignment. Duplicates were marked using Picard (http://picard.sourceforge.net). Base quality scores were recalibrated to account for machine cycle and dinucleotide context. Mean target coverage for the libraries varied from ×198 to ×594. Variant calling was done using VarScan2 , by applying the VarScan2 'somatic' program to paired samtools mpileup output files from matched normal and tumour, using the 'strand-filter' option as recommended, to eliminate false-positive variants over-represented on either strand. For variant calling, minimum allowed coverage of ×8 in normal and ×6 in tumour was used. Only single nucleotide variants (SNVs)/indels, with minimum four variant allele-containing reads and over 10% variant allele frequency at the site, and < 3% variant allele frequency in the corresponding normal sample, were considered. Somatic SNVs were further filtered to remove clusters of SNVs and SNVs near indels, possibly caused by local misalignment. Additional filtering was applied as recommended to minimize the false-positive rate, with parameters leading to an average of 8% variants being filtered out. Due to heavy contamination by normal DNA (probably from the infiltrating lymphocytes), as witnessed by absence of mutations and copy number changes, M2 from P7, M1 from P20, M3 from P28 and M1 from P64 were excluded from the comparative analysis. The mutations for each patient were summarized and the unique mutations present only in a fraction of a patient’s analysed metastases were always subject to visual inspection in IGV. Variants were annotated using annovar . Mutations in the following genes were validated by Sanger sequencing: *PTEN*, *CDH1*, *GRIN2A*, *PIK3CA*, *HDAC4*, *RB1*, *RAD50*, *NUAK2*, *TTN*, *NKD2*, *FLT3*, *FLT4*, *SLC12A3*, *PRDM4*, *USP40* and *TSPAN4*. Copy number for tumour–normal pairs was derived from targeted exome data, using Contra . Contra was run with default settings and removing multi-mapped reads. Exons with < 200 reads in the corresponding blood sample were removed, as well as the upper percentile of exons with the highest read counts in blood. Data were segmented using GLAD .

+C: **Base composition analysis**

All SNVs were considered for analysis of base context of adjacent positions; tandem base substitutions were split into individual SNVs. Sequences with the coordinates –5 … +5 around the mutation site were derived from the hg19 reference. On the opposite strand, reverse complementary sequence was derived. For analysis of sequence context at random sites, coding bases in the gene panel, called 'reference' by VarScan, in blood and all tumours from the patient were randomly picked. Random A (*n =* 250) and T (*n =* 250) sites with coverage > 100 were investigated as above.

+B: Low-coverage whole-genome sequencing

+C: **Library preparation**

2 μg DNA was sheared to a broad range of fragment sizes, with mode of 700–800 bp, using Covaris, with the following settings: duty cycle, 5; intensity, 3; cycles/burst, 200; time, 40 s. Adapters were ligated to the end-repaired, adenylated and phosphorylated fragments. Each sample was labelled with a unique six nucleotide-long barcode that, together with the seventh base (ligation-T), was a part of the adapter. The adapter-ligated fragments were PCR amplified (six cycles) and quantified using Qubit (Invitrogen, Eugene, OR, USA). Purification steps were done using column-based methods (Qiagen). Fragment size selection was done using SPRI beads (Agencourt). Mean fragment size of the libraries varied (580–890 bp). All libraries were mixed and the pool was sequenced on a HiSeq 2000 instrument (Illumina).

+C: **Data processing**

After the purity filtering, demultiplexing and barcode removal, each library yielded 31–70 million 2 × 94 bp read pairs. Reads were mapped to hg19 human genome using bwa , amounting to haploid coverage between ×1.9 and ×4.2. Duplicates were marked using Picard (http://picard.sourceforge.net). Genome-wide copy numbers were derived using Control-FREEC . Control-FREEC was run with default settings in 50 kb windows, using the corresponding blood sample as control. Structural variation was assessed using BreakDancer , with the following parameters: at least two supporting read pairs for tumour, one for normal; a minimum score of 30 for tumour, 10 for normal. The list of the structural variants in the normal was subtracted from the tumour list with a 10 kb flanking region tolerance for the overlap. Interchromosomal rearrangements (CTX) between break points that coincided with a copy number change were evaluated visually in IGV and subjected to validation by PCR amplification of the fusion point and Sanger sequencing (five rearrangements). Validation was successful for four out of five rearrangements.

For immunohistochemical analysis of MITF expression, 2 μm sections were automatically pretreated using the PT-link system (Dako, Glostrup, Denmark) and then stained in an Autostainer Plus (Dako) using a monoclonal MITF-antibody (clone C5, Sigma-Aldrich, St. Louis, MO, USA; diluted 1:50).

+A: **Supplementary references**

1. Harbst K, Staaf J, Lauss M, *et al.* Molecular profiling reveals low- and high-grade forms of primary melanoma. *Clin Cancer Res* 2012; **18**: 4026–4036.

2. Forbes SA, Bhamra G, Bamford S, *et al.* The Catalogue of Somatic Mutations in Cancer (COSMIC). *Curr Protoc Hum Genet* 2008; **10**: Unit 10.11.

3. McKenna A, Hanna M, Banks E, *et al.* The Genome Analysis Toolkit: a MapReduce framework for analyzing next-generation DNA sequencing data. *Genome Res* 2010; **20**: 1297–1303.

4. Koboldt DC, Zhang Q, Larson DE, *et al.* VarScan 2: somatic mutation and copy number alteration discovery in cancer by exome sequencing. *Genome Res* 2012; **22**: 568–576.

5. Wang K, Li M, Hakonarson H. ANNOVAR: functional annotation of genetic variants from high-throughput sequencing data. *Nucleic Acids Res* 2010; **38**: e164.

6. Li J, Lupat R, Amarasinghe KC, *et al.* CONTRA: copy number analysis for targeted resequencing. *Bioinformatics* 2012; **28**: 1307–1313.

7. Hupe P, Stransky N, Thiery JP, *et al.* Analysis of array CGH data: from signal ratio to gain and loss of DNA regions. *Bioinformatics* 2004; **20**: 3413–3422.

8. Li H, Durbin R. Fast and accurate short read alignment with Burrows–Wheeler transform. *Bioinformatics* 2009; **25**: 1754–1760.

9. Boeva V, Popova T, Bleakley K, *et al.* Control-FREEC: a tool for assessing copy number and allelic content using next-generation sequencing data. *Bioinformatics* 2012; **28**: 423–425.

10. Boeva V, Zinovyev A, Bleakley K, *et al.* Control-free calling of copy number alterations in deep-sequencing data using GC-content normalization. *Bioinformatics* 2011; **27**: 268–269.

11. Chen K, Wallis JW, McLellan MD, *et al.* BreakDancer: an algorithm for high-resolution mapping of genomic structural variation. *Nat Methods* 2009; **6**: 677–681.

12. Robinson JT, Thorvaldsdottir H, Winckler W, *et al.* Integrative genomics viewer. *Nat Biotechnol* 2011; **29**: 24–26.
