# Supplementary material for: Molecular and genetic diversity in the metastatic process of melanoma
Source: J Pathol. 2014 Jan 27;233(1):39–50. doi: 10.1002/path.4318 (PMC4359751; doi:10.1002/path.4318)

A)

Unsupervised hierarchical clustering using top 75% most variable genes

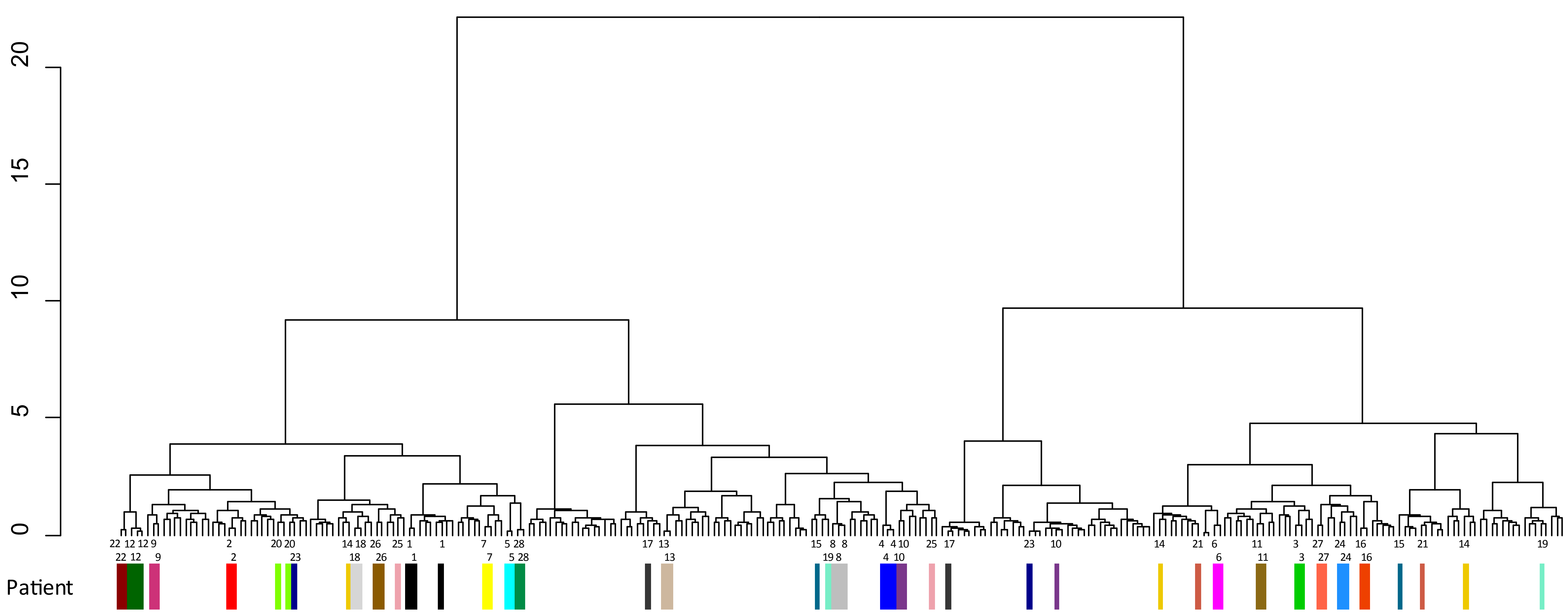

B)

Hierarchical clustering using the centroid genes

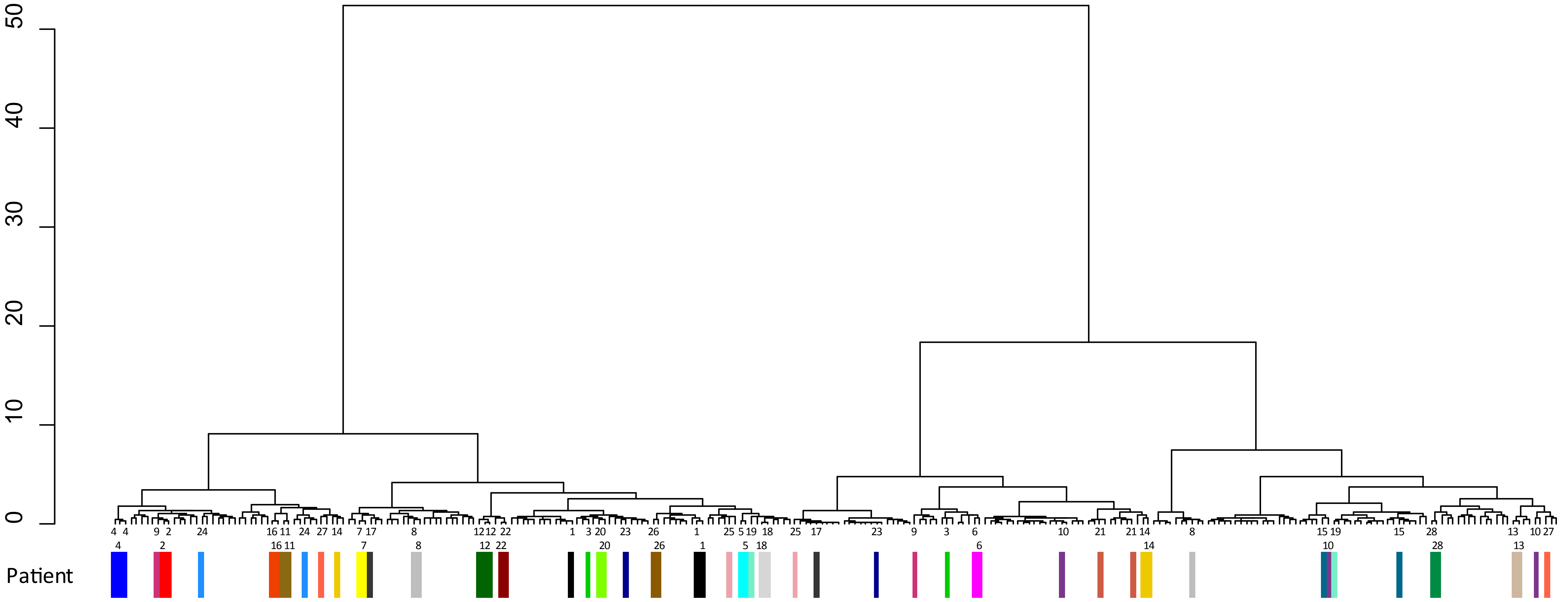

Supplement: Supplementary file 2 — Hierarchical clustering of melanoma samples. (A) Unsupervised clustering using the 75% most variable genes. (B) Clustering using the centroid genes (n = 500). Genes were taken from centroids defined in the Harbst et al study 1. Hierarchical clustering was performed using Pearson correlation and Ward as a distance measure [file path0233-0039-sd2.pdf]
