# Supplementary material for: Molecular and genetic diversity in the metastatic process of melanoma
Source: J Pathol. 2014 Jan 27;233(1):39–50. doi: 10.1002/path.4318 (PMC4359751; doi:10.1002/path.4318)

Pat 1 M1

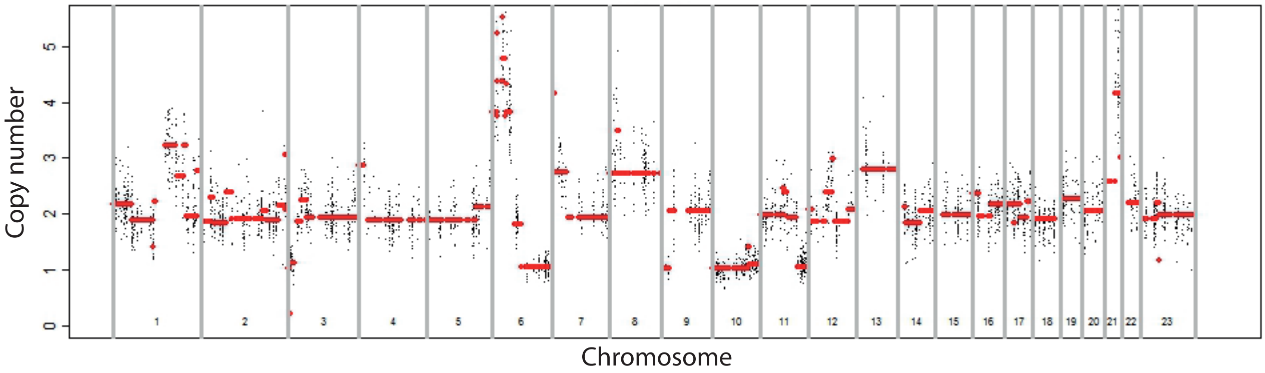

Pat 1 M2

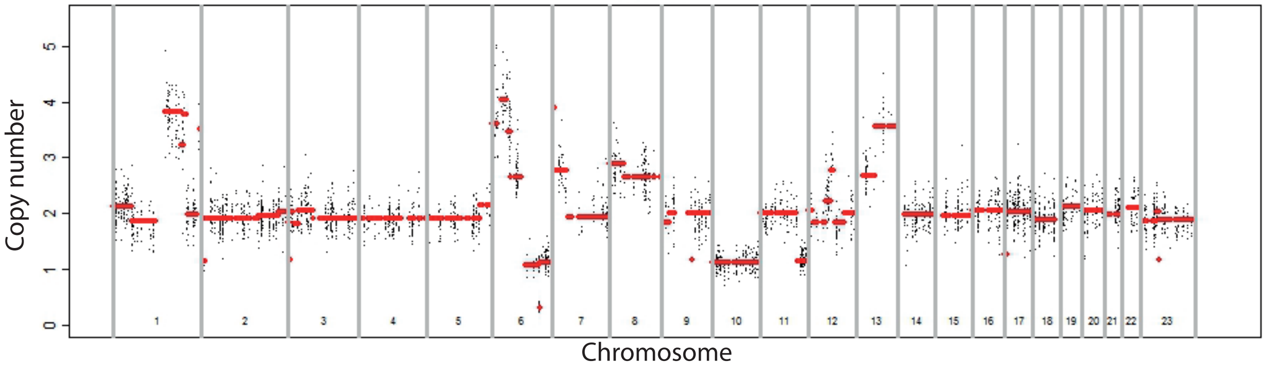

Pat 1 M3

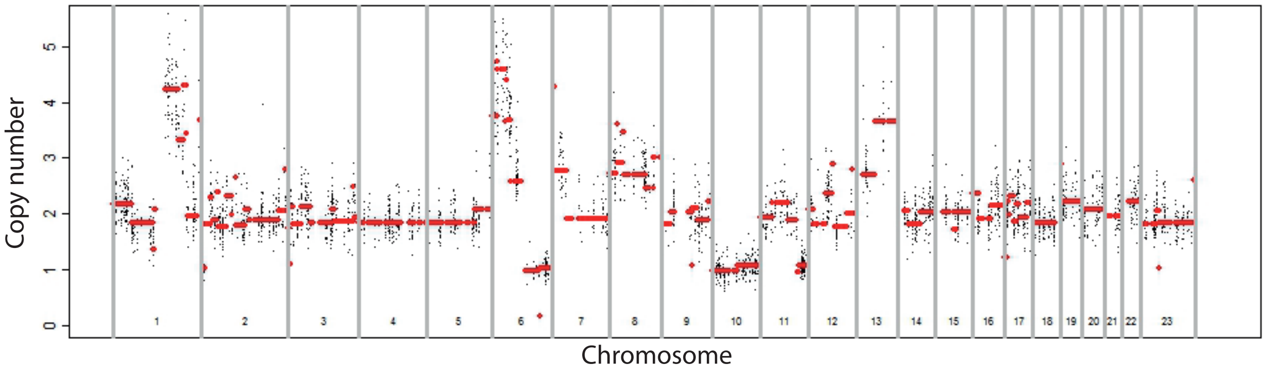

Pat 2 M1

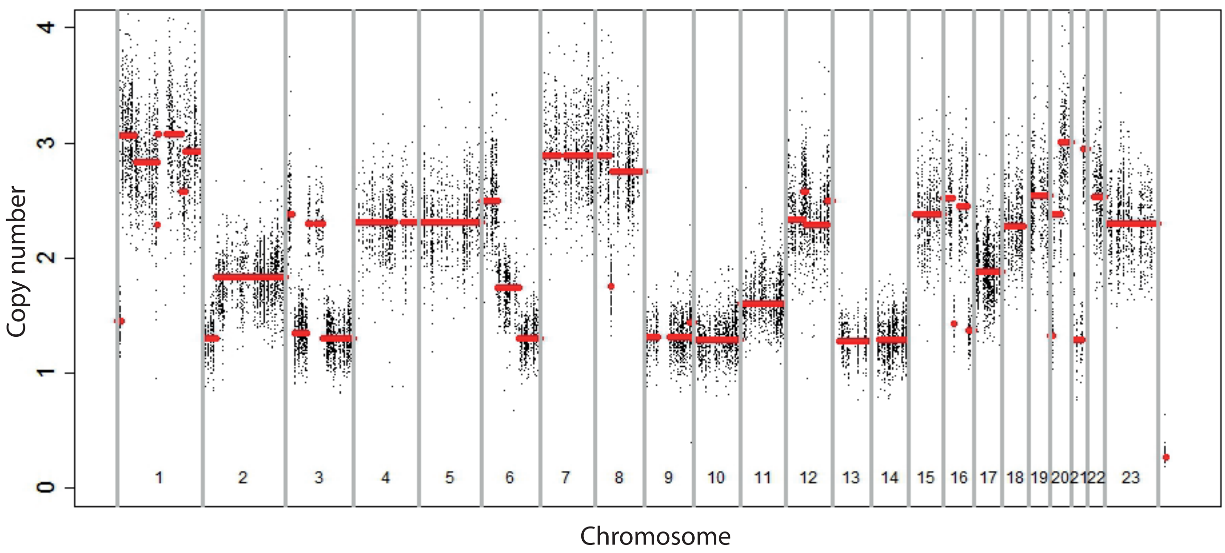

Pat 2 M2

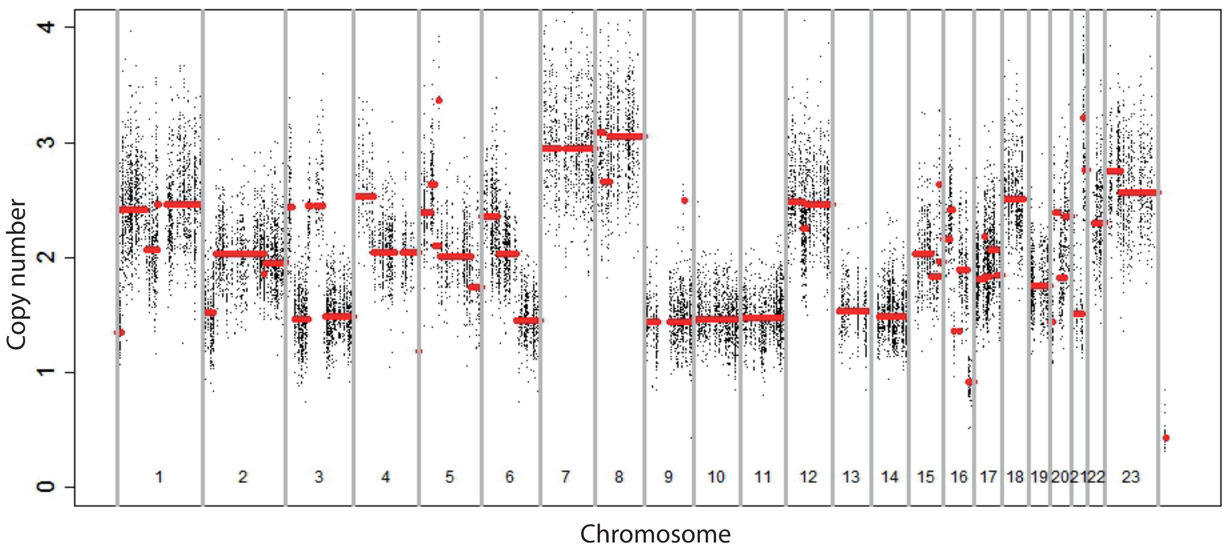

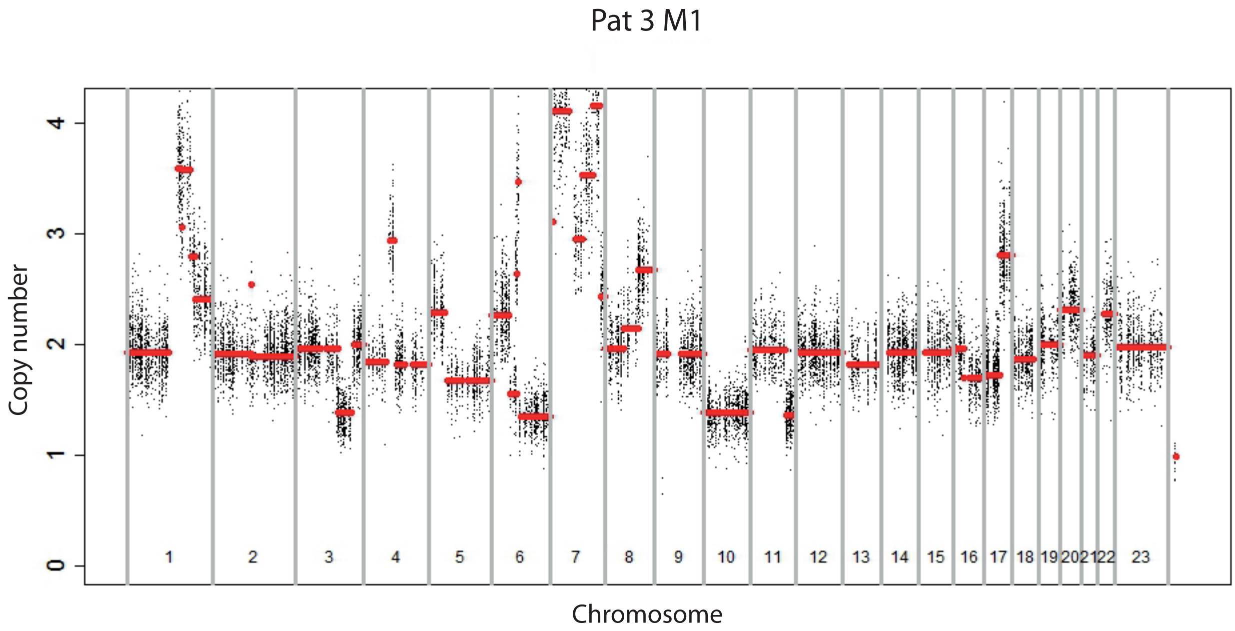
Pat 3 M1
Copy number
0
1
2
3
4
1
2
3
4
5
6
7
8
9
10
11
12
13
14
15
16
17
18
19
20
21
22
23
Chromosome

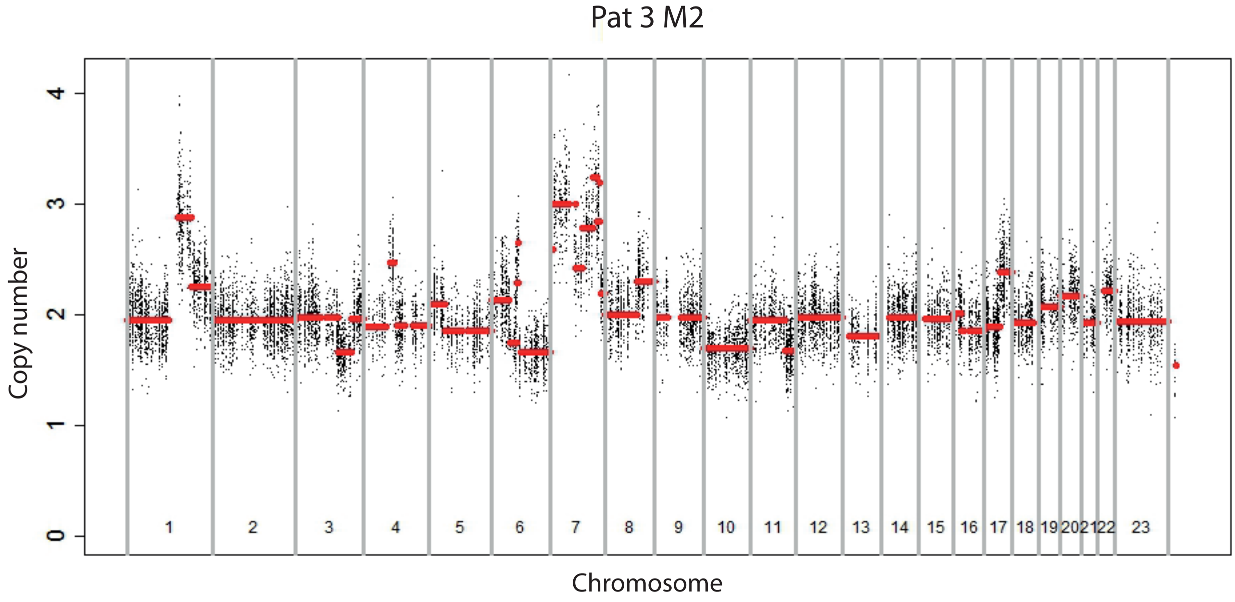
Pat 3 M2
Copy number
0
1
2
3
4
1
2
3
4
5
6
7
8
9
10
11
12
13
14
15
16
17
18
19
20
21
22
23
Chromosome

Pat 4 M1

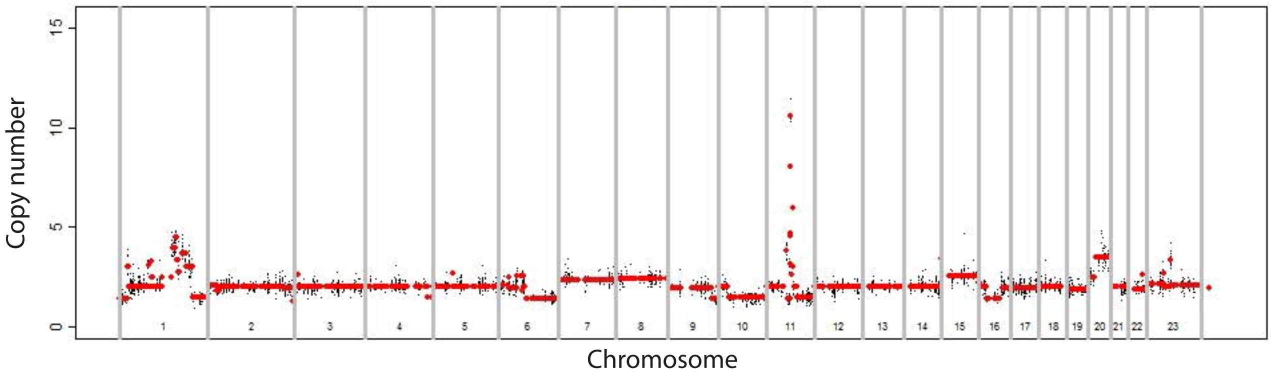

Pat 4 M2

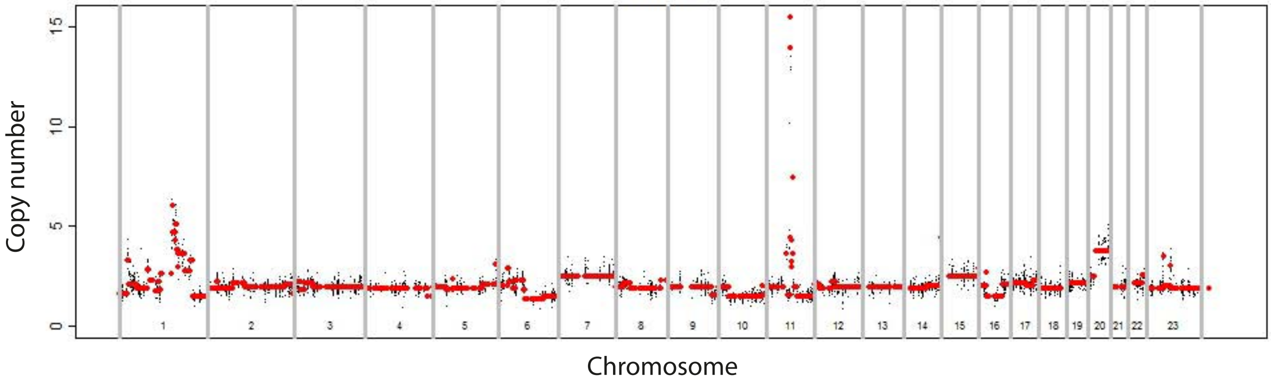

Pat 4 M3

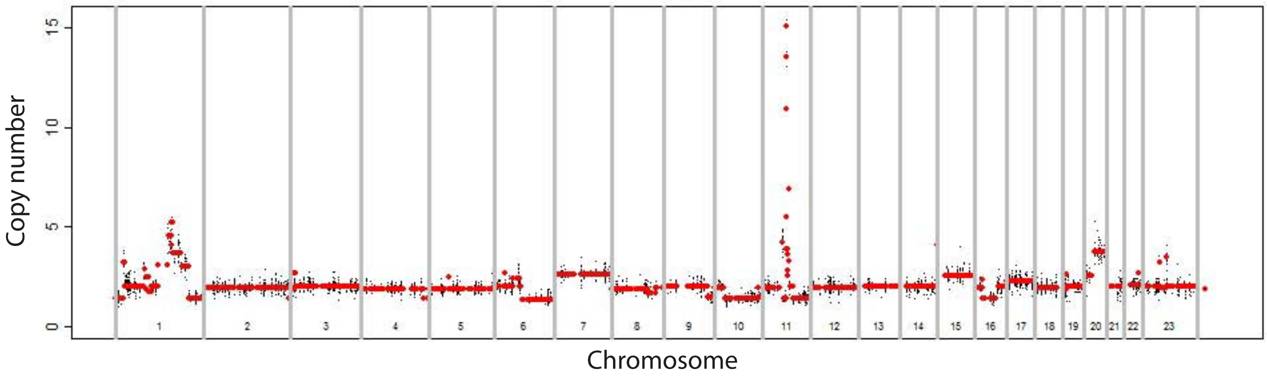

Pat 5 M1

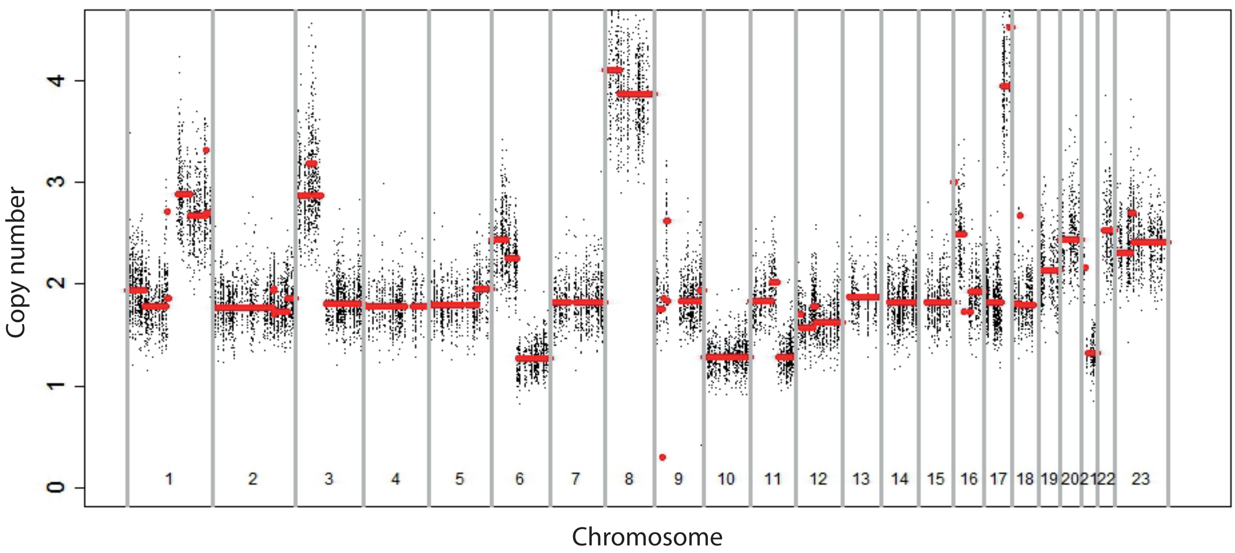

Pat 5 M2

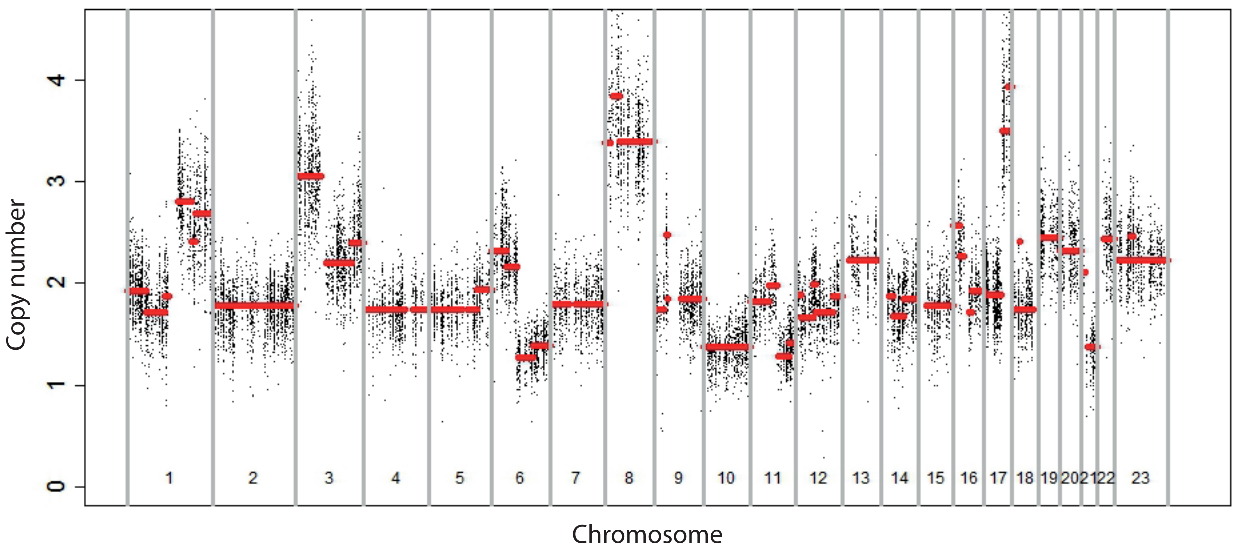

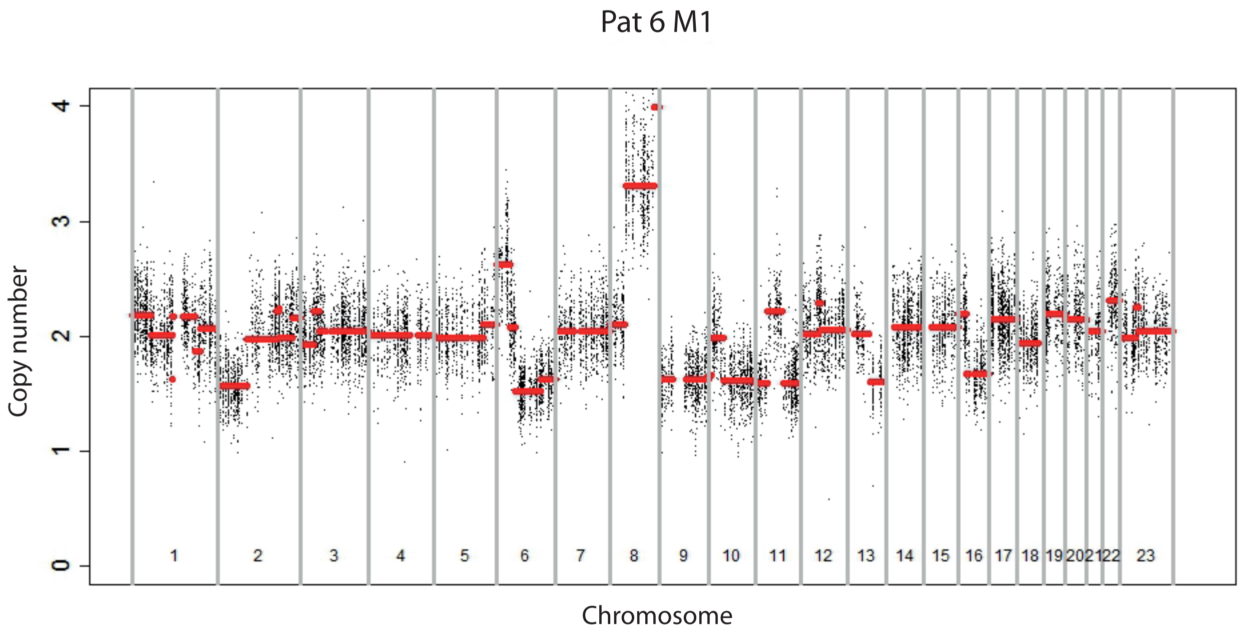
Pat 6 M1
Copy number
0
1
2
3
4
1
2
3
4
5
6
7
8
9
10
11
12
13
14
15
16
17
18
19
20
21
22
23
Chromosome

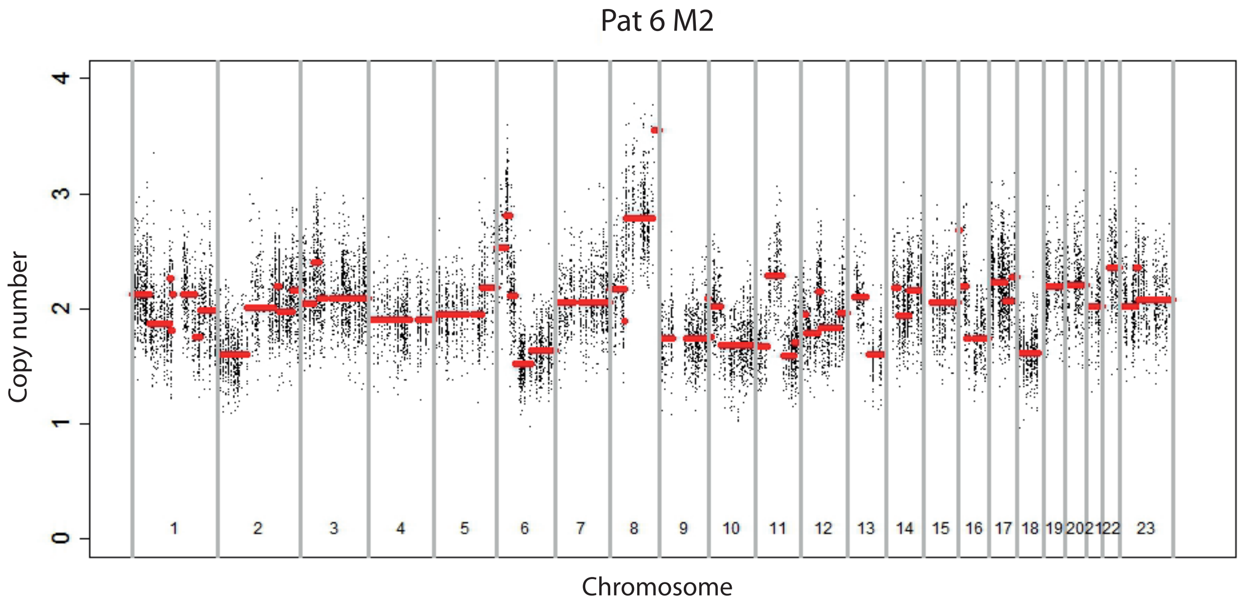
Pat 6 M2
Copy number
0
1
2
3
4
1
2
3
4
5
6
7
8
9
10
11
12
13
14
15
16
17
18
19
20
21
22
23
Chromosome

Pat 7 M1

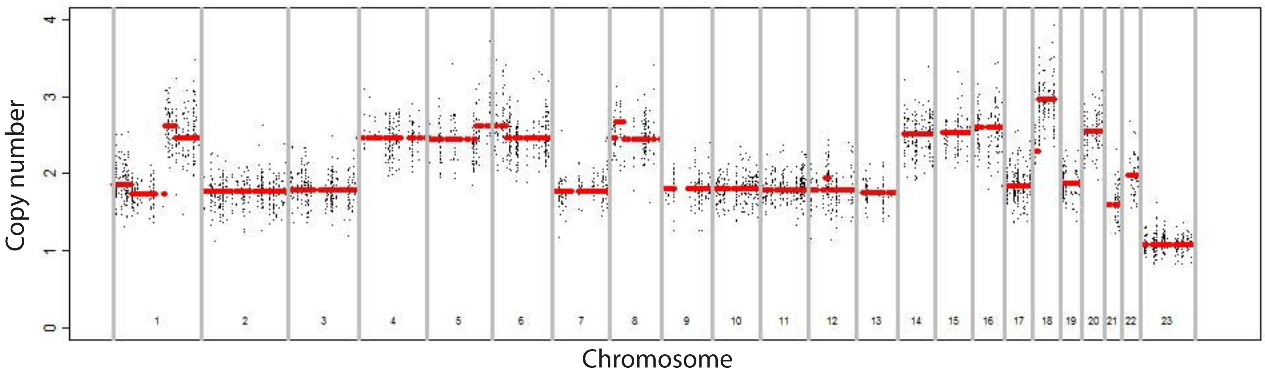

Pat 7 M2

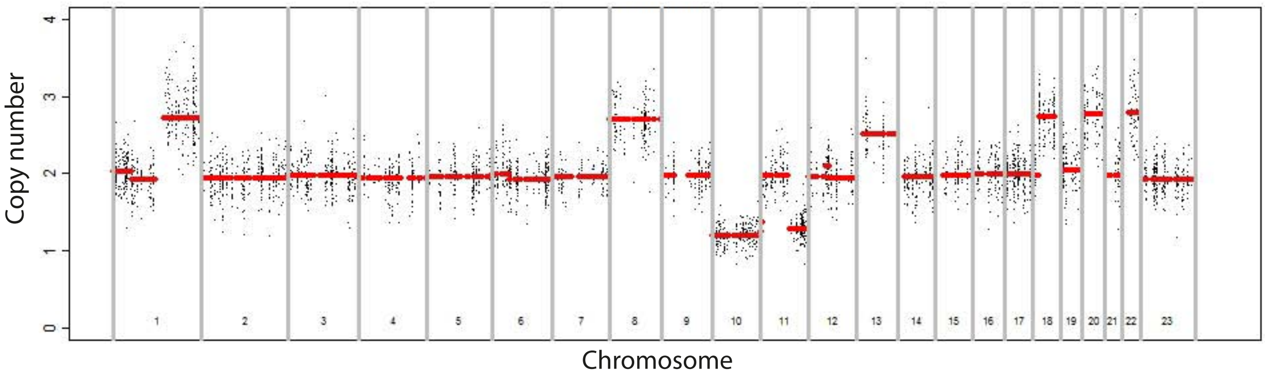

Pat 8 M1

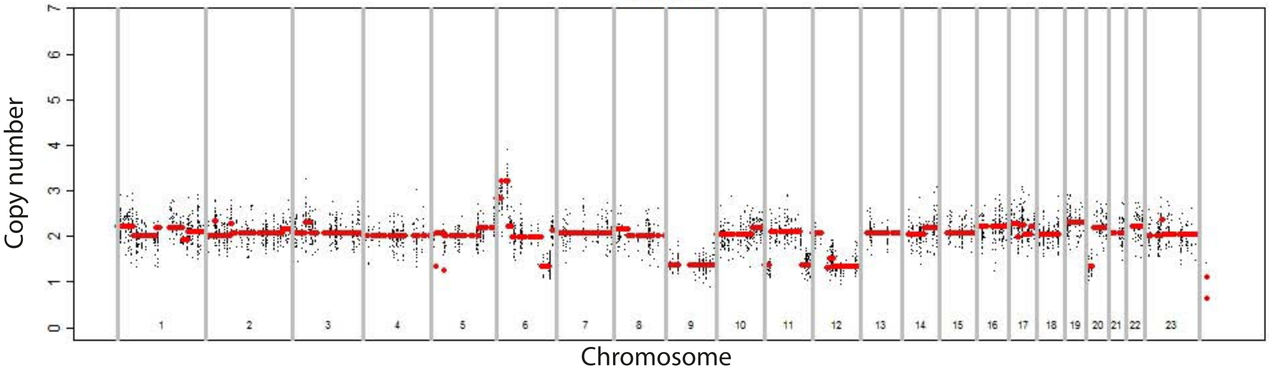

Pat 8 M2

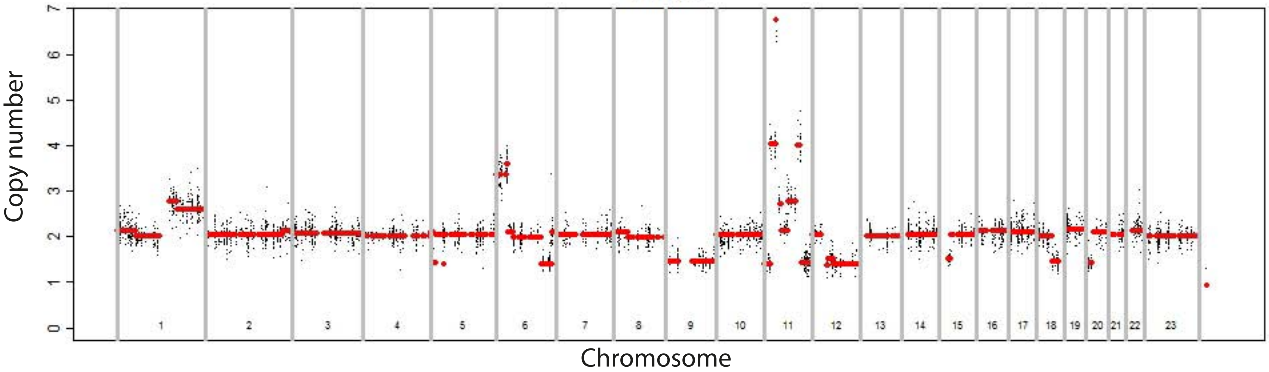

Pat 8 M3

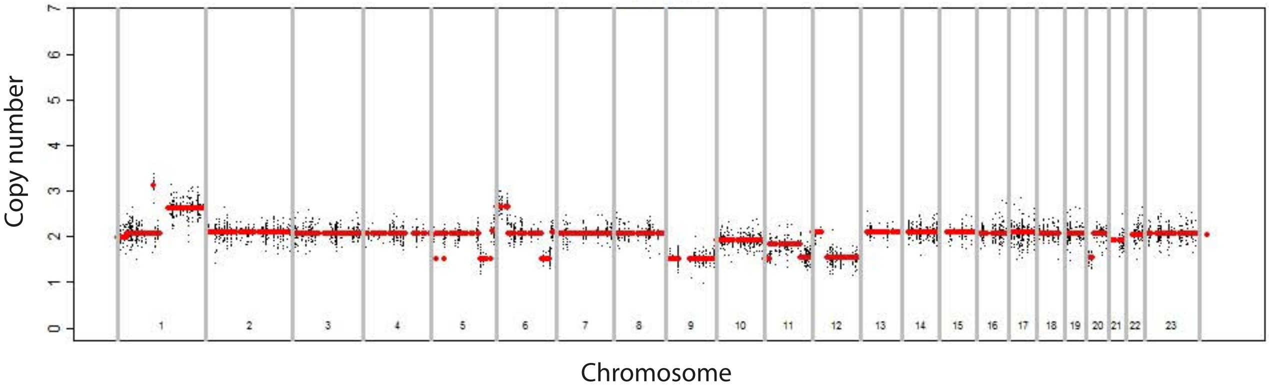

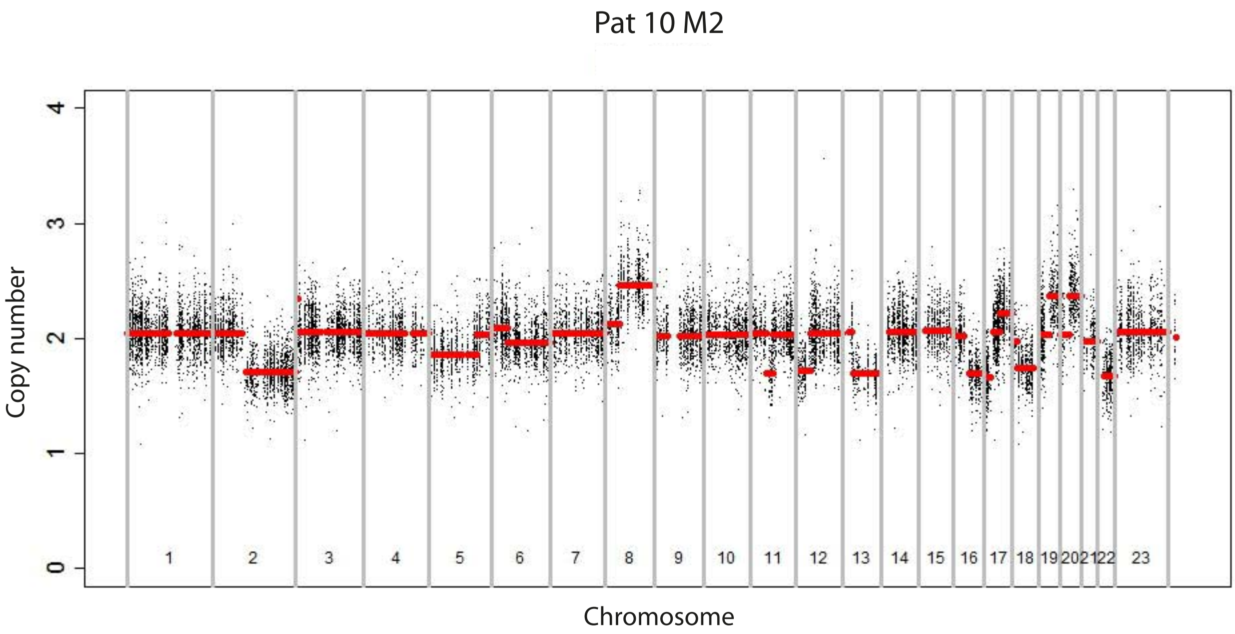
Pat 10 M2
Copy number
0
1
2
3
4
1
2
3
4
5
6
7
8
9
10
11
12
13
14
15
16
17
18
19
20
21
22
23
Chromosome

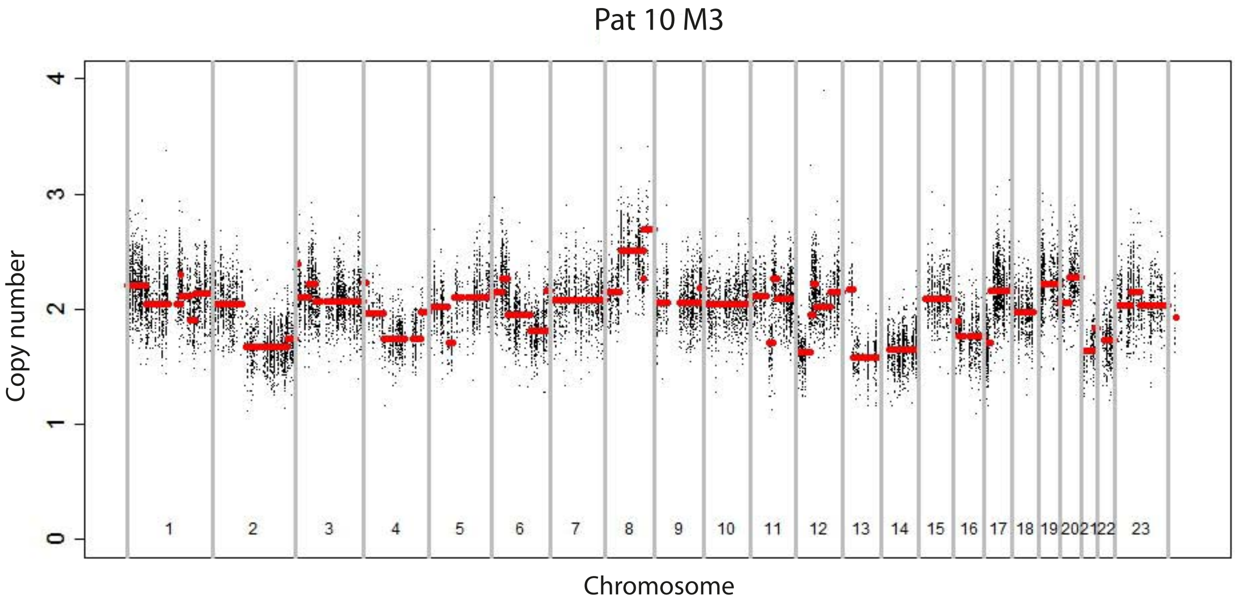
Pat 10 M3
Copy number
0
1
2
3
4
1
2
3
4
5
6
7
8
9
10
11
12
13
14
15
16
17
18
19
20
21
22
23
Chromosome

Pat 11 M1

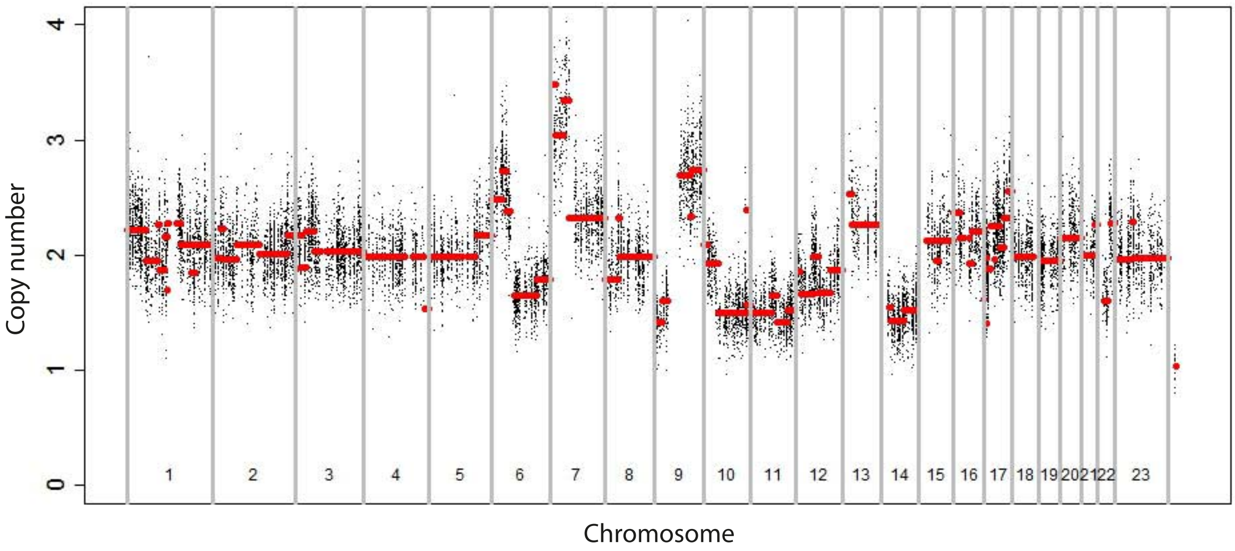

Pat 11 M2

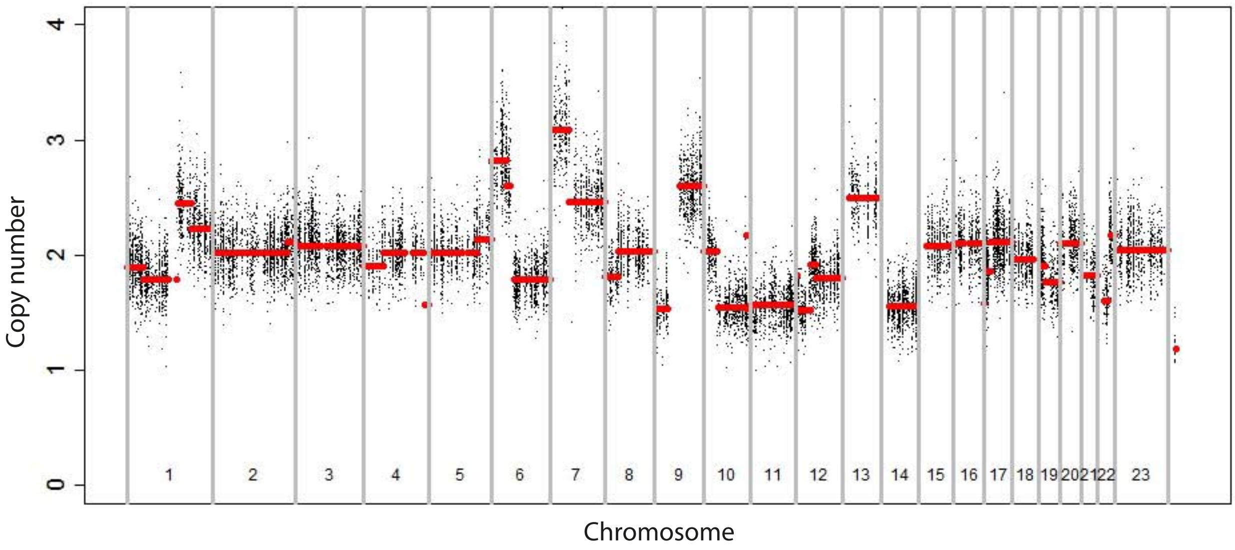

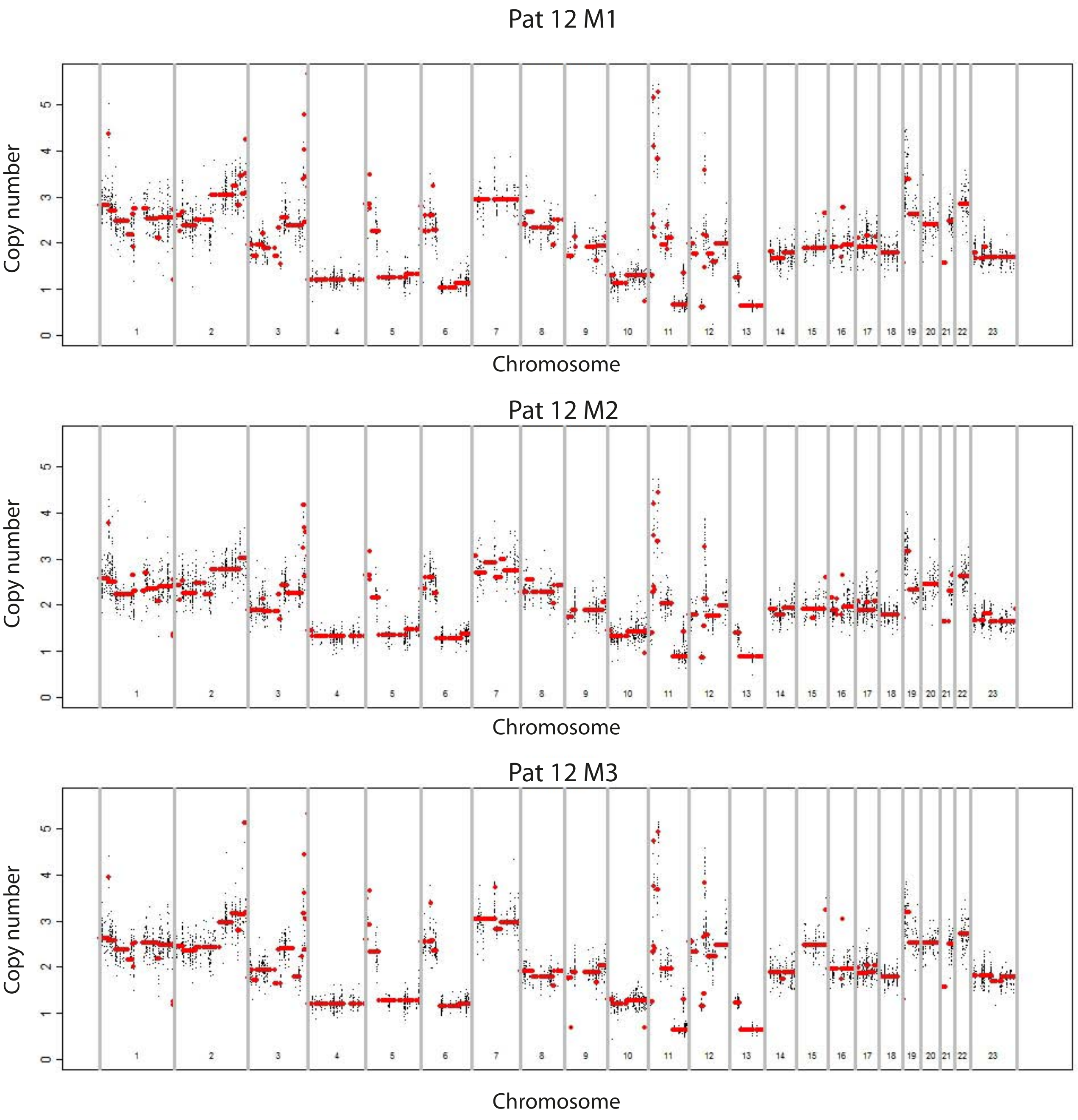
Pat 12 M1
Copy number
0 1 2 3 4 5
1 2 3 4 5 6 7 8 9 10 11 12 13 14 15 16 17 18 19 20 21 22 23
Chromosome
Pat 12 M2
Copy number
0 1 2 3 4 5
1 2 3 4 5 6 7 8 9 10 11 12 13 14 15 16 17 18 19 20 21 22 23
Chromosome
Pat 12 M3
Copy number
0 1 2 3 4 5
1 2 3 4 5 6 7 8 9 10 11 12 13 14 15 16 17 18 19 20 21 22 23
Chromosome

Pat 14 M1

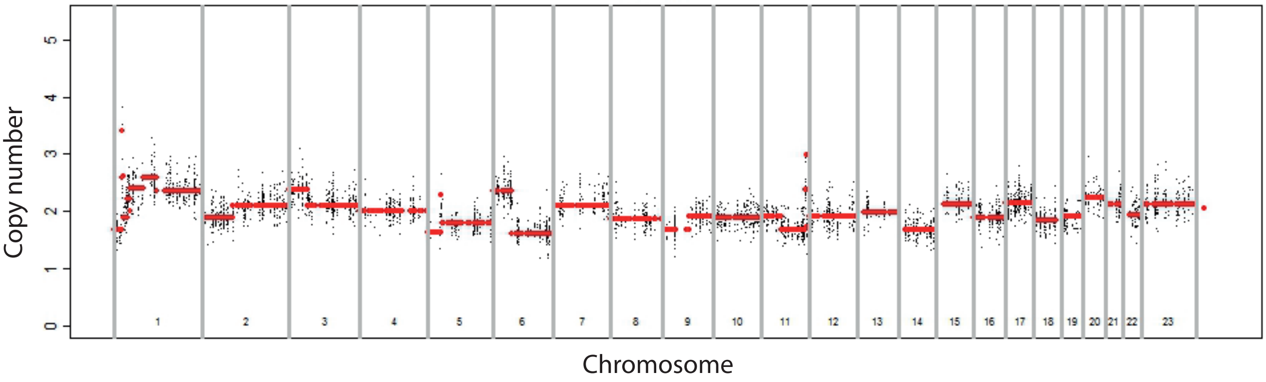

Pat 14 M2

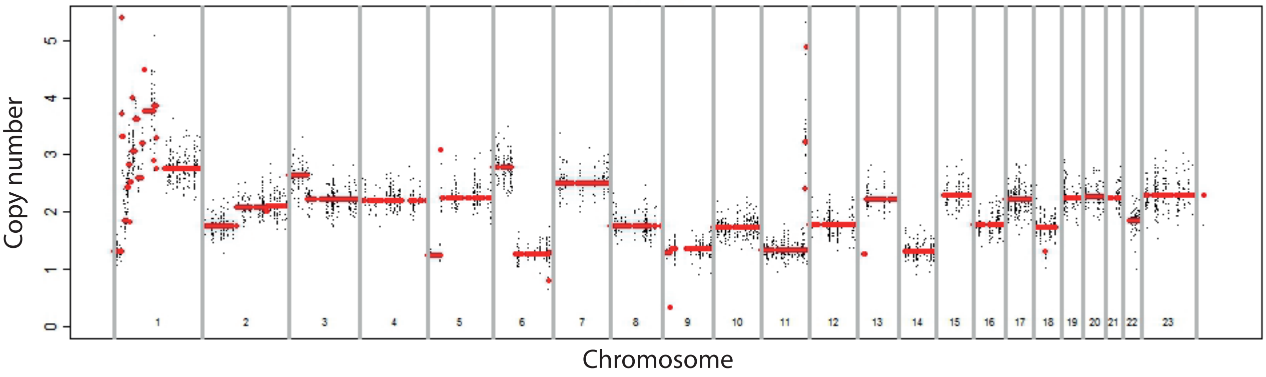

Pat 14 M3

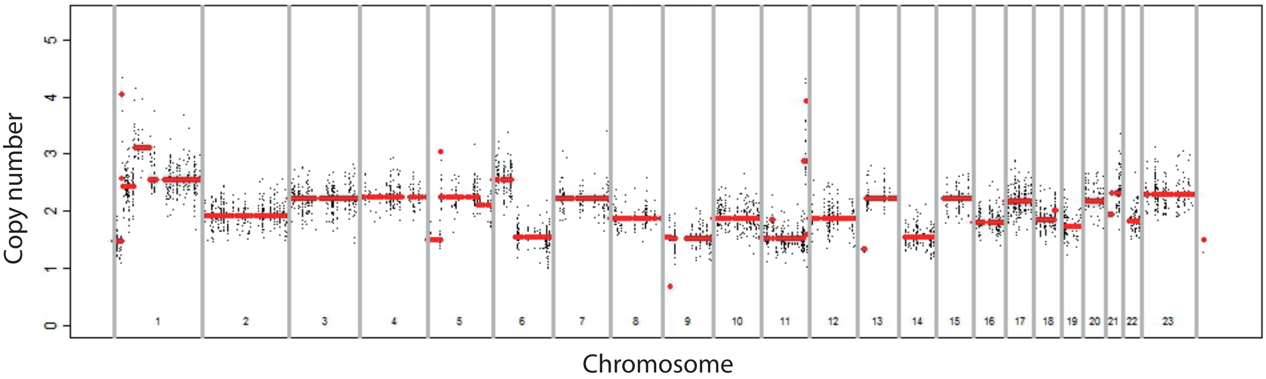

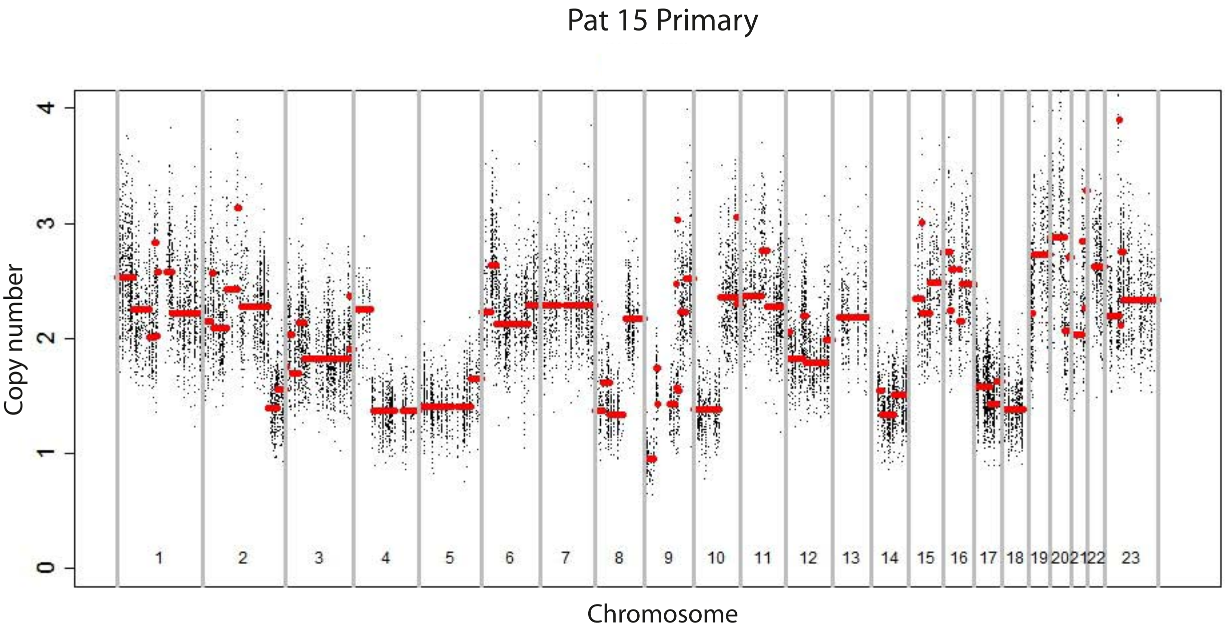
Pat 15 Primary
Copy number
0
1
2
3
4
1
2
3
4
5
6
7
8
9
10
11
12
13
14
15
16
17
18
19
20
21
22
23
Chromosome

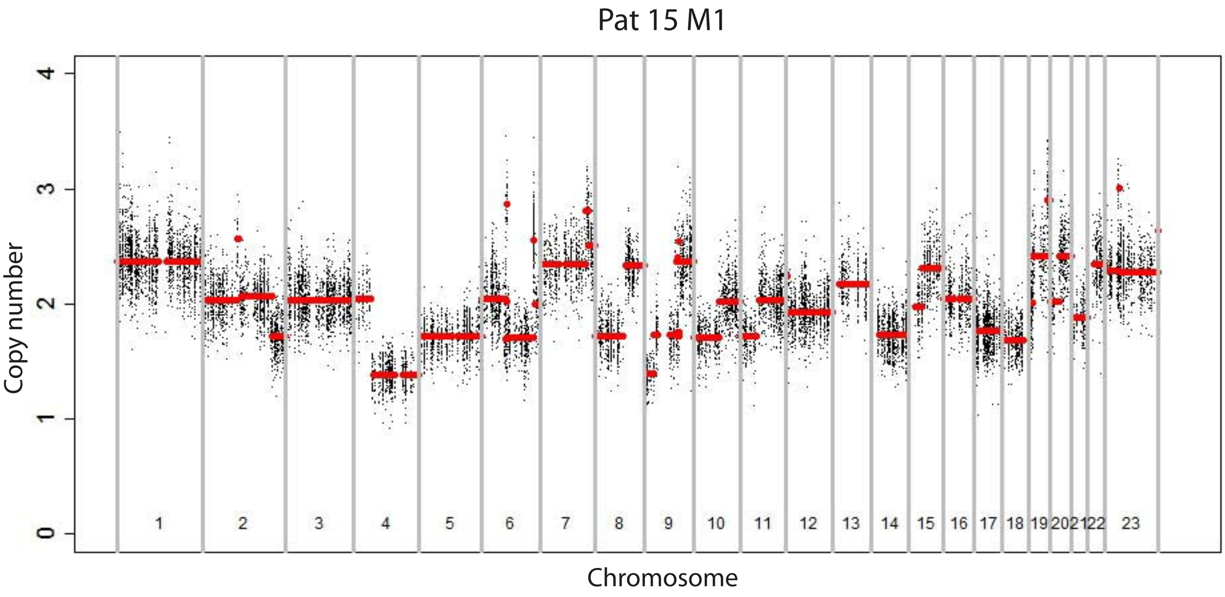
Pat 15 M1
Copy number
0
1
2
3
4
1
2
3
4
5
6
7
8
9
10
11
12
13
14
15
16
17
18
19
20
21
22
23
Chromosome

Pat 16 M1

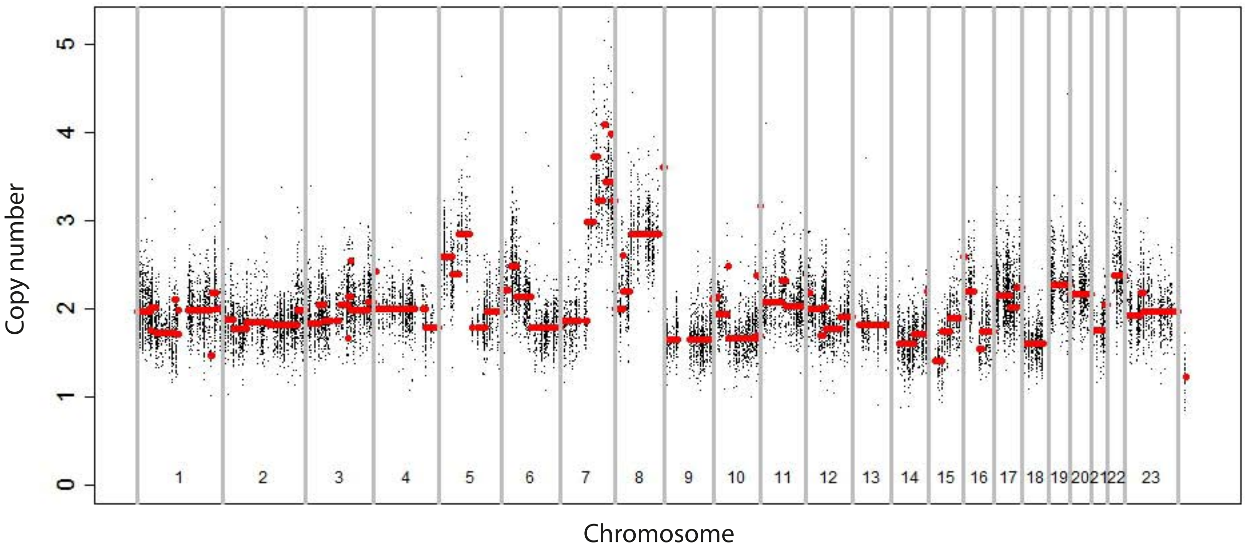

Pat 16 M2

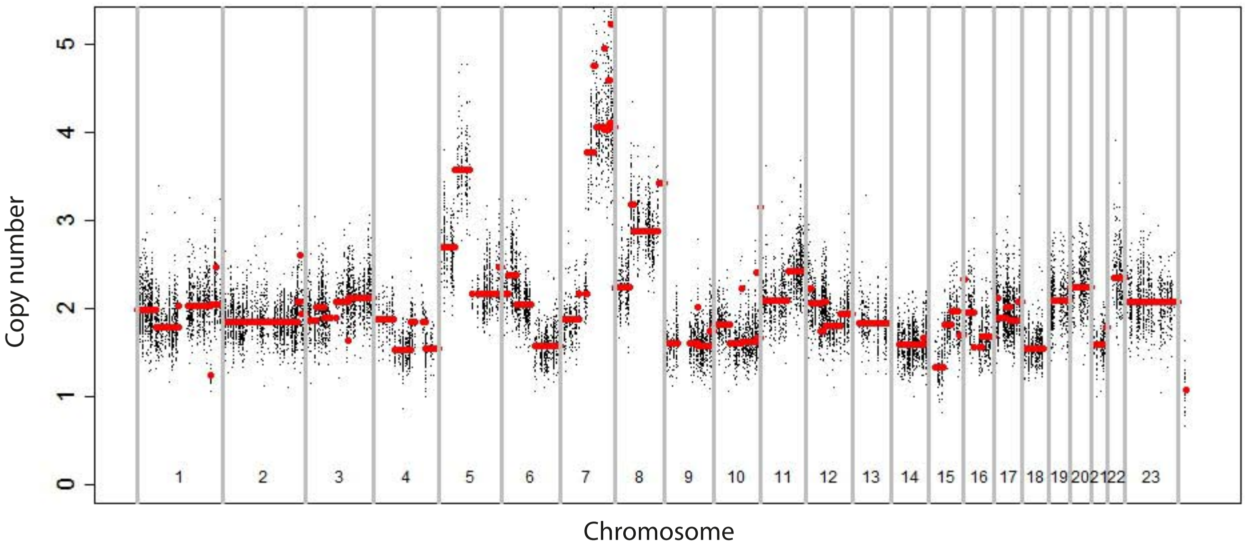

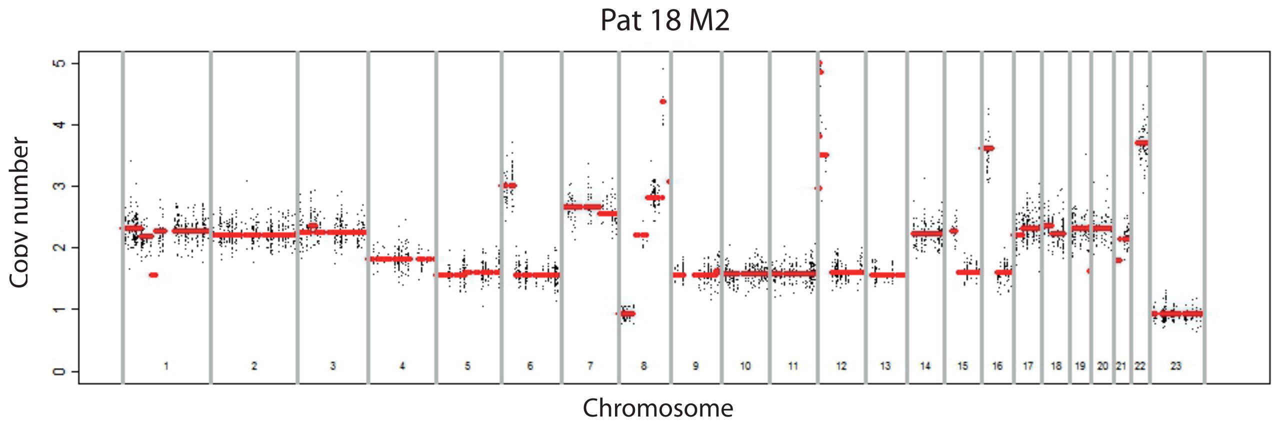

Pat 18 M2
Copy number
0
1
2
3
4
5
1 2 3 4 5 6 7 8 9 10 11 12 13 14 15 16 17 18 19 20 21 22 23
Chromosome

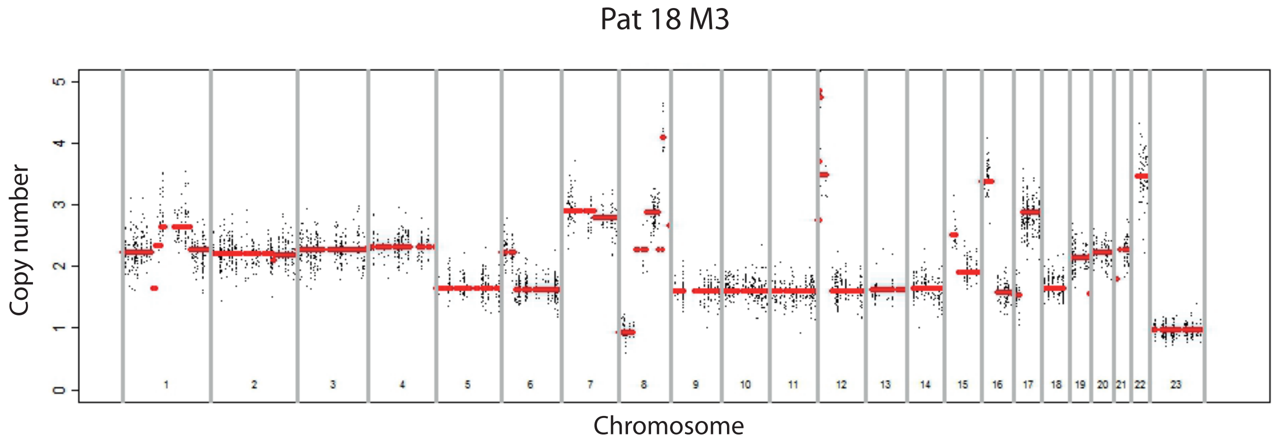

Pat 18 M3
Copy number
0
1
2
3
4
5
1 2 3 4 5 6 7 8 9 10 11 12 13 14 15 16 17 18 19 20 21 22 23
Chromosome

Pat 19 M1

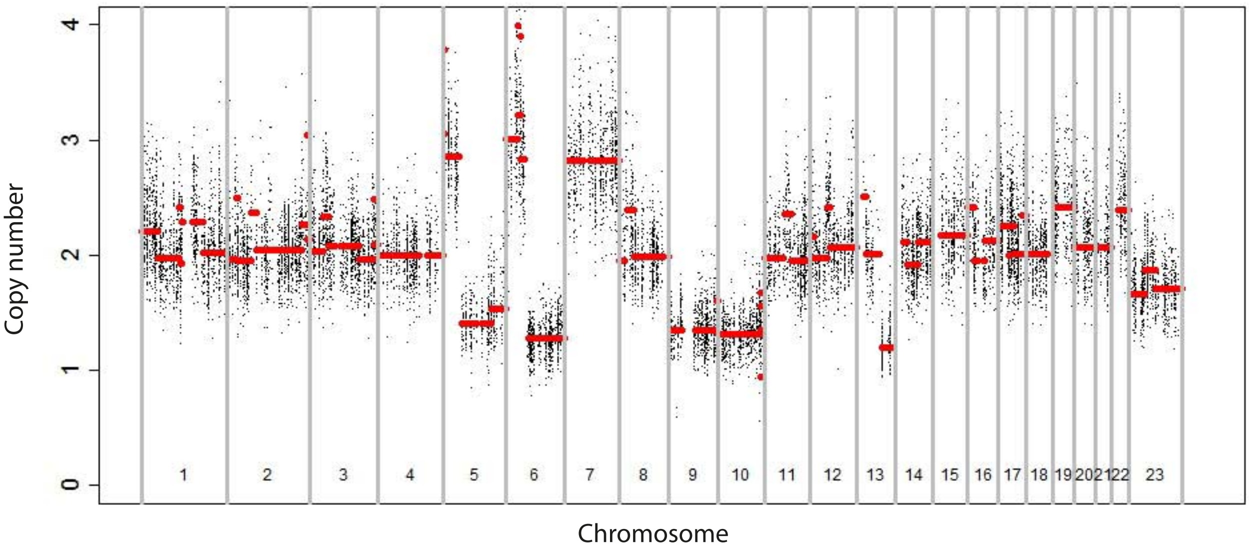

Pat 19 M2

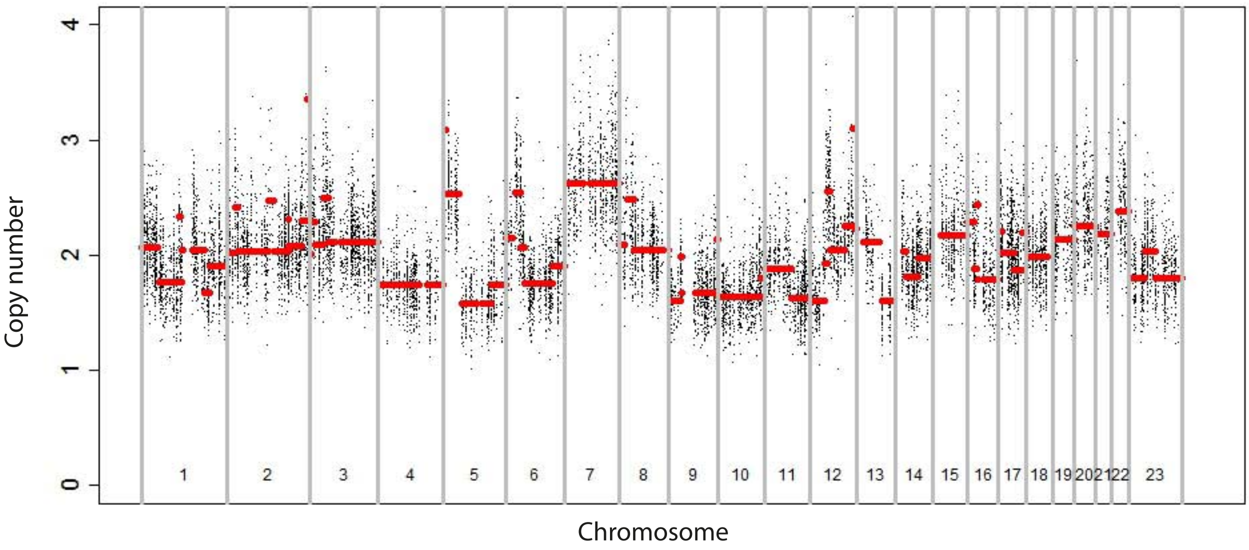

Pat 20 M1

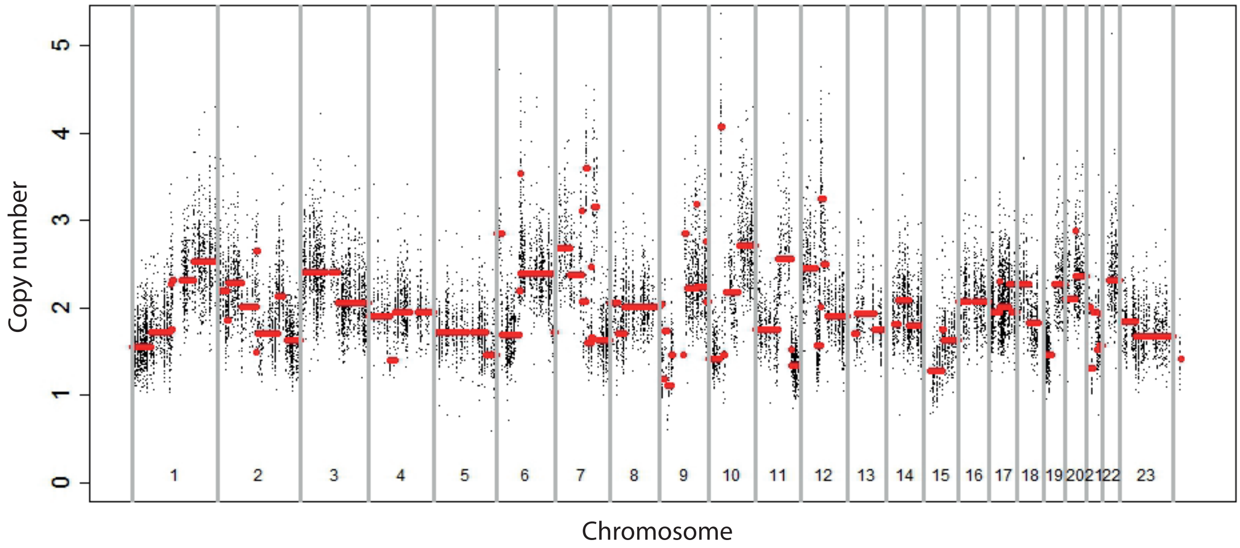

Pat 20 M2

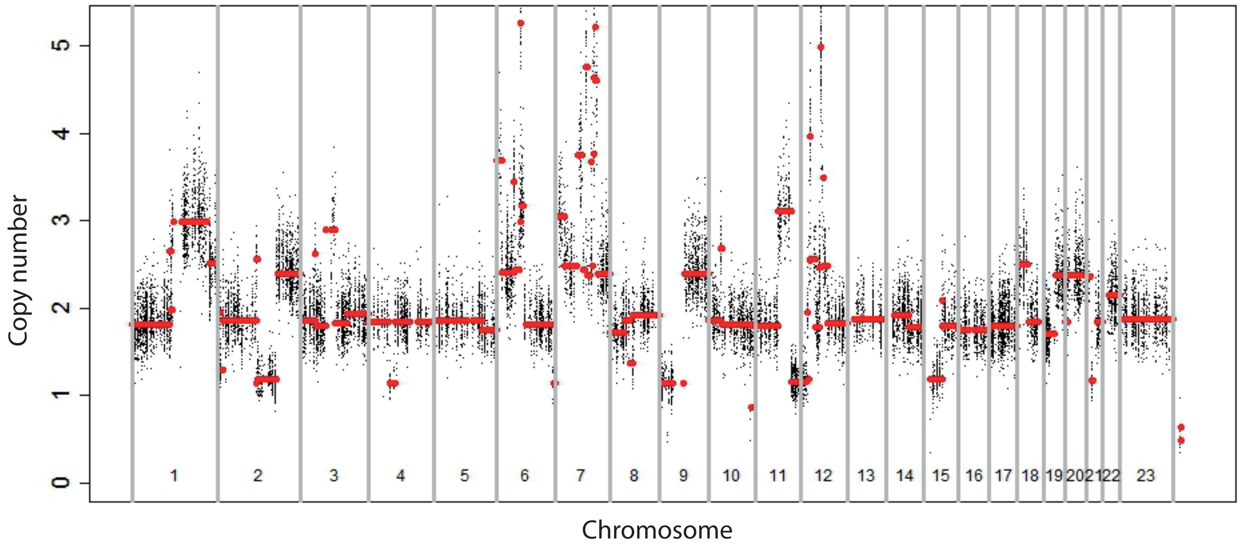

Pat 22 M1

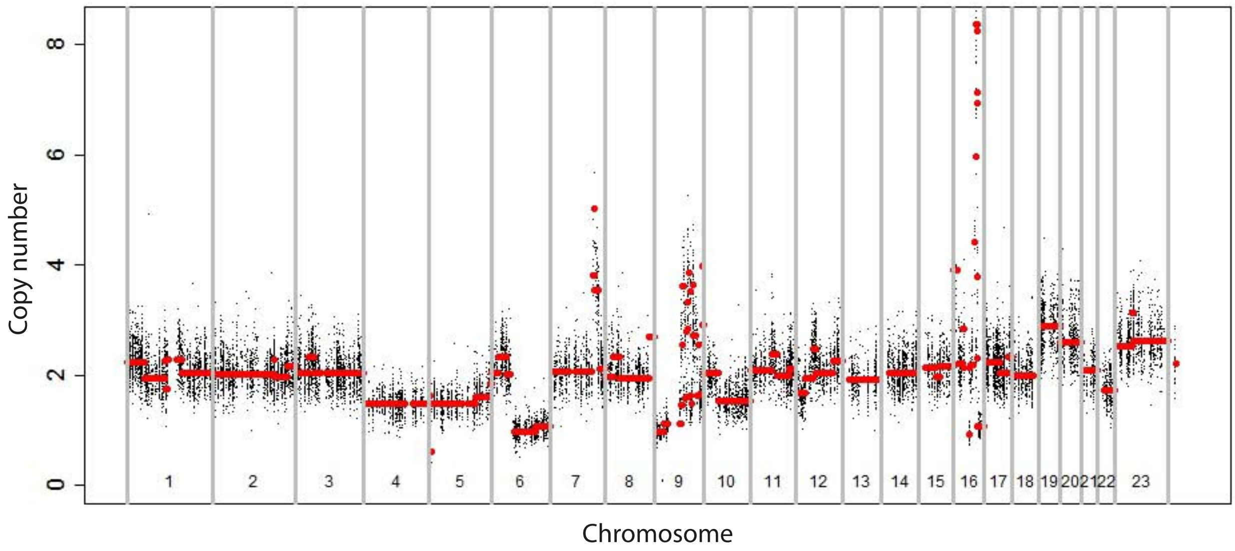

Pat 22 M2

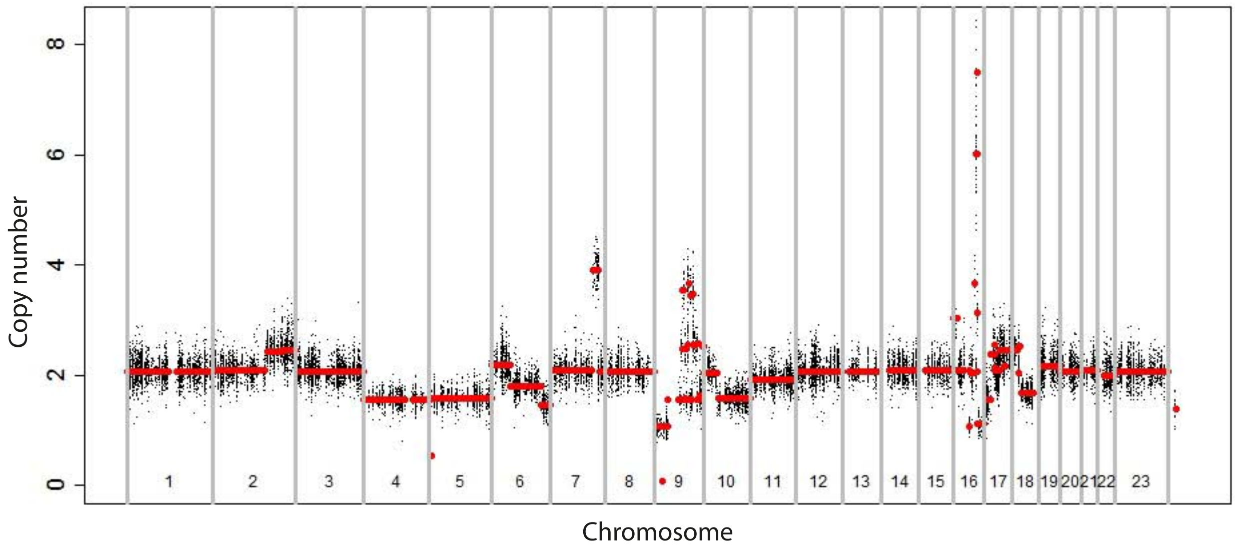

Pat 25 M1

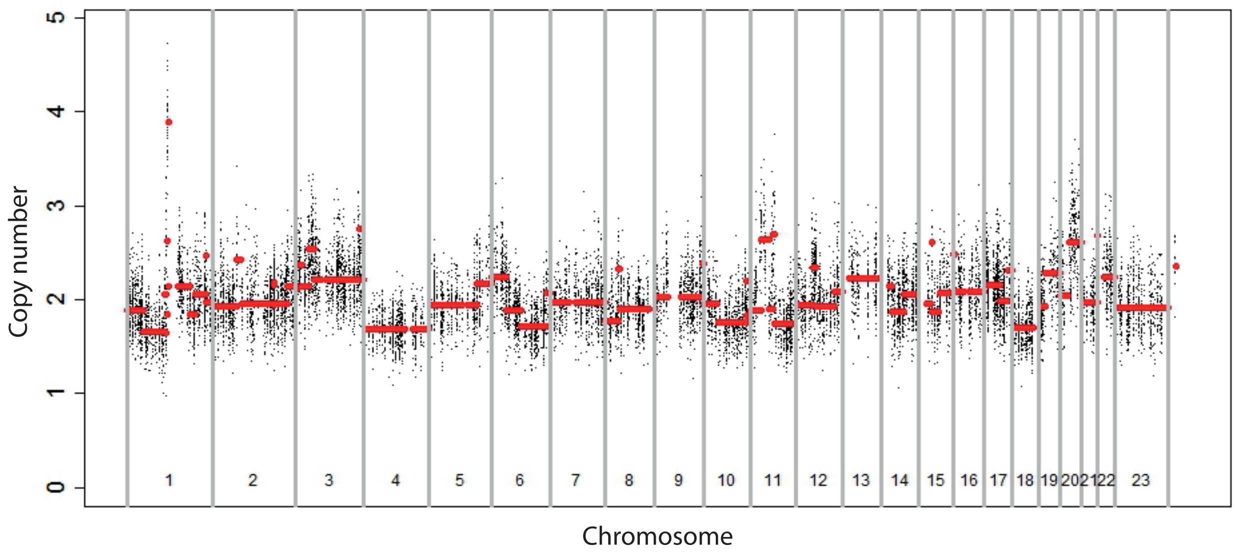

Pat 25 M2

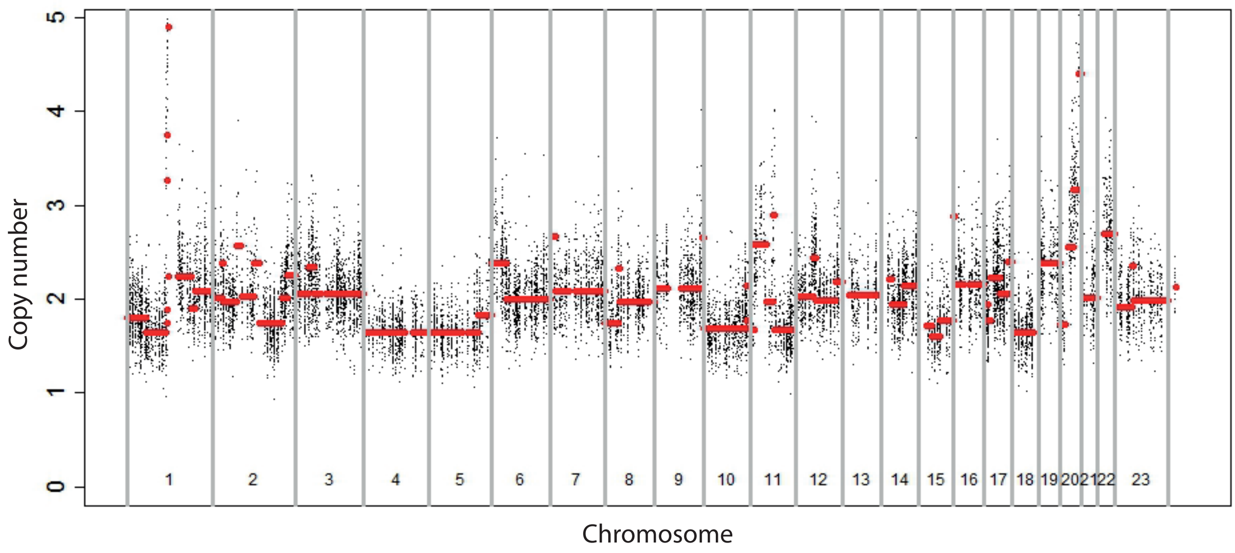

Pat 26 M1

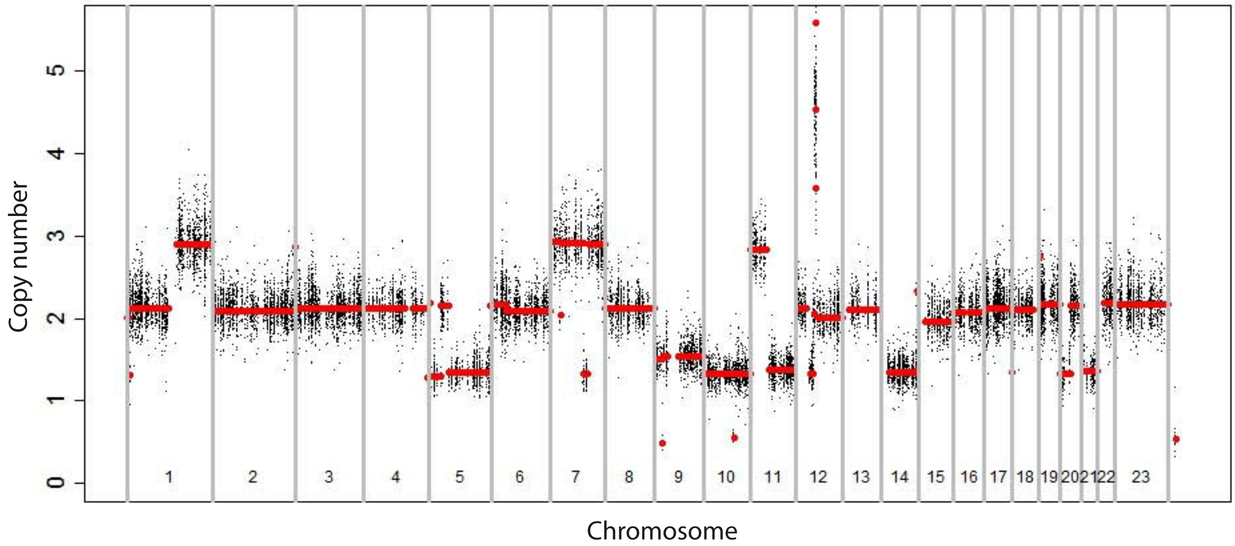

Pat 26 M2

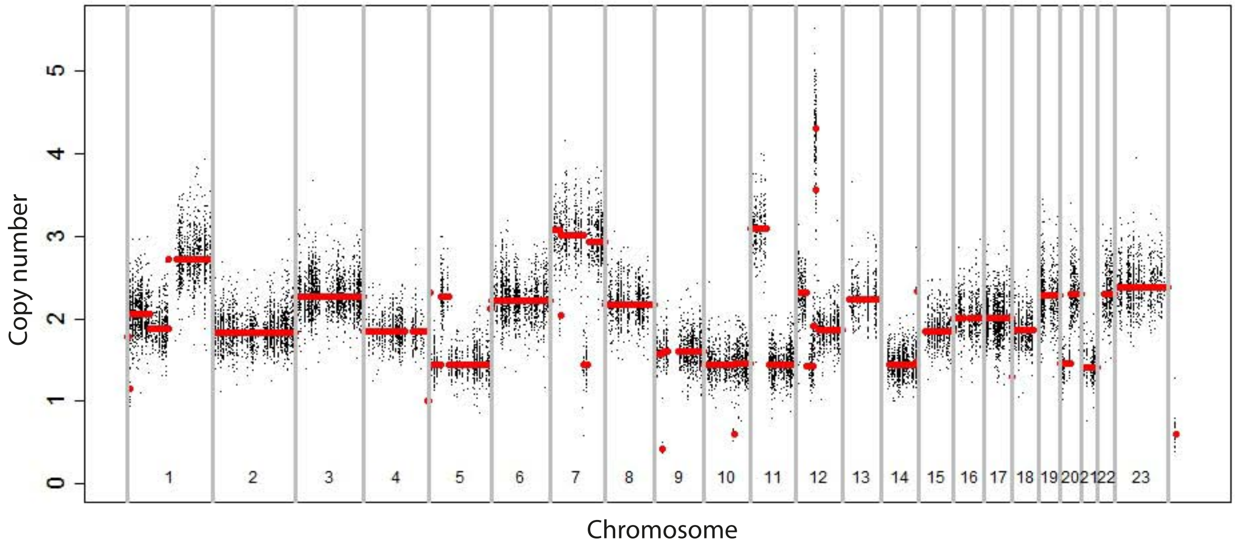

Pat 27 M1

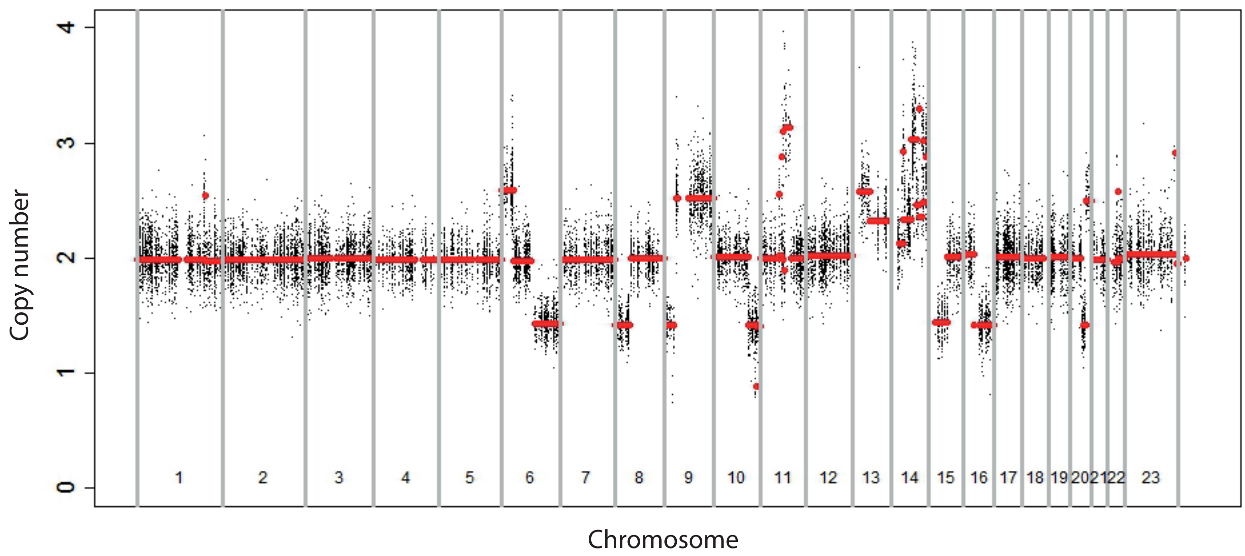

Pat 27 M2

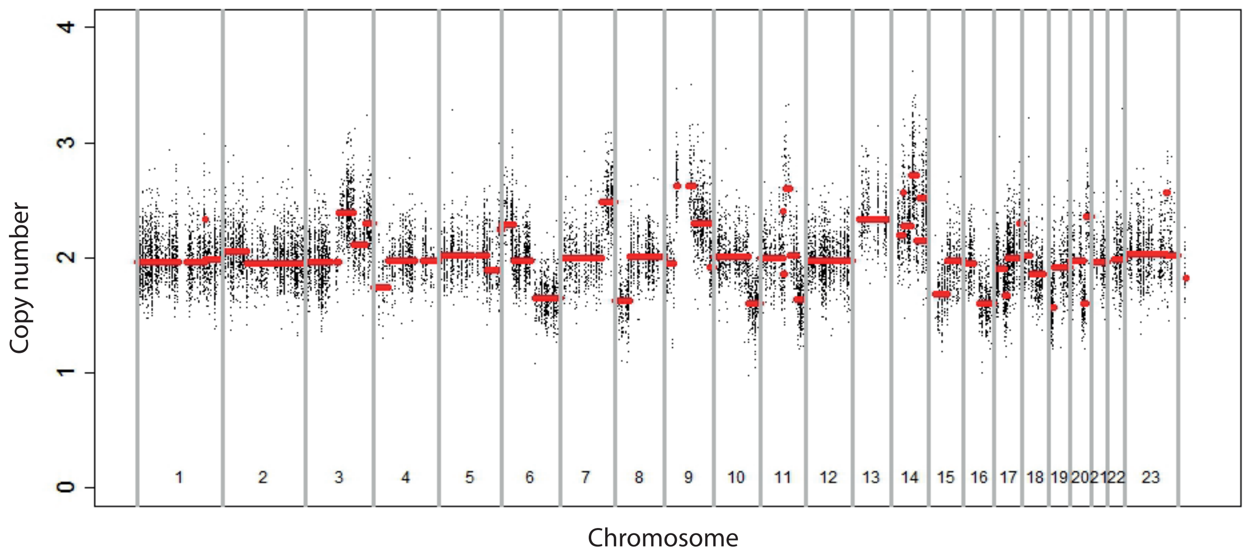

Pat 28 M1

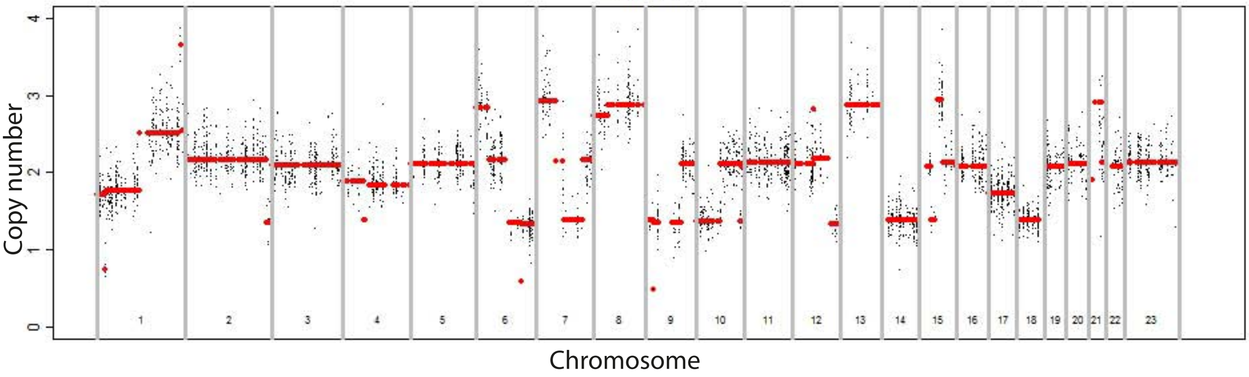

Pat 28 M2

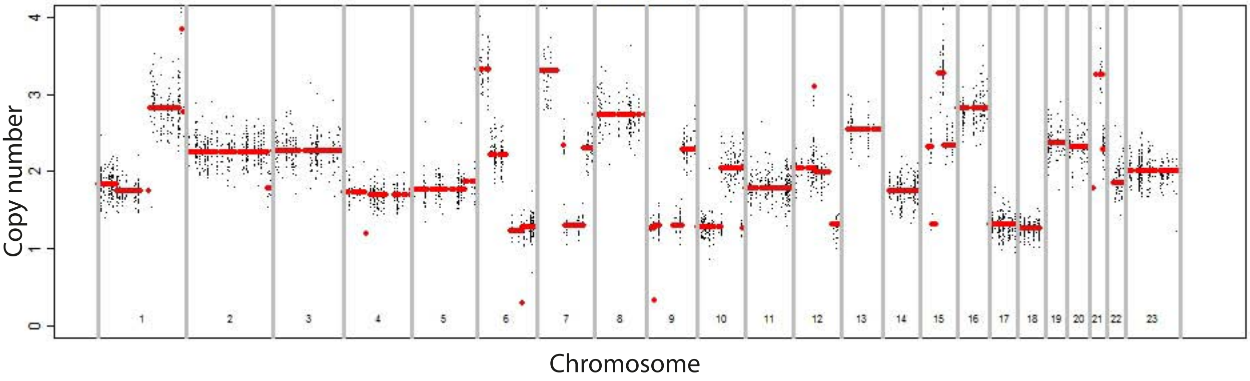

Supplement: Supplementary file 4 — Copy number profiles of the melanoma metastatic tumours (different tumours of the same patient are indicated as M1, M2, etc.), based on CONTRA analysis of the targeted deep-sequencing data. Segmentation was performed using gain and loss analysis of DNA (GLAD). Segments defined by GLAD are shown in red [file path0233-0039-sd4.pdf]
