# Supplementary figures and images for: Molecular and genetic diversity in the metastatic process of melanoma
Source: J Pathol. 2014 Jan 27;233(1):39–50. doi: 10.1002/path.4318 (PMC4359751; doi:10.1002/path.4318)

**A)**

## Patient 1

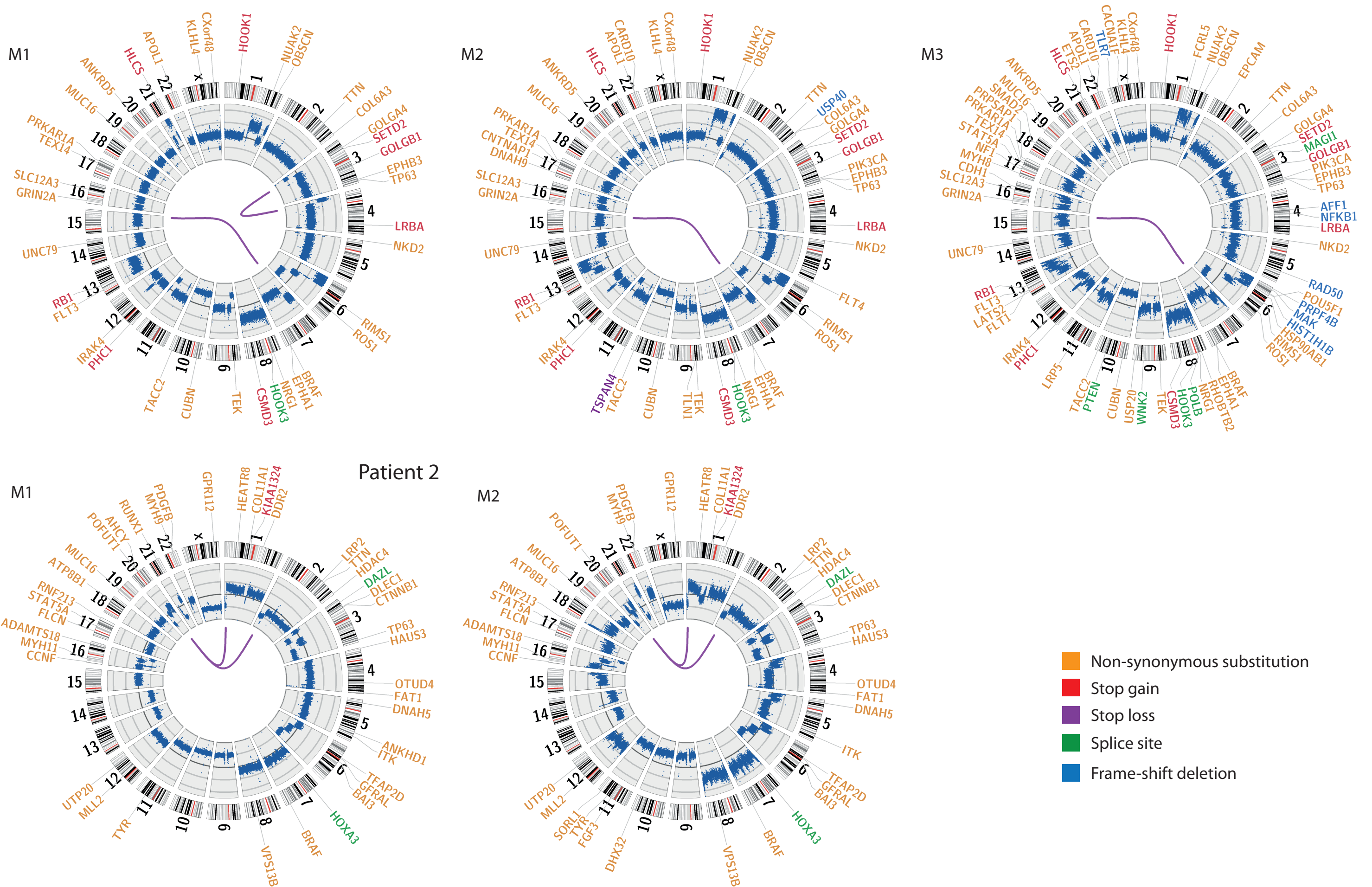

**B)**

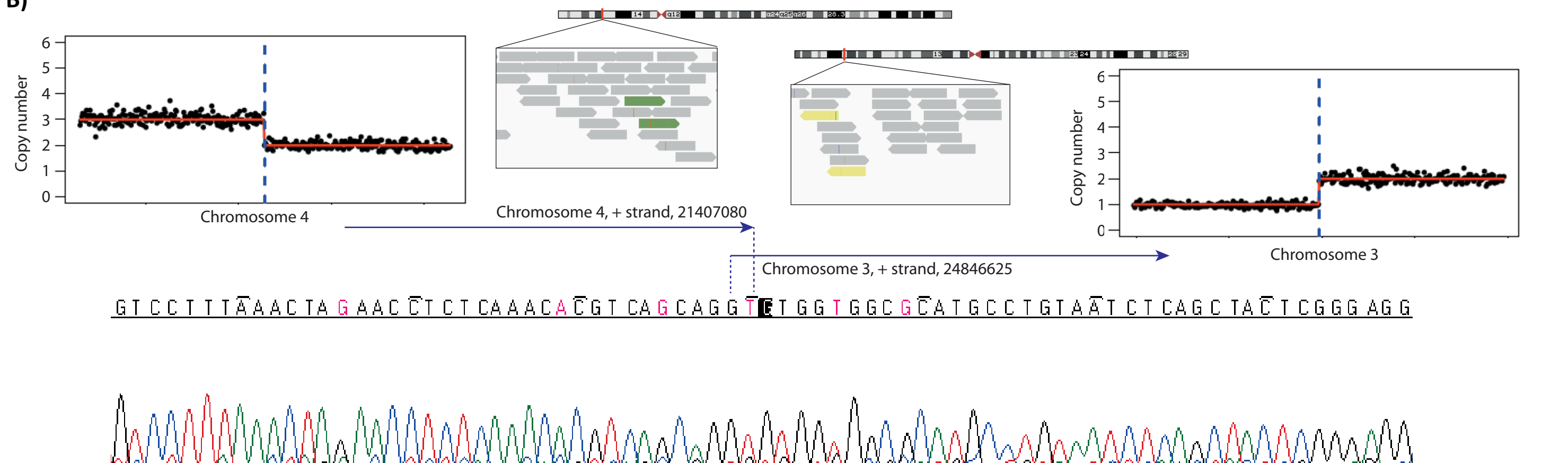

Supplement: Supplementary file 7 — Various types of somatic alteration identified in the tumours of patients 1 and 2, using whole-genome sequencing. (A) Circos plots: copy number profile in the inner circle (blue). Interchromosomal rearrangements were validated by Sanger sequencing (purple lines). Mutated genes are coloured by mutation type, indicated in the legend. (B) Validation of the chromosome 3–4 rearrangement in patient 1. The rearrangement was supported by discordant read pair mappings (in green and yellow) and by coincidence of both break points with a copy number break. This rearrangement was confirmed by Sanger sequencing. The absolute copy numbers are based on results from GLAD analysis [file path0233-0039-sd7.pdf]

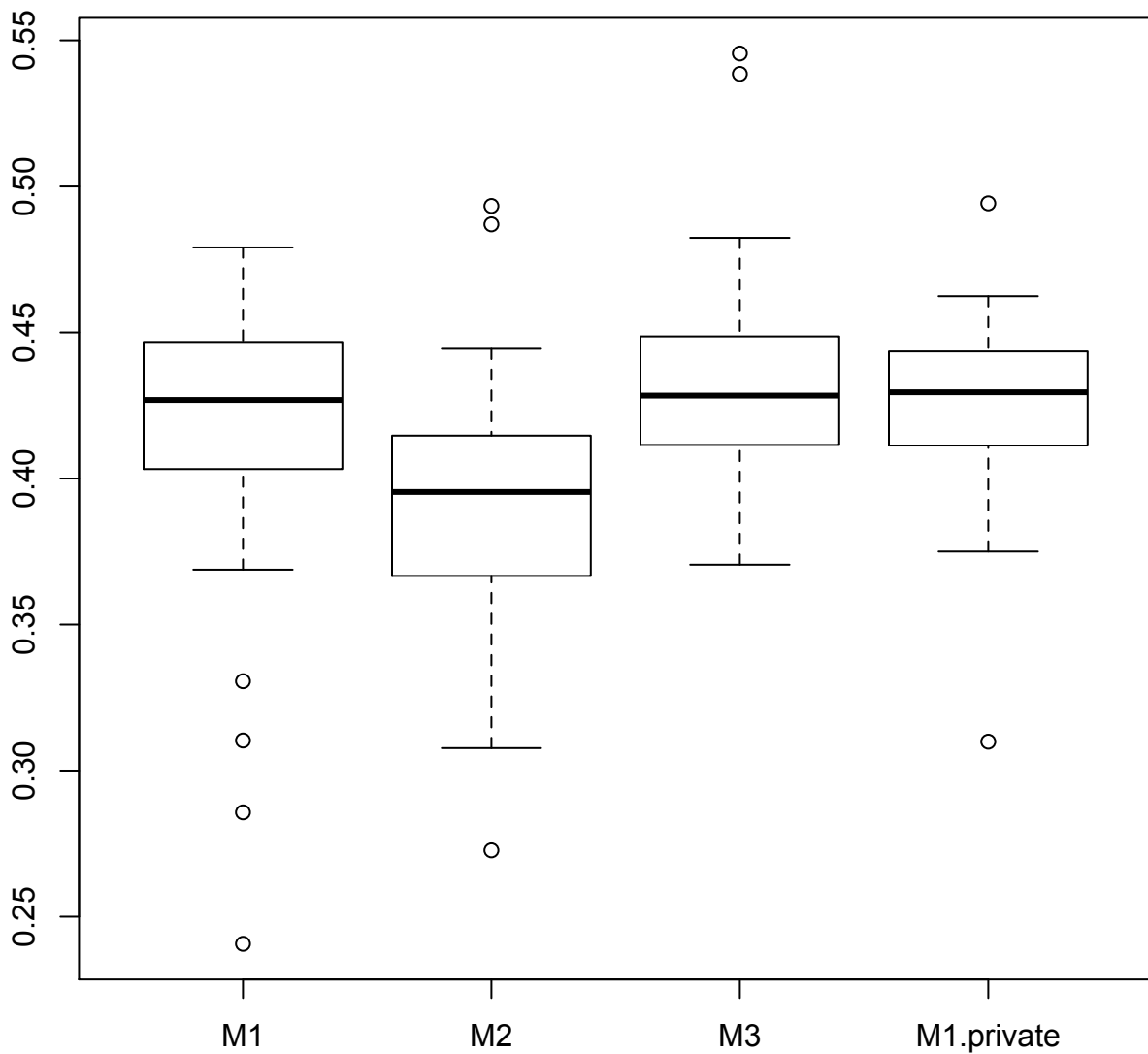

Supplement: Supplementary file 9 — Variant allele frequencies of the shared and M3 private mutations in patient 1. Only mutations in diploid regions were considered [file path0233-0039-sd9.pdf]
