# Supplementary material for: Molecular and genetic diversity in the metastatic process of melanoma
Source: J Pathol. 2014 Jan 27;233(1):39–50. doi: 10.1002/path.4318 (PMC4359751; doi:10.1002/path.4318)
Supplement: Supplementary file 11 — Sequencing data metrics [file path0233-0039-sd11.doc]

**Table S1.** Sequencing data metrics

|  |  |  | **Target capture sequencing metrics** | **Whole-genome sequencing metrics** | | |
| --- | --- | --- | --- | --- | --- | --- |
| **Patient ID** | **Sample** |  | **Mean target coverage** | **Median insert size, bp*** | **Sequence haploid coverage** | **Physical haploid coverage** |
| 1 | M1 |  | 350 | 407 | 3.8 | 11.5 |
| 1 | M2 |  | 277 | 419 | 2.9 | 8.9 |
| 1 | M3 |  | 497 | 369 | 4.4 | 12.4 |
| 1 | Normal |  | 272 | 549 | 2.0 | 7.3 |
| 2 | M1 |  | 316 | 493 | 3.0 | 10.2 |
| 2 | M2 |  | 310 | 392 | 4.2 | 12.4 |
| 2 | Normal |  | 294 | 517 | 3.6 | 12.8 |
| 3 | M1 |  | 247 |  |  |  |
| 3 | M2 |  | 198 |  |  |  |
| 3 | Normal |  | 205 |  |  |  |
| 4 | M1 |  | 316 |  |  |  |
| 4 | M2 |  | 305 |  |  |  |
| 4 | M3 |  | 338 |  |  |  |
| 4 | Normal |  | 302 |  |  |  |
| 5 | M1 |  | 594 |  |  |  |
| 5 | M2 |  | 322 |  |  |  |
| 5 | Normal |  | 272 |  |  |  |
| 6 | M1 |  | 457 |  |  |  |
| 6 | M2 |  | 434 |  |  |  |
| 6 | Normal |  | 264 |  |  |  |
| 7 | M1 |  | 490 |  |  |  |
| 7 | M2 |  | 320 |  |  |  |
| 7 | Normal |  | 307 |  |  |  |
| 8 | M1 |  | 200 |  |  |  |
| 8 | M2 |  | 518 |  |  |  |
| 8 | M3 |  | 426 |  |  |  |
| 8 | Normal |  | 268 |  |  |  |
| 10 | M2 |  | 402 |  |  |  |
| 10 | M3 |  | 546 |  |  |  |
| 10 | Normal |  | 380 |  |  |  |
| 11 | M1 |  | 567 |  |  |  |
| 11 | M2 |  | 319 |  |  |  |
| 11 | Normal |  | 330 |  |  |  |
| 12 | M1 |  | 546 |  |  |  |
| 12 | M2 |  | 390 |  |  |  |
| 12 | M3 |  | 370 |  |  |  |
| 12 | Normal |  | 301 |  |  |  |
| 14 | M1 |  | 354 |  |  |  |
| 14 | M2 |  | 331 |  |  |  |
| 14 | M3 |  | 371 |  |  |  |
| 14 | Normal |  | 328 |  |  |  |
| 15 | P |  | 352 |  |  |  |
| 15 | M1 |  | 307 |  |  |  |
| 15 | Normal |  | 306 |  |  |  |
| 16 | M1 |  | 423 |  |  |  |
| 16 | M2 |  | 307 |  |  |  |
| 16 | Normal |  | 337 |  |  |  |
| 18 | M2 |  | 400 |  |  |  |
| 18 | M3 |  | 362 |  |  |  |
| 18 | Normal |  | 354 |  |  |  |
| 19 | M1 |  | 332 |  |  |  |
| 19 | M2 |  | 227 |  |  |  |
| 19 | Normal |  | 209 |  |  |  |
| 20 | M1 |  | 325 |  |  |  |
| 20 | M2 |  | 389 |  |  |  |
| 20 | Normal |  | 383 |  |  |  |
| 22 | M1 |  | 338 |  |  |  |
| 22 | M2 |  | 302 |  |  |  |
| 22 | Normal |  | 326 |  |  |  |
| 25 | M1 |  | 209 |  |  |  |
| 25 | M2 |  | 328 |  |  |  |
| 25 | Normal |  | 209 |  |  |  |
| 26 | M1 |  | 296 |  |  |  |
| 26 | M2 |  | 275 |  |  |  |
| 26 | Normal |  | 401 |  |  |  |
| 27 | M1 |  | 362 |  |  |  |
| 27 | M2 |  | 254 |  |  |  |
| 27 | Normal |  | 359 |  |  |  |
| 28 | M1 |  | 370 |  |  |  |
| 28 | M2 |  | 399 |  |  |  |
| 28 | Normal |  | 343 |  |  |  |

*Based on alignment
