# Supplementary material for: Molecular and genetic diversity in the metastatic process of melanoma
Source: J Pathol. 2014 Jan 27;233(1):39–50. doi: 10.1002/path.4318 (PMC4359751; doi:10.1002/path.4318)
Supplement: Supplementary file 12 — Genes (n = 1697) in the target enrichment panel [file path0233-0039-sd12.doc]

**Table S2.** Genes (*n =* 1697) in the target enrichment panel

| *ABCA1* | *ABCA3* | *ABCA4* | *ABCB10* | *ABCB11* | *ABCB6* | *ABCB8* | *ABCD3* |
| --- | --- | --- | --- | --- | --- | --- | --- |
| *ABHD12B* | *ABL1* | *ABL2* | *ABLIM1* | *AC141586.1* | *ACADM* | *ACAP1* | *ACBD6* |
| *ACCN1* | *ACCS* | *ACLY* | *ACO2* | *ACPL2* | *ACSL5* | *ACTB* | *ACTL9* |
| *ACVR1B* | *ACVR2A* | *ACY1* | *ADAM10* | *ADAM12* | *ADAM17* | *ADAM29* | *ADAMTS15* |
| *ADAMTS18* | *ADAMTS19* | *ADAMTSL3* | *ADAR* | *ADAT3* | *ADHFE1* | *ADRA1A* | *AFF1* |
| *AFF4* | *AGAP1* | *AGAP2* | *AGBL5* | *AGER* | *AGK* | *AGPAT5* | *AHCY* |
| *AHRR* | *AHSA2* | *AKAP6* | *AKAP8* | *AKAP9* | *AKT1* | *AKT2* | *AKT3* |
| *ALDH1A1* | *ALDH1A3* | *ALK* | *ALPK3* | *ALS2CL* | *ALS2CR12* | *AMDHD2* | *AMFR* |
| *AMPD2* | *ANAPC5* | *ANK1* | *ANK2* | *ANKHD1* | *ANKK1* | *ANKRD29* | *ANKRD30A* |
| *ANKRD5* | *ANXA1* | *AP1M1* | *APC* | *APC2* | *APCS* | *APEX1* | *APEX2* |
| *APH1A* | *APH1B* | *APLNR* | *APOC4* | *APOL1* | *APPL1* | *AQP8* | *AR* |
| *ARAP3* | *AREG* | *ARFGAP3* | *ARFGEF2* | *ARFIP1* | *ARFRP1* | *ARHGAP11A* | *ARHGAP29* |
| *ARHGEF12* | *ARHGEF38* | *ARHGEF4* | *ARID1A* | *ARID1B* | *ARRB1* | *ARV1* | *ASB11* |
| *ASGR1* | *ASL* | *ASTN2* | *ATF2* | *ATG4C* | *ATM* | *ATP2A3* | *ATP2B1* |
| *ATP6V0B* | *ATP8B1* | *ATP8B2* | *ATR* | *ATRN* | *ATRX* | *AURKA* | *AURKB* |
| *AVPI1* | *AVPR2* | *AXIN1* | *AXL* | *B3GALNT2* | *BAI2* | *BAI3* | *BAP1* |
| *BARD1* | *BAT2* | *BAT3* | *BAZ1A* | *BAZ1B* | *BBS9* | *BCAR1* | *BCAT1* |
| *BCCIP* | *BCKDK* | *BCL11A* | *BCL2* | *BCL6* | *BCL9* | *BCORL1* | *BCR* |
| *BDKRB2* | *BEND7* | *BGN* | *BIRC2* | *BIRC6* | *BLM* | *BMI1* | *BMP1* |
| *BMP2* | *BMPR1A* | *BMPR1B* | *BMPR2* | *BOC* | *BRAF* | *BRAP* | *BRCA1* |
| *BRCA2* | *BRCC3* | *BRD4* | *BRIP1* | *BRMS1* | *BRSK1* | *BTG1* | *BTG2* |
| *BUB1* | *C10orf11* | *C10orf137* | *C11orf10* | *C11orf57* | *C12orf9* | *C14orf101* | *C14orf21* |
| *C15orf2* | *C15orf55* | *C16orf58* | *C17orf57* | *C17orf66* | *C18orf19* | *C19orf6* | *C1orf101* |
| *C1orf175* | *C1orf201* | *C1orf64* | *C1orf87* | *C1QB* | *C20orf103* | *C20orf177* | *C2orf44* |
| *C4orf14* | *C4orf50* | *C5orf34* | *C6orf145* | *C6orf204* | *C6orf97* | *C7orf11* | *CABLES1* |
| *CACNA1F* | *CAMK1G* | *CARD10* | *CASC5* | *CASP8* | *CAV1* | *CBFB* | *CBLB* |
| *CBX1* | *CBX2* | *CBX4* | *CBX7* | *CBX8* | *CCDC117* | *CCND1* | *CCND2* |
| *CCNE1* | *CCNE2* | *CCNF* | *CCNT1* | *CCR2* | *CCR3* | *CD109* | *CD2* |
| *CD22* | *CD24* | *CD248* | *CD44* | *CD46* | *CDC27* | *CDC42BPA* | *CDC42BPB* |
| *CDC6* | *CDC73* | *CDH1* | *CDH10* | *CDH12* | *CDH20* | *CDH3* | *CDH8* |
| *CDK1* | *CDK12* | *CDK15* | *CDK2* | *CDK2AP1* | *CDK4* | *CDKL2* | *CDKL3* |
| *CDKN1A* | *CDKN1B* | *CDKN2A* | *CDKN2B* | *CDKN2C* | *CDON* | *CDS1* | *CELA1* |
| *CELSR1* | *CENPE* | *CFH* | *CFHR5* | *CFL2* | *CFP* | *CHD3* | *CHD5* |
| *CHD8* | *CHEK1* | *CHEK2* | *CHL1* | *CHRND* | *CHUK* | *CIC* | *CIT* |
| *CLASRP* | *CLCA2* | *CLCN1* | *CLIP1* | *CLPTM1L* | *CLSPN* | *CLTC* | *CMKLR1* |
| *CNGA2* | *CNNM4* | *CNTN1* | *CNTN4* | *CNTN6* | *CNTNAP1* | *CNTROB* | *COG3* |
| *COL11A1* | *COL19A1* | *COL1A1* | *COL6A3* | *COL7A1* | *COX11* | *CPA3* | *CPS1* |
| *CPSF3* | *CREB3L2* | *CRX* | *CRYAA* | *CRYAB* | *CSF1* | *CSF1R* | *CSMD3* |
| *CSNK1A1* | *CSNK1D* | *CST4* | *CTAGE5* | *CTCF* | *CTNNA1* | *CTNNB1* | *CTNND1* |
| *CUBN* | *CUL9* | *CUTA* | *CUTC* | *CXCR5* | *CXorf30* | *CXorf48* | *CXorf57* |
| *CYB5R4* | *CYLD* | *CYP1A1* | *CYP26A1* | *CYP39A1* | *CYTH1* | *DAPK1* | *DAZAP1* |
| *DAZL* | *DBN1* | *DCC* | *DCLK3* | *DCT* | *DDIT3* | *DDO* | *DDR1* |
| *DDR2* | *DDX10* | *DDX18* | *DDX3X* | *DDX59* | *DEK* | *DGKB* | *DGKE* |
| *DGKG* | *DHH* | *DHX32* | *DIP2C* | *DKK3* | *DLC1* | *DLEC1* | *DLG1* |
| *DLG4* | *DLK1* | *DLK2* | *DLL1* | *DLL3* | *DLL4* | *DMBT1* | *DNAH5* |
| *DNAH9* | *DNAJA3* | *DNAJC12* | *DNAJC21* | *DNAJC24* | *DNASE1L3* | *DNER* | *DNMT1* |
| *DNMT3A* | *DNMT3B* | *DNMT3L* | *DOPEY1* | *DPAGT1* | *DPP10* | *DRG1* | *DSCR6* |
| *DTX1* | *DTX2* | *DTX3* | *DTX3L* | *DTX4* | *DUSP1* | *DUSP12* | *DUSP2* |
| *DVL3* | *DYRK1A* | *DYRK2* | *DYSF* | *E2F1* | *ECT2* | *EDN3* | *EDNRA* |
| *EDNRB* | *EED* | *EEF2K* | *EFTUD2* | *EGF* | *EGFL6* | *EGFR* | *EGR1* |
| *EGR3* | *EHBP1* | *EHF* | *EHMT1* | *EIF3K* | *EIF3M* | *EIF4A2* | *EIF5* |
| *ELF5* | *ELK1* | *ELK3* | *ELK4* | *ELP2* | *ENPEP* | *EOMES* | *EP300* |
| *EPCAM* | *EPHA1* | *EPHA10* | *EPHA3* | *EPHA6* | *EPHA8* | *EPHB1* | *EPHB3* |
| *EPHB4* | *EPHB6* | *ERAS* | *ERBB2* | *ERBB3* | *ERBB4* | *ERC1* | *ERCC3* |
| *ERCC6* | *EREG* | *ERG* | *ERGIC3* | *ERICH1* | *ERO1L* | *ERP27* | *ESR1* |
| *ESR2* | *ETS1* | *ETS2* | *ETV1* | *ETV2* | *ETV3* | *ETV4* | *ETV5* |
| *ETV6* | *ETV7* | *EVL* | *EWSR1* | *EXOC2* | *EXOC3L* | *EXOC4* | *EXT2* |
| *EYA4* | *EZH1* | *EZH2* | *FAAH* | *FABP4* | *FAM107B* | *FAM123B* | *FAM131A* |
| *FAM189B* | *FAM47B* | *FAM70A* | *FAM83F* | *FANCA* | *FANCB* | *FANCC* | *FANCD2* |
| *FANCE* | *FANCF* | *FANCG* | *FANCL* | *FANCM* | *FARP1* | *FAS* | *FASTKD3* |
| *FAT1* | *FBXL18* | *FBXL19* | *FBXO32* | *FBXO38* | *FBXO41* | *FBXO46* | *FBXO8* |
| *FBXW7* | *FCRL3* | *FCRL5* | *FEM1C* | *FERMT2* | *FEV* | *FGA* | *FGF10* |
| *FGF13* | *FGF19* | *FGF21* | *FGF23* | *FGF3* | *FGF4* | *FGF5* | *FGF6* |
| *FGF8* | *FGFR1* | *FGFR2* | *FGFR3* | *FGFR4* | *FHDC1* | *FHIT* | *FHOD1* |
| *FIGF* | *FKTN* | *FLCN* | *FLI1* | *FLNB* | *FLT1* | *FLT3* | *FLT4* |
| *FMNL3* | *FN1* | *FNDC3B* | *FOLR2* | *FOXA1* | *FOXA3* | *FOXC2* | *FOXD1* |
| *FOXD3* | *FOXO1* | *FOXO3* | *FOXO4* | *FOXP1* | *FOXP2* | *FOXP4* | *FPR3* |
| *FRMD6* | *FRMPD1* | *FRMPD3* | *FSCB* | *FUCA1* | *FURIN* | *FUS* | *FXR1* |
| *FZD10* | *G3BP2* | *G6PC* | *GAB1* | *GAB2* | *GABRA4* | *GABRP* | *GALNS* |
| *GALNT5* | *GATA3* | *GEN1* | *GFRAL* | *GGA1* | *GGA3* | *GIMAP1* | *GJB1* |
| *GKN1* | *GLI1* | *GLI2* | *GLP1R* | *GLT25D2* | *GMDS* | *GNA11* | *GNAO1* |
| *GNAQ* | *GNAS* | *GNB1L* | *GNPAT* | *GOLGA4* | *GOLGB1* | *GOLIM4* | *GP5* |
| *GPC1* | *GPC2* | *GPC3* | *GPNMB* | *GPR112* | *GPR115* | *GPR161* | *GPR180* |
| *GPR45* | *GPR81* | *GPR84* | *GPRC5B* | *GRB7* | *GREB1L* | *GRIK2* | *GRIK3* |
| *GRIN2A* | *GRIN2C* | *GRIN2D* | *GRIPAP1* | *GRK6* | *GRM1* | *GRM5* | *GRM6* |
| *GRM8* | *GSDMB* | *GSK3A* | *GSK3B* | *GSN* | *GSTCD* | *GTF2A1* | *GTF2H4* |
| *GUCY1A2* | *GUCY2F* | *HADHB* | *HAPLN1* | *HAUS3* | *HCN3* | *HCRTR2* | *HDAC1* |
| *HDAC10* | *HDAC11* | *HDAC2* | *HDAC3* | *HDAC4* | *HDAC5* | *HDAC6* | *HDAC7* |
| *HDAC8* | *HDAC9* | *HDLBP* | *HECW1* | *HELQ* | *HEPACAM2* | *HERC1* | *HERC4* |
| *HES1* | *HES2* | *HES3* | *HES4* | *HES5* | *HES6* | *HES7* | *HEY1* |
| *HEY2* | *HEYL* | *HIC1* | *HIF1A* | *HIP1* | *HIST1H1B* | *HIST1H4L* | *HIST2H2AB* |
| *HK3* | *HLCS* | *HM13* | *HMGA2* | *HMGN1* | *HMGXB3* | *HN1* | *HNF1A* |
| *HNF4A* | *HOOK1* | *HOOK3* | *HOXA11* | *HOXA3* | *HOXA4* | *HOXC13* | *HRAS* |
| *HSD11B1* | *HSD17B8* | *HSP90AA1* | *HSP90AB1* | *HSPA14* | *HSPA4* | *HTATSF1* | *HUWE1* |
| *ICAM5* | *IFNA2* | *IFNB1* | *IGF1R* | *IGF2* | *IHH* | *IKBKAP* | *IKBKB* |
| *IKZF1* | *IL1B* | *IL1R2* | *IL1RAPL2* | *IL21R* | *IL6R* | *IL7R* | *INA* |
| *INHBE* | *INPP5K* | *INPPL1* | *INSR* | *INTS4* | *IRAK1* | *IRAK3* | *IRAK4* |
| *IRF8* | *IRS1* | *IRS2* | *IRS4* | *ITCH* | *ITGA6* | *ITGA9* | *ITGB1* |
| *ITGB2* | *ITGB3* | *ITGB7* | *ITIH5L* | *ITK* | *ITPR1* | *ITPR2* | *ITPR3* |
| *JAG1* | *JAG2* | *JAK1* | *JAK2* | *JAK3* | *JARID2* | *JAZF1* | *JMJD1C* |
| *JUP* | *KALRN* | *KBTBD8* | *KCNA5* | *KCNC2* | *KCNIP3* | *KCNJ1* | *KCNJ15* |
| *KCNQ5* | *KCNT2* | *KDM3A* | *KDM5D* | *KDM6A* | *KDM6B* | *KEAP1* | *KIAA0182* |
| *KIAA0427* | *KIAA1012* | *KIAA1324* | *KIAA1409* | *KIAA1468* | *KIAA1632* | *KIF16B* | *KIF18B* |
| *KIF1C* | *KIF27* | *KIF6* | *KIT* | *KITLG* | *KLF6* | *KLHL4* | *KLK15* |
| *KPNA5* | *KRAS* | *KRT73* | *KRT76* | *KRTAP10-8* | *KRTAP20-1* | *KRTAP21-1* | *KTN1* |
| *LAMA2* | *LAMB1* | *LATS1* | *LATS2* | *LDHB* | *LDHC* | *LEF1* | *LEPREL1* |
| *LFNG* | *LGALS2* | *LGR6* | *LHCGR* | *LHFP* | *LIPE* | *LLGL1* | *LMF2* |
| *LMO7* | *LNPEP* | *LONRF3* | *LOXL2* | *LOXL4* | *LPAR2* | *LPHN2* | *LPHN3* |
| *LPP* | *LRBA* | *LRP1* | *LRP2* | *LRP5* | *LRRC4* | *LRRC41* | *LRRC7* |
| *LRRFIP1* | *LSP1* | *LYN* | *LZTS2* | *MACF1* | *MAF* | *MAG* | *MAGEA1* |
| *MAGEA4* | *MAGEB16* | *MAGEC2* | *MAGED2* | *MAGEE1* | *MAGI1* | *MAGI2* | *MAGOHB* |
| *MAK* | *MAMDC4* | *MAML1* | *MAML2* | *MAML3* | *MANEA* | *MAOA* | *MAP2* |
| *MAP2K4* | *MAP3K1* | *MAP3K11* | *MAP3K12* | *MAP3K14* | *MAP3K6* | *MAP3K8* | *MAP7D2* |
| *MAPK10* | *MAPK11* | *MAPK13* | *MAPK8IP3* | *MAPKAPK3* | *MAS1L* | *MAST2* | *MAST4* |
| *MAX* | *MBD1* | *MBD2* | *MBD4* | *MBD6* | *MBOAT2* | *MC3R* | *MCART1* |
| *MCF2L2* | *MCHR2* | *MCOLN1* | *MCPH1* | *MDC1* | *MDM2* | *MED1* | *MED14* |
| *MEF2C* | *MEN1* | *MET* | *MEX3B* | *MFAP2* | *MFAP5* | *MFNG* | *MFSD9* |
| *MGC42105* | *MIA2* | *MIB1* | *MIB2* | *MICAL1* | *MICALL1* | *MITF* | *MKL1* |
| *MKNK2* | *MKRN3* | *MLF1* | *MLH1* | *MLH3* | *MLL* | *MLL2* | *MLL3* |
| *MLLT6* | *MLST8* | *MMP10* | *MMP11* | *MMP14* | *MMP15* | *MMP2* | *MMP24* |
| *MMP26* | *MMP27* | *MMP28* | *MMP8* | *MN1* | *MORC1* | *MOSPD1* | *MRE11A* |
| *MSH2* | *MSH3* | *MSH5* | *MSH6* | *MSI1* | *MSI2* | *MTL5* | *MTMR3* |
| *MTMR8* | *MTOR* | *MUC1* | *MUC16* | *MUTYH* | *MXD1* | *MXI1* | *MYB* |
| *MYC* | *MYCL1* | *MYCN* | *MYD88* | *MYEOV* | *MYH1* | *MYH11* | *MYH8* |
| *MYH9* | *MYLK4* | *MYO3A* | *MYOD1* | *MYST4* | *N4BP2* | *NAA25* | *NALCN* |
| *NANOG* | *NBN* | *NCOA2* | *NCOA6* | *NCOR2* | *NCSTN* | *NDRG2* | *NDUFA2* |
| *NDUFA3* | *NDUFA8* | *NEDD4* | *NEFM* | *NEK2* | *NET1* | *NEURL* | *NF1* |
| *NF2* | *NFIA* | *NFIL3* | *NFKB1* | *NFKBIA* | *NFYC* | *NHS* | *NID2* |
| *NIP7* | *NIPBL* | *NKAP* | *NKD2* | *NLE1* | *NLRC5* | *NLRP1* | *NLRP14* |
| *NLRP7* | *NLRP8* | *NLRP9* | *NMBR* | *NMUR2* | *NOP58* | *NOS2* | *NOTCH1* |
| *NOTCH2* | *NOTCH3* | *NOTCH4* | *NOVA1* | *NPBWR1* | *NPM1* | *NPR1* | *NR2F2* |
| *NR4A3* | *NRAS* | *NRCAM* | *NRG1* | *NRG2* | *NRG3* | *NRK* | *NRXN3* |
| *NSMCE2* | *NTN1* | *NTRK1* | *NTRK3* | *NUAK2* | *NUDCD1* | *NUMB* | *NUMBL* |
| *NUP133* | *NUP153* | *NUP214* | *NUP98* | *NXN* | *OBSCN* | *OCA2* | *ODZ1* |
| *OLA1* | *OR12D3* | *OR1N1* | *ORAOV1* | *OSBPL11* | *OSTC* | *OTC* | *OTOF* |
| *OTUD4* | *OTUD7B* | *OVCA2* | *OXER1* | *OXSM* | *P2RX7* | *P2RY10* | *P2RY14* |
| *PACS1* | *PADI3* | *PALB2* | *PANX2* | *PARP1* | *PARP10* | *PARP14* | *PARP2* |
| *PATZ1* | *PAX3* | *PAX7* | *PAX8* | *PAXIP1* | *PBRM1* | *PCDH19* | *PCDH20* |
| *PCDH7* | *PCDH8* | *PCDH9* | *PCDHB15* | *PCGF1* | *PCGF2* | *PCGF3* | *PCGF5* |
| *PCGF6* | *PCM1* | *PCSK5* | *PDCD4* | *PDCD6* | *PDCD6,AHRR* | *PDE2A* | *PDE4A* |
| *PDE4D* | *PDE8B* | *PDGFB* | *PDGFRA* | *PDGFRB* | *PDIA4* | *PDLIM7* | *PDPK1* |
| *PDZD3* | *PDZD4* | *PER1* | *PER2* | *PFKFB4* | *PGBD3* | *PGR* | *PHC1* |
| *PHC2* | *PHC3* | *PHF7* | *PHIP* | *PHKB* | *PHOX2B* | *PIAS2* | *PIGN* |
| *PIGS* | *PIK3C2A* | *PIK3C2B* | *PIK3C2G* | *PIK3C3* | *PIK3CA* | *PIK3CB* | *PIK3CD* |
| *PIK3CG* | *PIK3R1* | *PIK3R2* | *PIK3R3* | *PIK3R4* | *PIK3R5* | *PKD1L1* | *PKDCC* |
| *PKDREJ* | *PKHD1* | *PKM2* | *PKN1* | *PKNOX1* | *PLA2G4A* | *PLA2R1* | *PLAG1* |
| *PLAT* | *PLCB1* | *PLCB2* | *PLCD3* | *PLCG1* | *PLD2* | *PLEKHA8* | *PLOD1* |
| *PLS3* | *PLXNB1* | *PLXND1* | *PML* | *PMS1* | *PMS2* | *PNCK* | *PNLIPRP1* |
| *POFUT1* | *POLB* | *POLH* | *POLQ* | *POLR2F* | *POP1* | *POU2F1* | *POU3F2* |
| *POU5F1* | *PPA1* | *PPAPDC3* | *PPARG* | *PPARGC1B* | *PPFIBP2* | *PPHLN1* | *PPM1E* |
| *PPM1F* | *PPM1J* | *PPM1L* | *PPP1CA* | *PPP1R12A* | *PPP1R3A* | *PPP1R3C* | *PPP2R3A* |
| *PPP2R5C* | *PRCC* | *PRDM16* | *PRDM4* | *PRDX5* | *PREX2* | *PRICKLE3* | *PRKAA1* |
| *PRKAA2* | *PRKAR1A* | *PRKAR2A* | *PRKCA* | *PRKCB* | *PRKCE* | *PRKCI* | *PRKD1* |
| *PRKDC* | *PRODH* | *PROM1* | *PROS1* | *PRPF4B* | *PRPS1* | *PRPSAP1* | *PRRG1* |
| *PRRT1* | *PSD* | *PSEN1* | *PSEN2* | *PSENEN* | *PSIP1* | *PSMD14* | *PSME4* |
| *PTCH1* | *PTCH2* | *PTCHD2* | *PTEN* | *PTGFR* | *PTGIS* | *PTH2R* | *PTHLH* |
| *PTP4A1* | *PTP4A2* | *PTP4A3* | *PTPDC1* | *PTPLA* | *PTPLAD1* | *PTPLAD2* | *PTPLB* |
| *PTPMT1* | *PTPN1* | *PTPN11* | *PTPN12* | *PTPN13* | *PTPN14* | *PTPN18* | *PTPN2* |
| *PTPN20A* | *PTPN20A,PTPN20B* | *PTPN20B* | *PTPN21* | *PTPN22* | *PTPN23* | *PTPN3* | *PTPN4* |
| *PTPN5* | *PTPN6* | *PTPN7* | *PTPN9* | *PTPRA* | *PTPRB* | *PTPRC* | *PTPRD* |
| *PTPRE* | *PTPRF* | *PTPRG* | *PTPRH* | *PTPRJ* | *PTPRK* | *PTPRM* | *PTPRN* |
| *PTPRN2* | *PTPRO* | *PTPRQ* | *PTPRR* | *PTPRS* | *PTPRT* | *PTPRU* | *PTPRZ1* |
| *PTRF* | *PUS1* | *RAB27A* | *RAB38* | *RAB41* | *RAC2* | *RAD50* | *RAD51* |
| *RAD51C* | *RAD51L1* | *RAD51L3* | *RAF1* | *RAGE* | *RAI2* | *RALA* | *RALB* |
| *RANBP1* | *RAP1GAP* | *RAP1GDS1* | *RAPH1* | *RARA* | *RARB* | *RARG* | *RASA2* |
| *RASAL2* | *RASEF* | *RASGRF2* | *RASL10B* | *RASL11A* | *RASSF1* | *RASSF2* | *RB1* |
| *RBBP4* | *RBBP7* | *RBM10* | *RBM14* | *RBM15* | *RBMS1* | *RBPJ* | *RBX1* |
| *RCE1* | *RECK* | *RECQL4* | *REL* | *REPS1* | *RET* | *RFC4* | *RFNG* |
| *RFWD3* | *RFX2* | *RG9MTD2* | *RGL1* | *RGL2* | *RGS22* | *RHAG* | *RHD* |
| *RHEB* | *RHEBL1* | *RHOBTB2* | *RICTOR* | *RIF1* | *RIMS1* | *RIN1* | *RING1* |
| *RNASEH2A* | *RNF113A* | *RNF149* | *RNF152* | *RNF2* | *RNF20* | *RNF213* | *RNF216* |
| *RNF220* | *ROCK1* | *ROD1* | *ROR1* | *ROR2* | *ROS1* | *RP1-32I10.10* | *RPL3* |
| *RPRD1A* | *RPS6KA1* | *RPS6KA2* | *RPS6KA3* | *RPS6KA4* | *RPS6KA5* | *RPS6KB1* | *RPS6KB2* |
| *RPS6KC1* | *RPS9* | *RPTOR* | *RPUSD4* | *RRAS2* | *RREB1* | *RRP15* | *RRP9* |
| *RSPO2* | *RSPO3* | *RTP1* | *RUFY1* | *RUNX1* | *RUNX1T1* | *RYK* | *SATL1* |
| *SAV1* | *SBNO1* | *SCARA3* | *SCARF2* | *SCEL* | *SCGB3A1* | *SCGB3A2* | *SCML1* |
| *SCML2* | *SCN3B* | *SCNN1B* | *SCP2* | *SDHB* | *SEMA3A* | *SEMA4G* | *SEMA5B* |
| *SEMA7A* | *SENP5* | *SENP6* | *SEPT8* | *SEPT9* | *SERGEF* | *SERPINB1* | *SERPINE2* |
| *SETD2* | *SF1* | *SFMBT2* | *SGK1* | *SGK2* | *SGK3* | *SGK494* | *SH2B3* |
| *SH2D3A* | *SH3RF1* | *SHCBP1* | *SHFM1* | *SHROOM2* | *SIGLEC1* | *SIK3* | *SIP1* |
| *SIPA1L1* | *SIRPB2* | *SIX4* | *SKIV2L* | *SLAMF1* | *SLC12A3* | *SLC16A2* | *SLC17A6* |
| *SLC22A1* | *SLC22A2* | *SLC22A9* | *SLC24A4* | *SLC26A6* | *SLC29A1* | *SLC35A2* | *SLC39A12* |
| *SLC44A4* | *SLC45A2* | *SLC4A3* | *SLC4A7* | *SLC6A3* | *SLC6A5* | *SLC7A7* | *SLC8A3* |
| *SLC9A2* | *SLC9A3R1* | *SLC9A3R2* | *SLC9A5* | *SLCO2B1* | *SMAD2* | *SMAD3* | *SMAD4* |
| *SMARCA4* | *SMARCAD1* | *SMARCAL1* | *SMARCB1* | *SMC1A* | *SMC2* | *SMC4* | *SMC6* |
| *SMG1* | *SMO* | *SMOX* | *SMYD3* | *SNAI1* | *SNAI2* | *SNAI3* | *SNRK* |
| *SNX21* | *SNX25* | *SNX4* | *SOCS1* | *SOCS2* | *SORBS1* | *SORCS1* | *SORL1* |
| *SOS1* | *SOS2* | *SOX10* | *SOX7* | *SOX9* | *SP1* | *SP110* | *SPAG6* |
| *SPAG9* | *SPAST* | *SPATA21* | *SPEN* | *SPI1* | *SPO11* | *SPOCD1* | *SPTAN1* |
| *SPTLC1* | *SQLE* | *SREBF2* | *SRF* | *SRGAP3* | *SRSF1* | *SRSF6* | *SSFA2* |
| *SSH2* | *SSNA1* | *STARD8* | *STAT1* | *STAT3* | *STAT4* | *STAT5A* | *STAT5B* |
| *STK11* | *STK32B* | *STK32C* | *STK36* | *STMN1* | *STRADB* | *STRBP* | *STX12* |
| *STX5* | *SUFU* | *SULF2* | *SURF1* | *SUSD1* | *SUSD3* | *SUV39H2* | *SUZ12* |
| *SYCP1* | *SYNE1* | *SYNE2* | *SYT3* | *TACC2* | *TACR3* | *TAF1* | *TAF15* |
| *TAF1L* | *TAF7L* | *TAS2R13* | *TAX1BP1* | *TBC1D19* | *TBC1D9B* | *TBCEL* | *TBK1* |
| *TBX1* | *TBX2* | *TBX22* | *TBX5* | *TBXAS1* | *TCF12* | *TCF3* | *TCF7L1* |
| *TCF7L2* | *TCP1* | *TEC* | *TECTA* | *TEK* | *TEKT4* | *TESK1* | *TESK2* |
| *TET1* | *TEX14* | *TFAP2D* | *TFE3* | *TFF1* | *TFG* | *TGFB1* | *TGFB3* |
| *TGFBR2* | *TGS1* | *THBS3* | *THOC5* | *THRSP* | *TIAM1* | *TICAM1* | *TIMELESS* |
| *TIPARP* | *TLE1* | *TLL1* | *TLN1* | *TLR10* | *TLR2* | *TLR4* | *TLR7* |
| *TMCC1* | *TMED1* | *TMEM125* | *TMEM161A* | *TMEM206* | *TMEM39A* | *TMPRSS15* | *TMPRSS2* |
| *TMPRSS3* | *TMPRSS6* | *TMTC4* | *TNFRSF11A* | *TNFRSF25* | *TNS1* | *TOP1* | *TOP2B* |
| *TOPBP1* | *TOR1AIP1* | *TOX3* | *TP53* | *TP63* | *TP73* | *TPM3* | *TPM4* |
| *TPTE* | *TRAF2* | *TRAF3* | *TRAF5* | *TRAF7* | *TREM1* | *TREML1* | *TRIM24* |
| *TRIM25* | *TRIM28* | *TRIM29* | *TRIM33* | *TRIM36* | *TRIM37* | *TRIM42* | *TRIM47* |
| *TRIM67* | *TRIML1* | *TRIO* | *TRIP11* | *TRMT2A* | *TRPC4* | *TRPM1* | *TRPM7* |
| *TSC1* | *TSC2* | *TSC22D4* | *TSN* | *TSPAN4* | *TTC15* | *TTC3* | *TTLL3* |
| *TTN* | *TTPAL* | *TUSC2* | *TXNDC15* | *TXNDC3* | *TYK2* | *TYR* | *TYRP1* |
| *UBE2I* | *UBE2O* | *UBE4A* | *UBR4* | *UBR5* | *UGT1A9* | *UHRF2* | *ULK3* |
| *UNC45B* | *UQCC* | *UQCR11* | *UQCRC2* | *USH1G* | *USP16* | *USP18* | *USP19* |
| *USP20* | *USP21* | *USP24* | *USP28* | *USP29* | *USP31* | *USP32* | *USP34* |
| *USP36* | *USP40* | *USP42* | *USP45* | *USP50* | *USP51* | *USP53* | *USP54* |
| *USP6* | *USP9X* | *UTP20* | *UTS2R* | *VAV1* | *VAV2* | *VAV3* | *VCPIP1* |
| *VEPH1* | *VHL* | *VPS13B* | *VPS72* | *VWF* | *WARS* | *WBP4* | *WDR53* |
| *WDR59* | *WDR69* | *WDR88* | *WEE1* | *WFDC1* | *WHSC1L1* | *WNK1* | *WNK2* |
| *WNT1* | *WNT2* | *WNT2B* | *WNT3* | *WNT3A* | *WNT5A* | *WNT7A* | *WNT8B* |
| *WT1* | *WWC1* | *WWP2* | *XBP1* | *XDH* | *XIRP1* | *XKRX* | *XPA* |
| *XPC* | *XRCC1* | *XRCC2* | *YSK4* | *YWHAQ* | *YY2* | *ZBTB16* | *ZBTB3* |
| *ZCCHC14* | *ZCCHC24* | *ZDHHC4* | *ZFP64* | *ZFYVE26* | *ZIC3* | *ZMIZ1* | *ZNF10* |
| *ZNF217* | *ZNF22* | *ZNF25* | *ZNF277* | *ZNF281* | *ZNF318* | *ZNF350* | *ZNF365* |
| *ZNF384* | *ZNF432* | *ZNF436* | *ZNF438* | *ZNF442* | *ZNF532* | *ZNF546* | *ZNF569* |
| *ZNF598* | *ZNF644* | *ZNF646* | *ZNF668* | *ZNF695* | *ZNF703* | *ZNF746* | *ZNF75A* |
| *ZNHIT2* |  |  |  |  |  |  |  |
