# Supplementary material for: Molecular and genetic diversity in the metastatic process of melanoma
Source: J Pathol. 2014 Jan 27;233(1):39–50. doi: 10.1002/path.4318 (PMC4359751; doi:10.1002/path.4318)
Supplement: Supplementary file 13 — Regions of differential copy number between metastases in patient 1 [file path0233-0039-sd13.doc]

**Table S3.** Regions of differential copy number between metastases in patient 1

| **Chrom** | **Start** | **End** | **Band1** | **Band2** | **M1** | **M2** | **M3** |
| --- | --- | --- | --- | --- | --- | --- | --- |
| 1 | 145150001 | 209250001 | q21.1 | q32.2 | 3 | 4 | 4 |
| 1 | 237850001 | 249250001 | q43 | q44 | 3 | 4 | 4 |
| 2 | 1 | 13450001 | p25.3 | p24.3 | 2 | 1 | 1 |
| 3 | 1 | 24800001 | p26.3 | p24.2 | 1(0*) | 2(1*) | 2(1*) |
| 3 | 114700001 | 115550001 | q13.31 | q13.31 | 2 | 3 | 2 |
| 4 | 100001 | 21350001 | p16.3 | p15.2 | 3 | 2 | 2 |
| 4 | 112450001 | 113050001 | q25 | q25 | 2 | 1 | 1 |
| 6 | 56100001 | 57150001 | p12.1 | p11.2 | 3 | 4 | 4 |
| 6 | 57550001 | 58050001 | p11.2 | p11.2 | 2 | 3 | 3 |
| 6 | 58150001 | 58700001 | p11.2 | p11.1 | 3 | 4 | 4 |
| 6 | 58800001 | 94500001 | p11.1 | q16.1 | 2 | 3 | 3 |
| 6 | 134400001 | 135450001 | q23.2 | q23.3 | 1 | 0 | 0 |
| 9 | 50001 | 26050001 | p24.3 | p21.2 | 1 | 2 | 2 |
| 13 | 54200001 | 72550001 | q14.3 | q21.33 | 3 | 4 | 4 |
| 13 | 73650001 | 80550001 | q22.1 | q31.1 | 3 | 4 | 4 |
| 13 | 81350001 | 112300001 | q31.1 | q34 | 3 | 4 | 4 |
| 16 | 76000001 | 76600001 | q23.1 | q23.1 | 2 | 1 | 1 |
| 17 | 400001 | 7550001 | p13.3 | p13.1 | 2 | 1 | 1 |
| 21 | 11150001 | 22800001 | p11.1 | q21.1 | 3 | 2 | 2 |
| 21 | 22850001 | 43500001 | q21.1 | q22.3 | 4 | 2 | 2 |
| 21 | 43550001 | 48100001 | q22.3 | q22.3 | 3 | 2 | 2 |
| X | 88850001 | 89400001 | q21.31 | q21.31 | 3 | 2 | 2 |
| X | 138450001 | 139550001 | q27.1 | q27.1 | 1 | 2 | 2 |
| X | 145700001 | 146250001 | q27.3 | q27.3 | 1 | 2 | 2 |

*Small deletion in a larger deleted segment.
