# Supplementary material for: Molecular and genetic diversity in the metastatic process of melanoma
Source: J Pathol. 2014 Jan 27;233(1):39–50. doi: 10.1002/path.4318 (PMC4359751; doi:10.1002/path.4318)
Supplement: Supplementary file 5 — DNA copy number changes in melanoma-relevant genes across the patients. Nine genes were selected, based on previous studies showing frequent alterations in melanoma tumours [file path0233-0039-sd5.pdf]

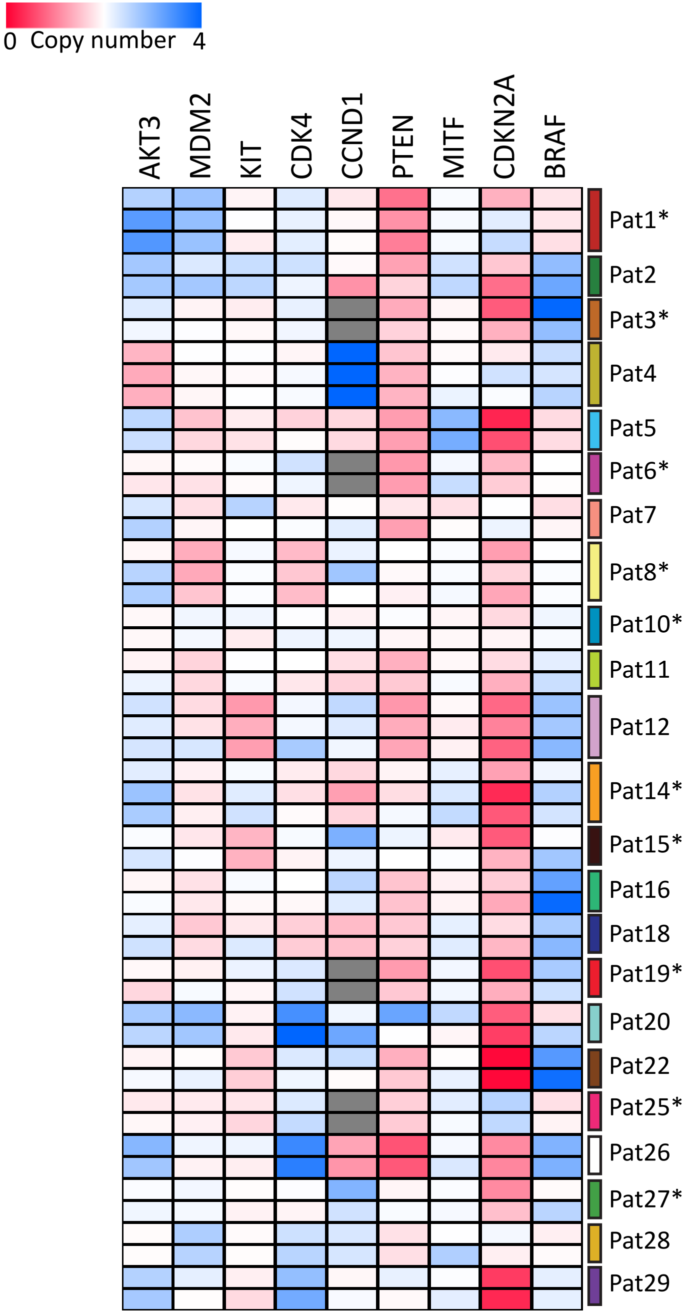
0 Copy number 4
AKT3
MDM2
KIT
CDK4
CCND1
PTEN
MITF
CDKN2A
BRAF
Pat1*
Pat2
Pat3*
Pat4
Pat5
Pat6*
Pat7
Pat8*
Pat10*
Pat11
Pat12
Pat14*
Pat15*
Pat16
Pat18
Pat19*
Pat20
Pat22
Pat25*
Pat26
Pat27*
Pat28
Pat29
