# Supplementary material for: Molecular and genetic diversity in the metastatic process of melanoma
Source: J Pathol. 2014 Jan 27;233(1):39–50. doi: 10.1002/path.4318 (PMC4359751; doi:10.1002/path.4318)
Supplement: Supplementary file 14 — Coding sequence (exonic and splice site) mutations in patients 1–28 [file path0233-0039-sd14.zip › TableS10.doc]

**Table S10.** Exonic and splice site mutations in patient 7

| **chrom** | **position** | **reference** | **alternative** | **M1** | **M2** | **Gene** | **Type** |
| --- | --- | --- | --- | --- | --- | --- | --- |
| 1 | 115256527 | CTT | TTC | 1 | 1 | *NRAS* | non-frameshift substitution |
| 1 | 178442227 | C | T | 1 | 1 | *RASAL2* | synonymous SNV |
| 2 | 152320180 | G | A | 1 | 1 | *RIF1* | synonymous SNV |
| 2 | 179459119 | C | T | 1 | 1 | *TTN* | non-synonymous SNV |
| 2 | 179639748 | G | C | 1 | 1 | *TTN* | non-synonymous SNV |
| 3 | 39228429 | G | A | 1 | 1 | *XIRP1* | synonymous SNV |
| 3 | 194117952 | GG | AA | 1 | 1 | *GP5* | non-frameshift substitution |
| 4 | 166996153 | A | T | 1 | 1 | *TLL1* | non-synonymous SNV |
| 6 | 31133754 | GT | AA | 1 | 1 | *POU5F1* | non-frameshift substitution |
| 6 | 70064186 | C | T | 1 | 1 | *BAI3* | non-synonymous SNV |
| 6 | 100395841 | C | T | 1 | 1 | *MCHR2* | synonymous SNV |
| 6 | 109769564 | A | G | 1 | 1 | *MICAL1* | non-synonymous SNV |
| 6 | 160670377 | C | T | 1 | 1 | *SLC22A2* | non-synonymous SNV |
| 7 | 6179783 | C | T | 1 | 1 | *USP42* | synonymous SNV |
| 7 | 157985078 | C | T | 1 | 1 | *PTPRN2* | non-synonymous SNV |
| 8 | 100205115 | C | T | 1 | 1 | *VPS13B* | non-synonymous SNV |
| 9 | 37707421 | T | C | 1 | 1 | *FRMPD1* | non-synonymous SNV |
| 9 | 78601108 | C | T | 1 | 1 | *PCSK5* | non-synonymous SNV |
| 9 | 85615951 | C | T | 1 | 1 | *RASEF* | non-synonymous SNV |
| 9 | 94871063 | G | A | 1 | 1 | *SPTLC1* | synonymous SNV |
| 9 | 112151549 | G | A | 1 | 1 | *PTPN3* | synonymous SNV |
| 10 | 114903762 | C | T | 1 | 1 | *TCF7L2* | non-synonymous SNV |
| 11 | 48185143 | CC | TT | 1 | 1 | *PTPRJ* | non-frameshift substitution |
| 13 | 110435182 | G | A | 1 | 1 | *IRS2* | synonymous SNV |
| 14 | 101351001 | C | T | 1 | 1 | *RTL1* | non-synonymous SNV |
| 15 | 56142773 | G | T | 1 | 1 | *NEDD4* | non-synonymous SNV |
| 15 | 64067545 | A | G | 1 | 1 | *HERC1* | non-synonymous SNV |
| 16 | 23391786 | G | A | 1 | 1 | *SCNN1B* | stopgain SNV |
| 16 | 47536957 | T | G | 1 | 1 | *PHKB* | non-synonymous SNV |
| 16 | 52479954 | T | C | 1 | 1 | *TOX3* | synonymous SNV |
| 16 | 52553371 | C | T | 1 | 1 | *TOX3* | non-synonymous SNV |
| 18 | 59221485 | G | A | 1 | 1 | *CDH20* | non-synonymous SNV |
| 19 | 9058659 | G | A | 1 | 1 | *MUC16* | non-synonymous SNV |
| 19 | 11136158 | C | T | 1 | 1 | *SMARCA4* | non-synonymous SNV |
| 19 | 56477621 | C | T | 1 | 1 | *NLRP8* | synonymous SNV |
| 20 | 48127700 | C | T | 1 | 1 | *PTGIS* | non-synonymous SNV |
| 20 | 50769672 | G | A | 1 | 1 | *ZFP64* | synonymous SNV |
| 21 | 32638732 | G | A | 1 | 1 | *TIAM1* | non-synonymous SNV |
| X | 41204501 | TC | AT | 1 | 1 | *DDX3X* | non-frameshift substitution |
| X | 41205577 | A | T | 1 | 1 | *DDX3X* | non-synonymous SNV |
| X | 107978341 | C | T | 1 | 1 | *IRS4* | non-synonymous SNV |
| 9 | 130453057 | C | T | 1 | 1 | *STXBP1* | non-synonymous SNV |
| 12 | 57922169 | C | T | 0 | 1 | *MBD6* | synonymous SNV |
| 18 | 8376139 | C | T | 0 | 1 | *PTPRM* | synonymous SNV |
| 22 | 18900866 | C | - | 0 | 1 | *PRODH* | frameshift deletion |
| X | 41075506 | C | T | 0 | 1 | *USP9X* | non-synonymous SNV |
| X | 47485543 | A | C | 0 | 1 | *CFP* | non-synonymous SNV |
| X | 76875892 | C | T | 0 | 1 | *ATRX* | non-synonymous SNV |
