# Supplementary material for: Molecular and genetic diversity in the metastatic process of melanoma
Source: J Pathol. 2014 Jan 27;233(1):39–50. doi: 10.1002/path.4318 (PMC4359751; doi:10.1002/path.4318)
Supplement: Supplementary file 14 — Coding sequence (exonic and splice site) mutations in patients 1–28 [file path0233-0039-sd14.zip › TableS11.doc]

**Table S11.** Exonic and splice site mutations in patient 8

| **Chrom** | **Position** | **Reference** | **Alternative** | **M1** | **M2** | **M3** | **Gene** | **Type** |
| --- | --- | --- | --- | --- | --- | --- | --- | --- |
| 1 | 11561369 | C | T | 1 | 1 | 1 | *PTCHD2* | non-synonymous SNV |
| 1 | 115256530 | G | T | 1 | 1 | 1 | *NRAS* | non-synonymous SNV |
| 1 | 228553174 | G | A | 1 | 1 | 1 | *OBSCN* | non-synonymous SNV |
| 2 | 179426689 | C | T | 1 | 1 | 1 | *TTN* | non-synonymous SNV |
| 2 | 179457386 | T | C | 1 | 1 | 1 | *TTN* | synonymous SNV |
| 2 | 179591984 | C | T | 1 | 1 | 1 | *TTN* | non-synonymous SNV |
| 3 | 52454955 | C | T | 1 | 1 | 1 | *PHF7* | synonymous SNV |
| 3 | 58196568 | C | T | 1 | 1 | 1 | *DNASE1L3* | synonymous SNV |
| 3 | 65364966 | C | T | 1 | 1 | 1 | *MAGI1* | non-synonymous SNV |
| 3 | 96963065 | G | A | 1 | 1 | 1 | *EPHA6* | non-synonymous SNV |
| 3 | 178916904 | C | T | 1 | 1 | 1 | *PIK3CA* | synonymous SNV |
| 3 | 196612385 | C | T | 1 | 1 | 1 | *SENP5* | synonymous SNV |
| 6 | 72889347 | G | A | 1 | 1 | 1 | *RIMS1* | non-synonymous SNV |
| 6 | 79692622 | G | A | 1 | 1 | 1 | *PHIP* | non-synonymous SNV |
| 7 | 139661795 | C | T | 1 | 1 | 1 | *TBXAS1* | synonymous SNV |
| 10 | 114901043 | C | T | 1 | 1 | 1 | *TCF7L2* | non-synonymous SNV |
| 11 | 34502397 | G | A | 1 | 1 | 1 | *ELF5* | non-synonymous SNV |
| 11 | 72316250 | T | G | 1 | 1 | 1 | *PDE2A* | synonymous SNV |
| 11 | 77775090 | G | A | 1 | 1 | 1 | *THRSP* | non-synonymous SNV |
| 12 | 124817762 | G | A | 1 | 1 | 1 | *NCOR2* | synonymous SNV |
| 13 | 28886168 | C | T | 1 | 1 | 1 | *FLT1* | non-synonymous SNV |
| 13 | 49027196 | C | T | 1 | 1 | 1 | *RB1* | non-synonymous SNV |
| 14 | 94079310 | G | A | 1 | 1 | 1 | *UNC79* | non-synonymous SNV |
| 16 | 67662412 | C | T | 1 | 1 | 1 | *CTCF* | non-synonymous SNV |
| 17 | 3831970 | C | T | 1 | 1 | 1 | *ATP2A3* | synonymous SNV |
| 17 | 5462138 | G | A | 1 | 1 | 1 | *NLRP1* | synonymous SNV |
| 17 | 10322334 | C | T | 1 | 1 | 1 | *MYH8* | non-synonymous SNV |
| 17 | 10395759 | G | A | 1 | 1 | 1 | *MYH1* | non-synonymous SNV |
| 19 | 9062087 | C | T | 1 | 1 | 1 | *MUC16* | synonymous SNV |
| 19 | 9066390 | G | A | 1 | 1 | 1 | *MUC16* | non-synonymous SNV |
| 19 | 9066700 | G | A | 1 | 1 | 1 | *MUC16* | non-synonymous SNV |
| 19 | 41744394 | G | A | 1 | 1 | 1 | *AXL* | synonymous SNV |
| 19 | 57641061 | G | A | 1 | 1 | 1 | *USP29* | non-synonymous SNV |
| 20 | 57429264 | C | T | 1 | 1 | 1 | *GNAS* | non-synonymous SNV |
| 21 | 38269426 | G | A | 1 | 1 | 1 | *HLCS* | synonymous SNV |
| 22 | 28196402 | G | C | 1 | 1 | 1 | *MN1* | non-synonymous SNV |
| X | 105152992 | G | A | 1 | 1 | 1 | *NRK* | synonymous SNV |
| 16 | 57054875 | T | C | 0 | 1 | 0 | *NLRC5* | non-synonymous SNV |
| 1 | 103412477 | A | T | 0 | 0 | 1 | *COL11A1* | synonymous SNV |
| 1 | 103488500 | T | G | 0 | 0 | 1 | *COL11A1* | non-synonymous SNV |
| 19 | 57641738 | C | T | 0 | 0 | 1 | *USP29* | synonymous SNV |
| 20 | 10632878 | G | A | 0 | 0 | 1 | *JAG1* | non-synonymous SNV |
