# Supplementary material for: Molecular and genetic diversity in the metastatic process of melanoma
Source: J Pathol. 2014 Jan 27;233(1):39–50. doi: 10.1002/path.4318 (PMC4359751; doi:10.1002/path.4318)
Supplement: Supplementary file 14 — Coding sequence (exonic and splice site) mutations in patients 1–28 [file path0233-0039-sd14.zip › TableS12.doc]

**Table S12.** Exonic and splice site mutations in patient 10

| **Chrom** | **Position** | **Reference** | **Alternative** | **M2** | **M3** | **Gene** | **Type** |
| --- | --- | --- | --- | --- | --- | --- | --- |
| 1 | 16257643 | C | T | 1 | 1 | *SPEN* | synonymous SNV |
| 1 | 16261102 | C | T | 1 | 1 | *SPEN* | synonymous SNV |
| 1 | 19439072 | C | G | 1 | 1 | *UBR4* | non-synonymous SNV |
| 1 | 22924313 | A | G | 1 | 1 | *EPHA8* | non-synonymous SNV |
| 1 | 26885330 | C | T | 1 | 1 | *RPS6KA1* | non-synonymous SNV |
| 1 | 46876082 | C | T | 1 | 1 | *FAAH* | synonymous SNV |
| 1 | 94643612 | G | A | 1 | 1 | *ARHGAP29* | synonymous SNV |
| 1 | 115523998 | G | A | 1 | 1 | *SYCP1* | stopgain SNV |
| 1 | 153661567 | C | T | 1 | 1 | *NPR1* | synonymous SNV |
| 1 | 162745609 | C | T | 1 | 1 | *DDR2* | non-synonymous SNV |
| 2 | 17846847 | G | A | 1 | 1 | *SMC6* | synonymous SNV |
| 2 | 17888515 | G | A | 1 | 1 | *SMC6* | synonymous SNV |
| 2 | 29455216 | C | T | 1 | 1 | *ALK* | synonymous SNV |
| 2 | 42281400 | C | T | 1 | 1 | *PKDCC* | synonymous SNV |
| 2 | 48925816 | G | A | 1 | 1 | *LHCGR* | synonymous SNV |
| 2 | 48952886 | C | T | 1 | 1 | *LHCGR* | synonymous SNV |
| 2 | 71894601 | G | A | 1 | 1 | *DYSF* | non-synonymous SNV |
| 2 | 74734788 | G | A | 1 | 1 | *PCGF1* | non-synonymous SNV |
| 2 | 179506996 | G | A | 1 | 1 | *TTN* | non-synonymous SNV |
| 2 | 179549711 | C | T | 1 | 1 | *TTN* | non-synonymous SNV |
| 2 | 202342509 | G | A | 1 | 1 | *STRADB* | non-synonymous SNV |
| 2 | 212495235 | C | T | 1 | 1 | *ERBB4* | synonymous SNV |
| 2 | 230272035 | G | A | 1 | 1 | *DNER* | non-synonymous SNV |
| 2 | 239990267 | CG | AA | 1 | 1 | *HDAC4* | non-frameshift substitution |
| 3 | 431079 | G | A | 1 | 1 | *CHL1* | non-synonymous SNV |
| 3 | 10119825 | C | T | 1 | 1 | *FANCD2* | stopgain SNV |
| 3 | 12447572 | G | A | 1 | 1 | *PPARG* | non-synonymous SNV |
| 3 | 12627290 | G | A | 1 | 1 | *RAF1* | non-synonymous SNV |
| 3 | 39229526 | C | T | 1 | 1 | *XIRP1* | non-synonymous SNV |
| 3 | 47452277 | CC | TT | 1 | 1 | *PTPN23* | non-frameshift substitution |
| 3 | 71161725 | TT | AC | 1 | 1 | *FOXP1* | immediate-stopgain |
| 3 | 112969664 | C | T | 1 | 1 | *BOC* | synonymous SNV |
| 3 | 140401756 | C | T | 1 | 1 | *TRIM42* | non-synonymous SNV |
| 3 | 141291470 | A | T | 1 | 1 | *RASA2* | stopgain SNV |
| 3 | 141305507 | C | T | 1 | 1 | *RASA2* | stopgain SNV |
| 3 | 185906153 | C | G | 1 | 1 | *DGKG* | non-synonymous SNV |
| 5 | 13735343 | C | T | 1 | 1 | *DNAH5* | synonymous SNV |
| 5 | 13735969 | G | A | 1 | 1 | *DNAH5* | non-synonymous SNV |
| 5 | 38960633 | A | G | 1 | 1 | *RICTOR* | non-synonymous SNV |
| 5 | 66460538 | C | T | 1 | 1 | *MAST4* | non-synonymous SNV |
| 5 | 76645328 | G | A | 1 | 1 | *PDE8B* | non-synonymous SNV |
| 5 | 80037310 | G | A | 1 | 1 | *MSH3* | non-synonymous SNV |
| 5 | 82937369 | G | A | 1 | 1 | *HAPLN1* | synonymous SNV |
| 5 | 149499682 | A | G | 1 | 1 | *PDGFRB* | non-synonymous SNV |
| 5 | 167850577 | C | T | 1 | 1 | *WWC1* | synonymous SNV |
| 5 | 170239060 | C | T | 1 | 1 | *GABRP* | non-synonymous SNV |
| 5 | 180048755 | C | T | 1 | 1 | *FLT4* | non-synonymous SNV |
| 6 | 31609654 | G | A | 1 | 1 | *BAG6* | non-synonymous SNV |
| 6 | 36106520 | GG | AA | 1 | 1 | *MAPK13* | non-frameshift substitution |
| 6 | 36106781 | C | T | 1 | 1 | *MAPK13* | non-synonymous SNV |
| 6 | 51921696 | C | T | 1 | 1 | *PKHD1* | non-synonymous SNV |
| 6 | 72806823 | C | T | 1 | 1 | *RIMS1* | synonymous SNV |
| 6 | 99958056 | G | A | 1 | 1 | *USP45* | non-synonymous SNV |
| 6 | 117708063 | C | T | 1 | 1 | *ROS1* | non-synonymous SNV |
| 6 | 129649451 | G | A | 1 | 1 | *LAMA2* | non-synonymous SNV |
| 6 | 142397175 | C | T | 1 | 1 | *NMBR* | synonymous SNV |
| 6 | 146720405 | C | T | 1 | 1 | *GRM1* | non-synonymous SNV |
| 6 | 152631087 | G | A | 1 | 1 | *SYNE1* | synonymous SNV |
| 6 | 152631099 | G | A | 1 | 1 | *SYNE1* | synonymous SNV |
| 7 | 77998505 | G | A | 1 | 1 | *MAGI2* | synonymous SNV |
| 7 | 100414917 | C | T | 1 | 1 | *EPHB4* | synonymous SNV |
| 8 | 12957266 | G | A | 1 | 1 | *DLC1* | synonymous SNV |
| 8 | 100990262 | G | A | 1 | 1 | *RGS22* | synonymous SNV |
| 8 | 113349921 | C | T | 1 | 1 | *CSMD3* | non-synonymous SNV |
| 9 | 8404557 | A | G | 1 | 1 | *PTPRD* | non-synonymous SNV |
| 9 | 27197402 | G | A | 1 | 1 | *TEK* | non-synonymous SNV |
| 9 | 37744588 | C | T | 1 | 1 | *FRMPD1* | synonymous SNV |
| 9 | 78803532 | G | A | 1 | 1 | *PCSK5* | non-synonymous SNV |
| 9 | 90219935 | C | T | 1 | 1 | *DAPK1* | synonymous SNV |
| 9 | 96051268 | C | T | 1 | 1 | *WNK2* | non-synonymous SNV |
| 9 | 125289540 | G | A | 1 | 1 | *OR1N1* | synonymous SNV |
| 10 | 3824211 | C | T | 1 | 1 | *KLF6* | non-synonymous SNV |
| 10 | 16975233 | A | G | 1 | 1 | *CUBN* | non-synonymous SNV |
| 10 | 108371779 | CC | TT | 1 | 1 | *SORCS1* | non-frameshift substitution |
| 10 | 123845682 | C | T | 1 | 1 | *TACC2* | synonymous SNV |
| 10 | 127412498 | C | G | 1 | 1 | *C10orf137* | non-synonymous SNV |
| 10 | 127967521 | C | T | 1 | 1 | *ADAM12* | non-synonymous SNV |
| 11 | 44219492 | C | T | 1 | 1 | *EXT2* | synonymous SNV |
| 11 | 113935091 | C | T | 1 | 1 | *ZBTB16* | non-synonymous SNV |
| 11 | 118344036 | G | A | 1 | 1 | *MLL* | non-synonymous SNV |
| 11 | 121424650 | G | A | 1 | 1 | *SORL1* | synonymous SNV |
| 11 | 128638109 | G | A | 1 | 1 | *FLI1* | synonymous SNV |
| 12 | 56487639 | CC | TT | 1 | 1 | *ERBB3* | immediate-stopgain |
| 12 | 70933765 | G | A | 1 | 1 | *PTPRB* | non-synonymous SNV |
| 12 | 80943390 | G | A | 1 | 1 | *PTPRQ* | non-synonymous SNV |
| 12 | 108686417 | C | T | 1 | 1 | *CMKLR1* | non-synonymous SNV |
| 12 | 124915240 | C | T | 1 | 1 | *NCOR2* | non-synonymous SNV |
| 13 | 48923136 | G | A | 1 | 1 | *RB1* | stopgain SNV |
| 15 | 84611433 | G | A | 1 | 1 | *ADAMTSL3* | non-synonymous SNV |
| 16 | 1810507 | C | T | 1 | 1 | *MAPK8IP3* | synonymous SNV |
| 16 | 9857222 | G | A | 1 | 1 | *GRIN2A* | non-synonymous SNV |
| 16 | 9858306 | G | A | 1 | 1 | *GRIN2A* | non-synonymous SNV |
| 16 | 25228627 | G | A | 1 | 1 | *AQP8* | non-synonymous SNV |
| 17 | 7577106 | G | A | 1 | 1 | *TP53* | non-synonymous SNV |
| 17 | 7579474 | G | A | 1 | 1 | *TP53* | synonymous SNV |
| 17 | 10398383 | C | T | 1 | 1 | *MYH1* | synonymous SNV |
| 17 | 11607645 | C | T | 1 | 1 | *DNAH9* | synonymous SNV |
| 17 | 29563040 | G | A | 1 | 1 | *NF1* | splicing |
| 17 | 29665157 | GG | AA | 1 | 1 | *NF1* | frameshift substitution |
| 17 | 40035095 | C | T | 1 | 1 | *ACLY* | non-synonymous SNV |
| 17 | 42169109 | C | T | 1 | 1 | *HDAC5* | non-synonymous SNV |
| 17 | 45438748 | C | T | 1 | 1 | *EFCAB13* | synonymous SNV |
| 18 | 50278496 | G | A | 1 | 1 | *DCC* | non-synonymous SNV |
| 19 | 6832135 | GG | AA | 1 | 1 | *VAV1* | non-frameshift substitution |
| 19 | 56243490 | G | A | 1 | 1 | *NLRP9* | synonymous SNV |
| 20 | 10036253 | T | A | 1 | 1 | *ANKRD5* | non-synonymous SNV |
| 20 | 40790091 | G | A | 1 | 1 | *PTPRT* | synonymous SNV |
| 20 | 43057009 | C | T | 1 | 1 | *HNF4A* | synonymous SNV |
| 20 | 46313181 | C | T | 1 | 1 | *SULF2* | non-synonymous SNV |
| 21 | 38538012 | G | T | 1 | 1 | *TTC3* | non-synonymous SNV |
| X | 17819753 | C | T | 1 | 1 | *RAI2* | synonymous SNV |
| X | 84363957 | C | T | 1 | 1 | *SATL1* | synonymous SNV |
| 14 | 94046618 | G | A | 1 | 0 | *UNC79* | non-synonymous SNV |
| 19 | 9047173 | C | T | 1 | 0 | *MUC16* | synonymous SNV |
| 19 | 9068102 | T | A | 1 | 0 | *MUC16* | non-synonymous SNV |
| 19 | 40762906 | G | A | 1 | 0 | *AKT2* | synonymous SNV |
