# Supplementary material for: Molecular and genetic diversity in the metastatic process of melanoma
Source: J Pathol. 2014 Jan 27;233(1):39–50. doi: 10.1002/path.4318 (PMC4359751; doi:10.1002/path.4318)
Supplement: Supplementary file 14 — Coding sequence (exonic and splice site) mutations in patients 1–28 [file path0233-0039-sd14.zip › TableS13.doc]

**Table S13.** Exonic and splice site mutations in patient 11

| **Chrom** | **Position** | **Reference** | **Alternative** | **M1** | **M2** | **Gene** | **Type** |
| --- | --- | --- | --- | --- | --- | --- | --- |
| 1 | 16260079 | C | T | 1 | 1 | *SPEN* | synonymous SNV |
| 1 | 33797992 | G | A | 1 | 1 | *PHC2* | non-synonymous SNV |
| 1 | 38227533 | C | T | 1 | 1 | *EPHA10* | non-synonymous SNV |
| 1 | 45288217 | G | A | 1 | 1 | *PTCH2* | non-synonymous SNV |
| 1 | 46871374 | C | T | 1 | 1 | *FAAH* | synonymous SNV |
| 1 | 57173348 | G | A | 1 | 1 | *PRKAA2* | non-synonymous SNV |
| 1 | 64643262 | A | G | 1 | 1 | *ROR1* | non-synonymous SNV |
| 1 | 70504941 | G | A | 1 | 1 | *LRRC7* | non-synonymous SNV |
| 1 | 70555491 | CC | TT | 1 | 1 | *LRRC7* | non-frameshift substitution |
| 1 | 103343611 | G | A | 1 | 1 | *COL11A1* | synonymous SNV |
| 1 | 114968316 | GA | AC | 1 | 1 | *TRIM33* | non-frameshift substitution |
| 1 | 149915798 | C | T | 1 | 1 | *OTUD7B* | synonymous SNV |
| 1 | 154315696 | C | T | 1 | 1 | *ATP8B2* | non-synonymous SNV |
| 1 | 157504507 | G | T | 1 | 1 | *FCRL5* | synonymous SNV |
| 1 | 186901908 | C | T | 1 | 1 | *PLA2G4A* | non-synonymous SNV |
| 2 | 3391812 | C | T | 1 | 1 | *TRAPPC12* | non-synonymous SNV |
| 2 | 16082336 | G | A | 1 | 1 | *MYCN* | stopgain SNV |
| 2 | 17962283 | C | T | 1 | 1 | *GEN1* | stopgain SNV |
| 2 | 26698231 | G | A | 1 | 1 | *OTOF* | non-synonymous SNV |
| 2 | 31625933 | G | A | 1 | 1 | *XDH* | non-synonymous SNV |
| 2 | 73491484 | G | A | 1 | 1 | *FBXO41* | synonymous SNV |
| 2 | 121747144 | G | A | 1 | 1 | *GLI2* | synonymous SNV |
| 2 | 170009392 | G | A | 1 | 1 | *LRP2* | synonymous SNV |
| 2 | 170044661 | G | A | 1 | 1 | *LRP2* | synonymous SNV |
| 2 | 170063040 | G | A | 1 | 1 | *LRP2* | non-synonymous SNV |
| 2 | 179400522 | G | A | 1 | 1 | *TTN* | non-synonymous SNV |
| 2 | 179416439 | T | G | 1 | 1 | *TTN* | non-synonymous SNV |
| 2 | 179438081 | C | T | 1 | 1 | *TTN* | non-synonymous SNV |
| 2 | 179462648 | C | T | 1 | 1 | *TTN* | non-synonymous SNV |
| 2 | 179496952 | C | T | 1 | 1 | *TTN* | non-synonymous SNV |
| 2 | 179497405 | C | T | 1 | 1 | *TTN* | non-synonymous SNV |
| 2 | 179548006 | C | T | 1 | 1 | *TTN* | non-synonymous SNV |
| 2 | 179577963 | G | A | 1 | 1 | *TTN* | synonymous SNV |
| 2 | 179579896 | G | A | 1 | 1 | *TTN* | non-synonymous SNV |
| 2 | 179587148 | C | T | 1 | 1 | *TTN* | non-synonymous SNV |
| 2 | 179588610 | C | T | 1 | 1 | *TTN* | non-synonymous SNV |
| 2 | 179588831 | T | C | 1 | 1 | *TTN* | non-synonymous SNV |
| 2 | 179595687 | C | T | 1 | 1 | *TTN* | non-synonymous SNV |
| 2 | 179596985 | G | A | 1 | 1 | *TTN* | non-synonymous SNV |
| 2 | 179612627 | G | A | 1 | 1 | *TTN* | non-synonymous SNV |
| 2 | 179632851 | C | T | 1 | 1 | *TTN* | synonymous SNV |
| 2 | 179639849 | C | T | 1 | 1 | *TTN* | non-synonymous SNV |
| 2 | 210559172 | C | T | 1 | 1 | *MAP2* | non-synonymous SNV |
| 2 | 212248565 | CT | TA | 1 | 1 | *ERBB4* | non-frameshift substitution |
| 2 | 212568876 | G | A | 1 | 1 | *ERBB4* | synonymous SNV |
| 2 | 230377541 | C | T | 1 | 1 | *DNER* | non-synonymous SNV |
| 2 | 238275927 | T | C | 1 | 1 | *COL6A3* | non-synonymous SNV |
| 2 | 238283143 | G | A | 1 | 1 | *COL6A3* | synonymous SNV |
| 2 | 242169586 | G | A | 1 | 1 | *HDLBP* | synonymous SNV |
| 3 | 3076407 | C | T | 1 | 1 | *CNTN4* | synonymous SNV |
| 3 | 37090087 | G | A | 1 | 1 | *MLH1* | non-synonymous SNV |
| 3 | 38139002 | C | T | 1 | 1 | *DLEC1* | synonymous SNV |
| 3 | 39230003 | C | T | 1 | 1 | *XIRP1* | non-synonymous SNV |
| 3 | 43344815 | A | G | 1 | 1 | *SNRK* | synonymous SNV |
| 3 | 46399978 | C | T | 1 | 1 | *CCR2* | synonymous SNV |
| 3 | 58139285 | C | T | 1 | 1 | *FLNB* | non-synonymous SNV |
| 3 | 89259117 | C | T | 1 | 1 | *EPHA3* | synonymous SNV |
| 3 | 89521616 | C | T | 1 | 1 | *EPHA3* | non-synonymous SNV |
| 3 | 97439174 | G | A | 1 | 1 | *EPHA6* | non-synonymous SNV |
| 3 | 121409630 | G | A | 1 | 1 | *GOLGB1* | stopgain SNV |
| 3 | 121438558 | G | A | 1 | 1 | *GOLGB1* | non-synonymous SNV |
| 3 | 130452957 | G | A | 1 | 1 | *PIK3R4* | synonymous SNV |
| 3 | 169993022 | C | T | 1 | 1 | *PRKCI* | non-synonymous SNV |
| 3 | 186015898 | C | T | 1 | 1 | *DGKG* | non-synonymous SNV |
| 3 | 189582089 | G | A | 1 | 1 | *TP63* | non-synonymous SNV |
| 3 | 189690756 | C | T | 1 | 1 | *LEPREL1* | non-synonymous SNV |
| 4 | 1801150 | G | A | 1 | 1 | *FGFR3* | synonymous SNV |
| 4 | 41749426 | G | A | 1 | 1 | *PHOX2B* | synonymous SNV |
| 4 | 104579541 | G | T | 1 | 1 | *TACR3* | non-synonymous SNV |
| 4 | 111398197 | G | A | 1 | 1 | *ENPEP* | synonymous SNV |
| 4 | 166795177 | G | A | 1 | 1 | *TLL1* | non-synonymous SNV |
| 4 | 187628307 | G | A | 1 | 1 | *FAT1* | non-synonymous SNV |
| 4 | 189061693 | G | A | 1 | 1 | *TRIML1* | synonymous SNV |
| 5 | 1036467 | G | A | 1 | 1 | *NKD2* | non-synonymous SNV |
| 5 | 13737382 | C | T | 1 | 1 | *DNAH5* | non-synonymous SNV |
| 5 | 13786369 | G | A | 1 | 1 | *DNAH5* | synonymous SNV |
| 5 | 24505352 | G | A | 1 | 1 | *CDH10* | non-synonymous SNV |
| 5 | 24511555 | C | T | 1 | 1 | *CDH10* | non-synonymous SNV |
| 5 | 44388629 | A | T | 1 | 1 | *FGF10* | synonymous SNV |
| 5 | 96315551 | C | T | 1 | 1 | *LNPEP* | synonymous SNV |
| 5 | 137803311 | A | T | 1 | 1 | *EGR1* | synonymous SNV |
| 5 | 149502718 | C | T | 1 | 1 | *PDGFRB* | synonymous SNV |
| 5 | 151784000 | G | A | 1 | 1 | *NMUR2* | synonymous SNV |
| 5 | 167824802 | C | T | 1 | 1 | *WWC1* | synonymous SNV |
| 5 | 178418481 | G | A | 1 | 1 | *GRM6* | synonymous SNV |
| 5 | 180043463 | C | T | 1 | 1 | *FLT4* | synonymous SNV |
| 5 | 180043921 | C | T | 1 | 1 | *FLT4* | non-synonymous SNV |
| 6 | 32180929 | G | A | 1 | 1 | *NOTCH4* | synonymous SNV |
| 6 | 32181925 | G | A | 1 | 1 | *NOTCH4* | non-synonymous SNV |
| 6 | 32187928 | C | T | 1 | 1 | *NOTCH4* | synonymous SNV |
| 6 | 36339303 | CC | TT | 1 | 1 | *ETV7* | non-frameshift substitution |
| 6 | 70589539 | G | A | 1 | 1 | *COL19A1* | non-synonymous SNV |
| 6 | 72967859 | A | G | 1 | 1 | *RIMS1* | synonymous SNV |
| 6 | 73904811 | G | A | 1 | 1 | *KCNQ5* | non-synonymous SNV |
| 6 | 100390994 | G | A | 1 | 1 | *MCHR2* | stopgain SNV |
| 6 | 128326314 | A | T | 1 | 1 | *PTPRK* | non-synonymous SNV |
| 6 | 142409653 | G | A | 1 | 1 | *NMBR* | non-synonymous SNV |
| 6 | 152652811 | G | A | 1 | 1 | *SYNE1* | stopgain SNV |
| 6 | 152826379 | G | A | 1 | 1 | *SYNE1* | synonymous SNV |
| 7 | 14724946 | G | A | 1 | 1 | *DGKB* | synonymous SNV |
| 7 | 37907403 | G | A | 1 | 1 | *NME8* | non-synonymous SNV |
| 7 | 47840380 | G | A | 1 | 1 | *PKD1L1* | non-synonymous SNV |
| 7 | 47942107 | C | T | 1 | 1 | *PKD1L1* | non-synonymous SNV |
| 7 | 55220249 | C | T | 1 | 1 | *EGFR* | synonymous SNV |
| 7 | 92848731 | G | A | 1 | 1 | *HEPACAM2* | non-synonymous SNV |
| 7 | 113518733 | G | A | 1 | 1 | *PPP1R3A* | non-synonymous SNV |
| 7 | 126173776 | C | T | 1 | 1 | *GRM8* | non-synonymous SNV |
| 7 | 140453136 | AC | TT | 1 | 1 | *BRAF* | non-frameshift substitution |
| 8 | 12957905 | G | A | 1 | 1 | *DLC1* | synonymous SNV |
| 8 | 23217622 | G | A | 1 | 1 | *LOXL2* | non-synonymous SNV |
| 8 | 24774740 | G | A | 1 | 1 | *NEFM* | non-synonymous SNV |
| 8 | 24775564 | G | A | 1 | 1 | *NEFM* | synonymous SNV |
| 8 | 27516635 | C | T | 1 | 1 | *SCARA3* | synonymous SNV |
| 8 | 32616925 | C | T | 1 | 1 | *NRG1* | synonymous SNV |
| 8 | 48809758 | G | A | 1 | 1 | *PRKDC* | unknown |
| 8 | 68965371 | G | A | 1 | 1 | *PREX2* | stopgain SNV |
| 8 | 68968149 | G | A | 1 | 1 | *PREX2* | non-synonymous SNV |
| 8 | 68984751 | G | A | 1 | 1 | *PREX2* | synonymous SNV |
| 8 | 69009418 | C | T | 1 | 1 | *PREX2* | synonymous SNV |
| 8 | 69129915 | C | T | 1 | 1 | *PREX2* | non-synonymous SNV |
| 8 | 71068364 | G | A | 1 | 1 | *NCOA2* | non-synonymous SNV |
| 8 | 93088252 | C | T | 1 | 1 | *RUNX1T1* | non-synonymous SNV |
| 8 | 113256661 | G | A | 1 | 1 | *CSMD3* | non-synonymous SNV |
| 9 | 21971091 | G | A | 1 | 1 | *CDKN2A* | non-synonymous SNV |
| 9 | 21971111 | G | A | 1 | 1 | *CDKN2A* | non-synonymous SNV |
| 9 | 119770383 | C | T | 1 | 1 | *ASTN2* | non-synonymous SNV |
| 10 | 3823858 | C | T | 1 | 1 | *KLF6* | synonymous SNV |
| 10 | 18254504 | C | T | 1 | 1 | *SLC39A12* | synonymous SNV |
| 10 | 18266993 | C | T | 1 | 1 | *SLC39A12* | non-synonymous SNV |
| 10 | 26377312 | T | A | 1 | 1 | *MYO3A* | non-synonymous SNV |
| 10 | 26385584 | T | C | 1 | 1 | *MYO3A* | synonymous SNV |
| 10 | 84744962 | G | A | 1 | 1 | *NRG3* | synonymous SNV |
| 10 | 123845990 | C | T | 1 | 1 | *TACC2* | synonymous SNV |
| 10 | 124345695 | G | A | 1 | 1 | *DMBT1* | non-synonymous SNV |
| 11 | 3744626 | G | A | 1 | 1 | *NUP98* | non-synonymous SNV |
| 11 | 77667011 | G | A | 1 | 1 | *INTS4* | non-synonymous SNV |
| 11 | 108564248 | C | T | 1 | 1 | *DDX10* | non-synonymous SNV |
| 11 | 113264332 | C | T | 1 | 1 | *ANKK1* | synonymous SNV |
| 12 | 6138563 | G | A | 1 | 1 | *VWF* | non-synonymous SNV |
| 12 | 41316117 | G | A | 1 | 1 | *CNTN1* | non-synonymous SNV |
| 12 | 49446807 | G | A | 1 | 1 | *MLL2* | non-synonymous SNV |
| 12 | 54756927 | G | A | 1 | 1 | *GPR84* | non-synonymous SNV |
| 12 | 54757174 | G | A | 1 | 1 | *GPR84* | synonymous SNV |
| 12 | 56363324 | G | A | 1 | 1 | *CDK2* | synonymous SNV |
| 12 | 56827351 | CC | TT | 1 | 1 | *TIMELESS* | non-frameshift substitution |
| 12 | 64890745 | C | T | 1 | 1 | *TBK1* | non-synonymous SNV |
| 12 | 71016196 | G | A | 1 | 1 | *PTPRB* | stopgain SNV |
| 12 | 75444312 | C | T | 1 | 1 | *KCNC2* | synonymous SNV |
| 12 | 81064132 | T | C | 1 | 1 | *PTPRQ* | non-synonymous SNV |
| 12 | 101736572 | C | T | 1 | 1 | *UTP20* | non-synonymous SNV |
| 12 | 124971081 | G | A | 1 | 1 | *NCOR2* | non-synonymous SNV |
| 13 | 32911792 | C | T | 1 | 1 | *BRCA2* | synonymous SNV |
| 13 | 32912917 | G | A | 1 | 1 | *BRCA2* | non-synonymous SNV |
| 13 | 38225438 | C | T | 1 | 1 | *TRPC4* | synonymous SNV |
| 14 | 33242932 | C | T | 1 | 1 | *AKAP6* | synonymous SNV |
| 14 | 94067110 | C | T | 1 | 1 | *UNC79* | non-synonymous SNV |
| 15 | 28230327 | C | T | 1 | 1 | *OCA2* | non-synonymous SNV |
| 15 | 88472488 | C | T | 1 | 1 | *NTRK3* | synonymous SNV |
| 16 | 2222342 | G | A | 1 | 1 | *TRAF7* | non-synonymous SNV |
| 16 | 4498815 | G | A | 1 | 1 | *DNAJA3* | non-synonymous SNV |
| 16 | 77317895 | A | C | 1 | 1 | *ADAMTS18* | non-synonymous SNV |
| 16 | 77359763 | C | T | 1 | 1 | *ADAMTS18* | non-synonymous SNV |
| 16 | 77401580 | G | A | 1 | 1 | *ADAMTS18* | non-synonymous SNV |
| 16 | 77465488 | C | T | 1 | 1 | *ADAMTS18* | non-synonymous SNV |
| 17 | 4713231 | C | T | 1 | 1 | *PLD2* | non-synonymous SNV |
| 17 | 7752185 | C | T | 1 | 1 | *KDM6B* | non-synonymous SNV |
| 17 | 8044451 | C | A | 1 | 1 | *PER1* | stopgain SNV |
| 17 | 10304272 | C | T | 1 | 1 | *MYH8* | non-synonymous SNV |
| 17 | 11556086 | G | A | 1 | 1 | *DNAH9* | non-synonymous SNV |
| 17 | 11607680 | G | A | 1 | 1 | *DNAH9* | non-synonymous SNV |
| 17 | 33498478 | C | T | 1 | 1 | *UNC45B* | synonymous SNV |
| 17 | 39925285 | G | T | 1 | 1 | *JUP* | non-synonymous SNV |
| 17 | 39925842 | G | A | 1 | 1 | *JUP* | non-synonymous SNV |
| 17 | 43192735 | C | A | 1 | 1 | *PLCD3* | unknown |
| 17 | 55339533 | C | T | 1 | 1 | *MSI2* | non-synonymous SNV |
| 17 | 58300869 | C | T | 1 | 1 | *USP32* | non-synonymous SNV |
| 17 | 78321556 | C | T | 1 | 1 | *RNF213* | non-synonymous SNV |
| 17 | 78727902 | C | T | 1 | 1 | *RPTOR* | synonymous SNV |
| 18 | 19070121 | C | T | 1 | 1 | *GREB1L* | non-synonymous SNV |
| 18 | 49867168 | G | A | 1 | 1 | *DCC* | non-synonymous SNV |
| 18 | 50432440 | G | A | 1 | 1 | *DCC* | non-synonymous SNV |
| 18 | 59157987 | C | T | 1 | 1 | *CDH20* | synonymous SNV |
| 18 | 59166637 | TCAAGACA | - | 1 | 1 | *CDH20* | frameshift deletion |
| 19 | 8973999 | G | A | 1 | 1 | *MUC16* | synonymous SNV |
| 19 | 9058036 | C | T | 1 | 1 | *MUC16* | non-synonymous SNV |
| 19 | 9065265 | G | A | 1 | 1 | *MUC16* | non-synonymous SNV |
| 19 | 9068476 | C | T | 1 | 1 | *MUC16* | non-synonymous SNV |
| 19 | 9069501 | G | A | 1 | 1 | *MUC16* | non-synonymous SNV |
| 19 | 9071521 | C | A | 1 | 1 | *MUC16* | stopgain SNV |
| 19 | 9076900 | G | A | 1 | 1 | *MUC16* | synonymous SNV |
| 19 | 9082401 | C | T | 1 | 1 | *MUC16* | synonymous SNV |
| 19 | 15289992 | C | T | 1 | 1 | *NOTCH3* | non-synonymous SNV |
| 19 | 48342950 | G | A | 1 | 1 | *CRX* | non-synonymous SNV |
| 19 | 57640074 | C | T | 1 | 1 | *USP29* | stopgain SNV |
| 20 | 1471998 | G | A | 1 | 1 | *SIRPB2* | non-synonymous SNV |
| 20 | 3016465 | C | T | 1 | 1 | *PTPRA* | synonymous SNV |
| 20 | 8745823 | G | A | 1 | 1 | *PLCB1* | synonymous SNV |
| 20 | 10036255 | C | T | 1 | 1 | *ANKRD5* | stopgain SNV |
| 20 | 16486446 | G | A | 1 | 1 | *KIF16B* | synonymous SNV |
| 20 | 33855117 | G | A | 1 | 1 | *MMP24* | synonymous SNV |
| 20 | 47633845 | G | A | 1 | 1 | *ARFGEF2* | non-synonymous SNV |
| 20 | 48166680 | CC | TT | 1 | 1 | *PTGIS* | non-frameshift substitution |
| 20 | 50701765 | G | A | 1 | 1 | *ZFP64* | synonymous SNV |
| 21 | 19713733 | G | A | 1 | 1 | *TMPRSS15* | non-synonymous SNV |
| 21 | 32575246 | G | A | 1 | 1 | *TIAM1* | non-synonymous SNV |
| 21 | 32638750 | C | T | 1 | 1 | *TIAM1* | non-synonymous SNV |
| 21 | 38269431 | C | T | 1 | 1 | *HLCS* | non-synonymous SNV |
| 21 | 39755817 | G | A | 1 | 1 | *ERG* | synonymous SNV |
| 22 | 36712697 | C | T | 1 | 1 | *MYH9* | synonymous SNV |
| X | 34961687 | C | T | 1 | 1 | *FAM47B* | non-synonymous SNV |
| X | 44929218 | T | A | 1 | 1 | *KDM6A* | non-synonymous SNV |
| X | 48661361 | C | T | 1 | 1 | *HDAC6* | synonymous SNV |
| X | 54783910 | G | A | 1 | 1 | *ITIH6* | non-synonymous SNV |
| X | 66863116 | C | T | 1 | 1 | *AR* | synonymous SNV |
| X | 84363970 | T | G | 1 | 1 | *SATL1* | non-synonymous SNV |
| X | 105152954 | - | G | 1 | 1 | *NRK* | frameshift insertion |
| X | 105152958 | C | A | 1 | 1 | *NRK* | non-synonymous SNV |
| X | 105153194 | C | T | 1 | 1 | *NRK* | stopgain SNV |
| X | 105197044 | G | A | 1 | 1 | *NRK* | non-synonymous SNV |
| X | 105199584 | G | A | 1 | 1 | *NRK* | non-synonymous SNV |
| X | 123775740 | G | A | 1 | 1 | *TENM1* | non-synonymous SNV |
| 1 | 32221979 | G | A | 1 | 0 | *BAI2* | synonymous SNV |
| 12 | 5154507 | C | T | 1 | 0 | *KCNA5* | synonymous SNV |
