# Supplementary material for: Molecular and genetic diversity in the metastatic process of melanoma
Source: J Pathol. 2014 Jan 27;233(1):39–50. doi: 10.1002/path.4318 (PMC4359751; doi:10.1002/path.4318)
Supplement: Supplementary file 14 — Coding sequence (exonic and splice site) mutations in patients 1–28 [file path0233-0039-sd14.zip › TableS14.doc]

**Table S14.** Exonic and splice site mutations in patient 12

| **Chrom** | **Position** | **Reference** | **Alternative** | **M1** | **M2** | **M3** | **Gene** | **Type** |
| --- | --- | --- | --- | --- | --- | --- | --- | --- |
| 1 | 36214049 | G | A | 1 | 1 | 1 | *CLSPN* | non-synonymous SNV |
| 1 | 65307233 | C | T | 1 | 1 | 1 | *JAK1* | non-synonymous SNV |
| 1 | 120458604 | G | A | 1 | 1 | 1 | *NOTCH2* | synonymous SNV |
| 1 | 154437849 | C | T | 1 | 1 | 1 | *IL6R* | non-synonymous SNV |
| 1 | 157514284 | C | T | 1 | 1 | 1 | *FCRL5* | synonymous SNV |
| 1 | 162735820 | C | T | 1 | 1 | 1 | *DDR2* | synonymous SNV |
| 1 | 196458977 | C | T | 1 | 1 | 1 | *KCNT2* | non-synonymous SNV |
| 1 | 202279367 | G | A | 1 | 1 | 1 | *LGR6* | synonymous SNV |
| 1 | 229683313 | G | A | 1 | 1 | 1 | *ABCB10* | non-synonymous SNV |
| 2 | 31589798 | G | A | 1 | 1 | 1 | *XDH* | non-synonymous SNV |
| 2 | 121744048 | C | T | 1 | 1 | 1 | *GLI2* | synonymous SNV |
| 2 | 135738957 | C | T | 1 | 1 | 1 | *YSK4* | synonymous SNV |
| 2 | 158140960 | G | A | 1 | 1 | 1 | *GALNT5* | non-synonymous SNV |
| 2 | 179417214 | C | T | 1 | 1 | 1 | *TTN* | non-synonymous SNV |
| 2 | 179633644 | G | A | 1 | 1 | 1 | *TTN* | synonymous SNV |
| 2 | 212288948 | A | G | 1 | 1 | 1 | *ERBB4* | non-synonymous SNV |
| 2 | 218713358 | C | T | 1 | 1 | 1 | *TNS1* | non-synonymous SNV |
| 3 | 10122819 | C | T | 1 | 1 | 1 | *FANCD2* | synonymous SNV |
| 3 | 39226601 | C | T | 1 | 1 | 1 | *XIRP1* | non-synonymous SNV |
| 3 | 58183660 | G | A | 1 | 1 | 1 | *DNASE1L3* | non-synonymous SNV |
| 3 | 129276033 | C | T | 1 | 1 | 1 | *PLXND1* | non-synonymous SNV |
| 3 | 194117773 | C | T | 1 | 1 | 1 | *GP5* | synonymous SNV |
| 4 | 62849213 | G | A | 1 | 1 | 1 | *LPHN3* | non-synonymous SNV |
| 4 | 167021913 | C | T | 1 | 1 | 1 | *TLL1* | non-synonymous SNV |
| 5 | 13793757 | G | A | 1 | 1 | 1 | *DNAH5* | synonymous SNV |
| 5 | 13914032 | C | T | 1 | 1 | 1 | *DNAH5* | synonymous SNV |
| 5 | 112174223 | C | T | 1 | 1 | 1 | *APC* | stopgain SNV |
| 5 | 149389994 | C | T | 1 | 1 | 1 | *HMGXB3* | synonymous SNV |
| 6 | 32190389 | T | C | 1 | 1 | 1 | *NOTCH4* | non-synonymous SNV |
| 6 | 43307692 | G | A | 1 | 1 | 1 | *ZNF318* | synonymous SNV |
| 6 | 69728363 | G | A | 1 | 1 | 1 | *BAI3* | synonymous SNV |
| 6 | 102503410 | G | A | 1 | 1 | 1 | *GRIK2* | synonymous SNV |
| 6 | 117638425 | G | A | 1 | 1 | 1 | *ROS1* | non-synonymous SNV |
| 6 | 117639403 | C | T | 1 | 1 | 1 | *ROS1* | non-synonymous SNV |
| 6 | 146351039 | C | T | 1 | 1 | 1 | *GRM1* | non-synonymous SNV |
| 6 | 146719982 | G | A | 1 | 1 | 1 | *GRM1* | non-synonymous SNV |
| 6 | 152577882 | G | A | 1 | 1 | 1 | *SYNE1* | stopgain SNV |
| 6 | 152738117 | C | T | 1 | 1 | 1 | *SYNE1* | non-synonymous SNV |
| 7 | 14613941 | T | A | 1 | 1 | 1 | *DGKB* | non-synonymous SNV |
| 7 | 72883883 | GGTCTTTGCAGTGATGGTTGAATC | - | 1 | 1 | 1 | *BAZ1B* | non-frameshift deletion |
| 7 | 140453135 | CA | TT | 1 | 1 | 1 | *BRAF* | non-frameshift substitution |
| 8 | 26623662 | C | T | 1 | 1 | 1 | *ADRA1A* | non-synonymous SNV |
| 8 | 48848400 | G | A | 1 | 1 | 1 | *PRKDC* | unknown |
| 9 | 21971017 | G | A | 1 | 1 | 1 | *CDKN2A* | non-synonymous SNV |
| 9 | 32630291 | C | T | 1 | 1 | 1 | *TAF1L* | non-synonymous SNV |
| 9 | 32634771 | C | T | 1 | 1 | 1 | *TAF1L* | synonymous SNV |
| 9 | 112144664 | C | T | 1 | 1 | 1 | *PTPN3* | non-synonymous SNV |
| 9 | 119202937 | C | T | 1 | 1 | 1 | *ASTN2* | non-synonymous SNV |
| 11 | 32610573 | C | T | 1 | 1 | 1 | *EIF3M* | synonymous SNV |
| 11 | 128680842 | C | T | 1 | 1 | 1 | *FLI1* | non-synonymous SNV |
| 12 | 7945655 | G | T | 1 | 1 | 1 | *NANOG* | non-synonymous SNV |
| 13 | 28877366 | C | G | 1 | 1 | 1 | *FLT1* | non-synonymous SNV |
| 13 | 38248428 | C | T | 1 | 1 | 1 | *TRPC4* | non-synonymous SNV |
| 14 | 33015398 | G | A | 1 | 1 | 1 | *AKAP6* | synonymous SNV |
| 14 | 70512907 | C | T | 1 | 1 | 1 | *SLC8A3* | stopgain SNV |
| 14 | 72090805 | C | T | 1 | 1 | 1 | *SIPA1L1* | non-synonymous SNV |
| 14 | 94088389 | G | A | 1 | 1 | 1 | *UNC79* | non-synonymous SNV |
| 15 | 28267652 | T | G | 1 | 1 | 1 | *OCA2* | non-synonymous SNV |
| 15 | 50792967 | C | T | 1 | 1 | 1 | *USP50* | synonymous SNV |
| 16 | 339474 | A | G | 1 | 1 | 1 | *AXIN1* | non-synonymous SNV |
| 16 | 62055079 | G | A | 1 | 1 | 1 | *CDH8* | non-synonymous SNV |
| 16 | 77334194 | G | A | 1 | 1 | 1 | *ADAMTS18* | synonymous SNV |
| 17 | 7578395 | G | A | 1 | 1 | 1 | *TP53* | non-synonymous SNV |
| 17 | 10405218 | G | A | 1 | 1 | 1 | *MYH1* | non-synonymous SNV |
| 17 | 11593114 | G | A | 1 | 1 | 1 | *DNAH9* | synonymous SNV |
| 17 | 38062116 | G | A | 1 | 1 | 1 | *GSDMB* | synonymous SNV |
| 17 | 41610258 | C | T | 1 | 1 | 1 | *ETV4* | non-synonymous SNV |
| 17 | 76824991 | G | A | 1 | 1 | 1 | *USP36* | stopgain SNV |
| 18 | 18964268 | G | A | 1 | 1 | 1 | *GREB1L* | non-synonymous SNV |
| 18 | 19020297 | C | T | 1 | 1 | 1 | *GREB1L* | synonymous SNV |
| 18 | 50432515 | C | T | 1 | 1 | 1 | *DCC* | non-synonymous SNV |
| 19 | 5225814 | G | A | 1 | 1 | 1 | *PTPRS* | synonymous SNV |
| 19 | 8998704 | G | A | 1 | 1 | 1 | *MUC16* | non-synonymous SNV |
| 19 | 9073481 | G | A | 1 | 1 | 1 | *MUC16* | synonymous SNV |
| 19 | 51128802 | G | A | 1 | 1 | 1 | *SYT3* | synonymous SNV |
| 19 | 55696969 | CC | TT | 1 | 1 | 1 | *PTPRH* | non-frameshift substitution |
| 19 | 56463969 | G | A | 1 | 1 | 1 | *NLRP8* | non-synonymous SNV |
| 20 | 33851702 | C | T | 1 | 1 | 1 | *MMP24* | non-synonymous SNV |
| 20 | 41076878 | C | T | 1 | 1 | 1 | *PTPRT* | synonymous SNV |
| 20 | 41306622 | T | C | 1 | 1 | 1 | *PTPRT* | non-synonymous SNV |
| 21 | 10951345 | G | A | 1 | 1 | 1 | *TPTE* | non-synonymous SNV |
| 21 | 10951406 | G | A | 1 | 1 | 1 | *TPTE* | synonymous SNV |
| 21 | 39671545 | C | T | 1 | 1 | 1 | *KCNJ15* | non-synonymous SNV |
| 21 | 39671615 | C | T | 1 | 1 | 1 | *KCNJ15* | synonymous SNV |
| 22 | 22285586 | G | A | 1 | 1 | 1 | *PPM1F* | synonymous SNV |
| 22 | 24125597 | G | A | 1 | 1 | 1 | *MMP11* | splicing |
| 22 | 50615408 | C | T | 1 | 1 | 1 | *PANX2* | synonymous SNV |
| X | 9864434 | C | G | 1 | 1 | 1 | *SHROOM2* | non-synonymous SNV |
| X | 48887952 | - | C | 1 | 1 | 1 | *TFE3* | frameshift insertion |
| X | 53610677 | C | T | 1 | 1 | 1 | *HUWE1* | synonymous SNV |
| X | 66931443 | C | A | 1 | 1 | 1 | *AR* | synonymous SNV |
| X | 75649430 | G | A | 1 | 1 | 1 | *MAGEE1* | synonymous SNV |
| X | 119410761 | G | A | 1 | 1 | 1 | *FAM70A* | synonymous SNV |
| X | 135429861 | C | A | 1 | 1 | 1 | *GPR112* | synonymous SNV |
| X | 135593216 | GG | AA | 1 | 1 | 1 | *HTATSF1* | non-frameshift substitution |
| X | 152482846 | G | T | 1 | 1 | 1 | *MAGEA1* | synonymous SNV |
| X | 153069647 | G | A | 1 | 1 | 1 | *PDZD4* | non-synonymous SNV |
| 9 | 78784630 | C | G | 1 | 1 | 1 | *PCSK5* | non-synonymous SNV |
| 5 | 43246150 | G | A | 1 | 1 | 1 | *NIM1* | synonymous SNV |
| 17 | 48265492 | C | T | 1 | 1 | 1 | *COL1A1* | non-synonymous SNV |
| X | 48681601 | G | A | 1 | 1 | 1 | *HDAC6* | non-synonymous SNV |
| 22 | 40815027 | G | A | 1 | 1 | 1 | *MKL1* | non-synonymous SNV |
| 17 | 73236130 | G | A | 1 | 1 | 1 | *GGA3* | synonymous SNV |
| 5 | 137803144 | C | T | 1 | 0 | 0 | *EGR1* | non-synonymous SNV |
| 5 | 178418903 | C | T | 1 | 0 | 0 | *GRM6* | non-synonymous SNV |
| 8 | 113267591 | G | T | 1 | 0 | 0 | *CSMD3* | non-synonymous SNV |
| 9 | 78965845 | G | T | 1 | 0 | 0 | *PCSK5* | splicing |
| 15 | 101434280 | T | C | 1 | 0 | 0 | *ALDH1A3* | non-synonymous SNV |
| 11 | 68115518 | G | A | 0 | 0 | 1 | *LRP5* | non-synonymous SNV |
| 1 | 149916897 | G | T | 0 | 0 | 1 | *OTUD7B* | non-synonymous SNV |
| 2 | 25470969 | G | T | 0 | 0 | 1 | *DNMT3A* | synonymous SNV |
| 5 | 137802791 | C | T | 0 | 0 | 1 | *EGR1* | non-synonymous SNV |
