# Supplementary material for: Molecular and genetic diversity in the metastatic process of melanoma
Source: J Pathol. 2014 Jan 27;233(1):39–50. doi: 10.1002/path.4318 (PMC4359751; doi:10.1002/path.4318)
Supplement: Supplementary file 14 — Coding sequence (exonic and splice site) mutations in patients 1–28 [file path0233-0039-sd14.zip › TableS15.doc]

**Table S15.** Exonic and splice site mutations in patient 14

| **Chrom** | **Position** | **Reference** | **Alternative** | **M1** | **M2** | **M3** | **Gene** | **Type** |
| --- | --- | --- | --- | --- | --- | --- | --- | --- |
| 1 | 3644763 | G | A | 1 | 1 | 1 | *TP73* | synonymous SNV |
| 1 | 19491445 | C | T | 1 | 1 | 1 | *UBR4* | synonymous SNV |
| 1 | 29638182 | G | A | 1 | 1 | 1 | *PTPRU* | non-synonymous SNV |
| 1 | 79002119 | G | A | 1 | 1 | 1 | *PTGFR* | non-synonymous SNV |
| 1 | 115256529 | T | C | 1 | 1 | 1 | *NRAS* | non-synonymous SNV |
| 1 | 153653749 | T | C | 1 | 1 | 1 | *NPR1* | non-synonymous SNV |
| 1 | 155252416 | C | T | 1 | 1 | 1 | *HCN3* | non-synonymous SNV |
| 1 | 157497650 | G | A | 1 | 1 | 1 | *FCRL5* | non-synonymous SNV |
| 1 | 196706613 | C | T | 1 | 1 | 1 | *CFH* | non-synonymous SNV |
| 1 | 196967406 | G | A | 1 | 1 | 1 | *CFHR5* | synonymous SNV |
| 1 | 228505396 | C | T | 1 | 1 | 1 | *OBSCN* | non-synonymous SNV |
| 2 | 26703741 | GC | TT | 1 | 1 | 1 | *OTOF* | non-frameshift substitution |
| 2 | 48958435 | G | A | 1 | 1 | 1 | *LHCGR* | non-synonymous SNV |
| 2 | 170063573 | G | A | 1 | 1 | 1 | *LRP2* | synonymous SNV |
| 2 | 170135892 | G | A | 1 | 1 | 1 | *LRP2* | non-synonymous SNV |
| 2 | 179393785 | C | T | 1 | 1 | 1 | *TTN* | non-synonymous SNV |
| 2 | 179416831 | C | T | 1 | 1 | 1 | *TTN* | non-synonymous SNV |
| 2 | 179433942 | C | T | 1 | 1 | 1 | *TTN* | synonymous SNV |
| 2 | 179561871 | G | A | 1 | 1 | 1 | *TTN* | non-synonymous SNV |
| 2 | 179595842 | C | T | 1 | 1 | 1 | *TTN* | synonymous SNV |
| 2 | 179599565 | C | T | 1 | 1 | 1 | *TTN* | non-synonymous SNV |
| 2 | 179613616 | G | A | 1 | 1 | 1 | *TTN* | non-synonymous SNV |
| 2 | 179621256 | C | T | 1 | 1 | 1 | *TTN* | synonymous SNV |
| 2 | 179639879 | C | T | 1 | 1 | 1 | *TTN* | non-synonymous SNV |
| 2 | 219920077 | G | A | 1 | 1 | 1 | *IHH* | non-synonymous SNV |
| 3 | 119634948 | G | A | 1 | 1 | 1 | *GSK3B* | non-synonymous SNV |
| 3 | 172520727 | G | A | 1 | 1 | 1 | *ECT2* | non-synonymous SNV |
| 3 | 182925550 | T | A | 1 | 1 | 1 | *MCF2L2* | non-synonymous SNV |
| 3 | 184298309 | C | T | 1 | 1 | 1 | *EPHB3* | synonymous SNV |
| 3 | 184298870 | C | T | 1 | 1 | 1 | *EPHB3* | synonymous SNV |
| 3 | 189612210 | C | T | 1 | 1 | 1 | *TP63* | synonymous SNV |
| 5 | 13824365 | C | T | 1 | 1 | 1 | *DNAH5* | non-synonymous SNV |
| 5 | 37059103 | AGATTCAGATTCAGATTC | - | 1 | 1 | 1 | *NIPBL* | non-frameshift deletion |
| 5 | 138160269 | C | T | 1 | 1 | 1 | *CTNNA1* | synonymous SNV |
| 6 | 36343697 | G | A | 1 | 1 | 1 | *ETV7* | non-synonymous SNV |
| 6 | 39602659 | G | C | 1 | 1 | 1 | *KIF6* | non-synonymous SNV |
| 6 | 47682640 | G | A | 1 | 1 | 1 | *GPR115* | stopgain SNV |
| 6 | 74502515 | T | C | 1 | 1 | 1 | *CD109* | synonymous SNV |
| 6 | 117631384 | C | T | 1 | 1 | 1 | *ROS1* | synonymous SNV |
| 7 | 5541263 | G | A | 1 | 1 | 1 | *FBXL18* | non-synonymous SNV |
| 7 | 18875567 | G | A | 1 | 1 | 1 | *HDAC9* | stopgain SNV |
| 7 | 99773426 | G | A | 1 | 1 | 1 | *GPC2* | synonymous SNV |
| 7 | 139657536 | C | T | 1 | 1 | 1 | *TBXAS1* | synonymous SNV |
| 8 | 56698886 | A | T | 1 | 1 | 1 | *TGS1* | non-synonymous SNV |
| 8 | 68939496 | C | T | 1 | 1 | 1 | *PREX2* | non-synonymous SNV |
| 8 | 99146818 | C | T | 1 | 1 | 1 | *POP1* | synonymous SNV |
| 10 | 8115941 | C | T | 1 | 1 | 1 | *GATA3* | synonymous SNV |
| 10 | 37438747 | G | A | 1 | 1 | 1 | *ANKRD30A* | non-synonymous SNV |
| 10 | 123256189 | C | T | 1 | 1 | 1 | *FGFR2* | non-synonymous SNV |
| 10 | 124380660 | G | A | 1 | 1 | 1 | *DMBT1* | non-synonymous SNV |
| 12 | 57849497 | C | T | 1 | 1 | 1 | *INHBE* | non-synonymous SNV |
| 12 | 70946780 | C | T | 1 | 1 | 1 | *PTPRB* | non-synonymous SNV |
| 12 | 80982137 | G | A | 1 | 1 | 1 | *PTPRQ* | synonymous SNV |
| 13 | 39918097 | C | T | 1 | 1 | 1 | *LHFP* | synonymous SNV |
| 14 | 70633828 | C | T | 1 | 1 | 1 | *SLC8A3* | non-synonymous SNV |
| 15 | 85402508 | G | A | 1 | 1 | 1 | *ALPK3* | synonymous SNV |
| 16 | 347145 | GG | AA | 1 | 1 | 1 | *AXIN1* | non-frameshift substitution |
| 16 | 2129375 | CC | TT | 1 | 1 | 1 | *TSC2* | non-frameshift substitution |
| 16 | 2129376 | C | T | 1 | 1 | 1 | *TSC2* | synonymous SNV |
| 16 | 15814104 | C | T | 1 | 1 | 1 | *MYH11* | synonymous SNV |
| 16 | 23079873 | G | A | 1 | 1 | 1 | *USP31* | stopgain SNV |
| 16 | 23641780 | A | G | 1 | 1 | 1 | *PALB2* | synonymous SNV |
| 16 | 24135302 | G | A | 1 | 1 | 1 | *PRKCB* | synonymous SNV |
| 17 | 11608362 | C | T | 1 | 1 | 1 | *DNAH9* | synonymous SNV |
| 17 | 11642338 | C | T | 1 | 1 | 1 | *DNAH9* | non-synonymous SNV |
| 17 | 37864712 | C | T | 1 | 1 | 1 | *ERBB2* | non-synonymous SNV |
| 17 | 45360795 | G | A | 1 | 1 | 1 | *ITGB3* | non-synonymous SNV |
| 17 | 59821939 | A | - | 1 | 1 | 1 | *BRIP1* | stopgain SNV |
| 18 | 33739766 | G | A | 1 | 1 | 1 | *ELP2* | non-synonymous SNV |
| 18 | 50918141 | G | A | 1 | 1 | 1 | *DCC* | non-synonymous SNV |
| 19 | 9056261 | G | A | 1 | 1 | 1 | *MUC16* | synonymous SNV |
| 19 | 15374311 | C | T | 1 | 1 | 1 | *BRD4* | non-synonymous SNV |
| 19 | 55451463 | G | A | 1 | 1 | 1 | *NLRP7* | synonymous SNV |
| 19 | 56243852 | G | A | 1 | 1 | 1 | *NLRP9* | stopgain SNV |
| 20 | 33027990 | C | T | 1 | 1 | 1 | *ITCH* | non-synonymous SNV |
| X | 40982957 | - | C | 1 | 1 | 1 | *USP9X* | frameshift insertion |
| X | 86890739 | C | T | 1 | 1 | 1 | *KLHL4* | non-synonymous SNV |
| X | 105011105 | G | A | 1 | 1 | 1 | *IL1RAPL2* | synonymous SNV |
| X | 123699397 | C | T | 1 | 1 | 1 | *ODZ1* | non-synonymous SNV |
| 9 | 98242860 | G | A | 1 | 0 | 1 | *PTCH1* | non-synonymous SNV |
| 8 | 93029545 | C | T | 0 | 1 | 1 | *RUNX1T1* | synonymous SNV |
| 1 | 91406555 | G | A | 0 | 0 | 1 | *ZNF644* | non-synonymous SNV |
| 17 | 8046771 | G | T | 0 | 0 | 1 | *PER1* | non-synonymous SNV |
| 22 | 29693831 | G | T | 0 | 0 | 1 | *EWSR1* | non-synonymous SNV |
| X | 49074227 | G | T | 0 | 0 | 1 | *CACNA1F* | non-synonymous SNV |
