# Supplementary material for: Molecular and genetic diversity in the metastatic process of melanoma
Source: J Pathol. 2014 Jan 27;233(1):39–50. doi: 10.1002/path.4318 (PMC4359751; doi:10.1002/path.4318)
Supplement: Supplementary file 14 — Coding sequence (exonic and splice site) mutations in patients 1–28 [file path0233-0039-sd14.zip › TableS16.doc]

**Table S16.** Exonic and splice site mutations in patient 15

| **Chrom** | **Position** | **Reference** | **Alternative** | **P** | **M1** | **Gene** | **Type** |
| --- | --- | --- | --- | --- | --- | --- | --- |
| 1 | 46874816 | C | T | 1 | 1 | *FAAH* | non-synonymous SNV |
| 1 | 115256530 | G | T | 1 | 1 | *NRAS* | non-synonymous SNV |
| 1 | 178412269 | C | T | 1 | 1 | *RASAL2* | synonymous SNV |
| 1 | 228559480 | C | T | 1 | 1 | *OBSCN* | non-synonymous SNV |
| 2 | 54175653 | T | C | 1 | 1 | *PSME4* | synonymous SNV |
| 2 | 179408378 | G | A | 1 | 1 | *TTN* | non-synonymous SNV |
| 2 | 179431712 | G | A | 1 | 1 | *TTN* | non-synonymous SNV |
| 2 | 179453417 | G | A | 1 | 1 | *TTN* | non-synonymous SNV |
| 2 | 179457798 | G | A | 1 | 1 | *TTN* | non-synonymous SNV |
| 2 | 179479467 | G | A | 1 | 1 | *TTN* | synonymous SNV |
| 2 | 179516182 | T | C | 1 | 1 | *TTN* | non-synonymous SNV |
| 2 | 179542456 | C | T | 1 | 1 | *TTN* | non-synonymous SNV |
| 2 | 179556824 | G | A | 1 | 1 | *TTN* | non-synonymous SNV |
| 2 | 179587127 | C | T | 1 | 1 | *TTN* | non-synonymous SNV |
| 2 | 179610843 | C | T | 1 | 1 | *TTN* | synonymous SNV |
| 2 | 238285641 | G | A | 1 | 1 | *COL6A3* | synonymous SNV |
| 3 | 12633234 | G | A | 1 | 1 | *RAF1* | non-synonymous SNV |
| 3 | 36759619 | G | A | 1 | 1 | *DCLK3* | synonymous SNV |
| 3 | 37365637 | T | A | 1 | 1 | *GOLGA4* | non-synonymous SNV |
| 3 | 122288833 | A | G | 1 | 1 | *DTX3L* | non-synonymous SNV |
| 3 | 134911467 | G | A | 1 | 1 | *EPHB1* | synonymous SNV |
| 3 | 189526232 | C | T | 1 | 1 | *TP63* | non-synonymous SNV |
| 3 | 196612776 | C | T | 1 | 1 | *SENP5* | non-synonymous SNV |
| 5 | 21842425 | G | A | 1 | 1 | *CDH12* | non-synonymous SNV |
| 5 | 43508719 | G | T | 1 | 1 | *C5orf34* | non-synonymous SNV |
| 5 | 114482996 | G | A | 1 | 1 | *TRIM36* | synonymous SNV |
| 6 | 43173780 | C | T | 1 | 1 | *CUL9* | non-synonymous SNV |
| 6 | 46609998 | C | T | 1 | 1 | *CYP39A1* | non-synonymous SNV |
| 6 | 47682505 | G | A | 1 | 1 | *GPR115* | synonymous SNV |
| 6 | 69949092 | G | A | 1 | 1 | *BAI3* | non-synonymous SNV |
| 6 | 70897897 | G | A | 1 | 1 | *COL19A1* | non-synonymous SNV |
| 6 | 160638499 | G | A | 1 | 1 | *SLC22A2* | non-synonymous SNV |
| 7 | 23292967 | A | T | 1 | 1 | *GPNMB* | non-synonymous SNV |
| 7 | 113519153 | G | A | 1 | 1 | *PPP1R3A* | non-synonymous SNV |
| 7 | 143039518 | C | T | 1 | 1 | *CLCN1* | non-synonymous SNV |
| 7 | 151876987 | G | A | 1 | 1 | *MLL3* | synonymous SNV |
| 8 | 48761735 | G | A | 1 | 1 | *PRKDC* | unknown |
| 8 | 113529334 | C | T | 1 | 1 | *CSMD3* | non-synonymous SNV |
| 8 | 114326957 | G | A | 1 | 1 | *CSMD3* | non-synonymous SNV |
| 10 | 18266965 | G | A | 1 | 1 | *SLC39A12* | non-synonymous SNV |
| 10 | 37425564 | G | A | 1 | 1 | *ANKRD30A* | non-synonymous SNV |
| 11 | 44104823 | C | T | 1 | 1 | *ACCS* | non-synonymous SNV |
| 11 | 77629911 | GT | AA | 1 | 1 | *INTS4* | non-frameshift substitution |
| 11 | 108180998 | A | C | 1 | 1 | *ATM* | synonymous SNV |
| 11 | 113934947 | C | T | 1 | 1 | *ZBTB16* | non-synonymous SNV |
| 11 | 121424683 | C | T | 1 | 1 | *SORL1* | synonymous SNV |
| 11 | 125848220 | C | T | 1 | 1 | *CDON* | non-synonymous SNV |
| 12 | 70928676 | G | A | 1 | 1 | *PTPRB* | synonymous SNV |
| 12 | 80900373 | C | T | 1 | 1 | *PTPRQ* | synonymous SNV |
| 12 | 80982048 | G | A | 1 | 1 | *PTPRQ* | non-synonymous SNV |
| 12 | 112915470 | T | C | 1 | 1 | *PTPN11* | non-synonymous SNV |
| 14 | 50626170 | A | G | 1 | 1 | *SOS2* | synonymous SNV |
| 15 | 88679265 | G | A | 1 | 1 | *NTRK3* | synonymous SNV |
| 16 | 10032089 | G | A | 1 | 1 | *GRIN2A* | non-synonymous SNV |
| 17 | 7579389 | G | A | 1 | 1 | *TP53* | stopgain SNV |
| 17 | 8785016 | C | T | 1 | 1 | *PIK3R5* | synonymous SNV |
| 18 | 18534800 | A | G | 1 | 1 | *ROCK1* | non-synonymous SNV |
| 18 | 43523226 | G | A | 1 | 1 | *EPG5* | non-synonymous SNV |
| 18 | 50912472 | C | T | 1 | 1 | *DCC* | non-synonymous SNV |
| 18 | 59217382 | G | A | 1 | 1 | *CDH20* | non-synonymous SNV |
| 19 | 9046390 | G | A | 1 | 1 | *MUC16* | synonymous SNV |
| 19 | 9047189 | G | A | 1 | 1 | *MUC16* | non-synonymous SNV |
| 19 | 57642163 | G | A | 1 | 1 | *USP29* | non-synonymous SNV |
| 20 | 8862337 | C | T | 1 | 1 | *PLCB1* | synonymous SNV |
| 20 | 9498834 | T | G | 1 | 1 | *LAMP5* | non-synonymous SNV |
| 20 | 32878143 | G | A | 1 | 1 | *AHCY* | non-synonymous SNV |
| X | 49076094 | G | A | 1 | 1 | *CACNA1F* | non-synonymous SNV |
| X | 135428293 | C | A | 1 | 1 | *GPR112* | non-synonymous SNV |
| 1 | 196953106 | C | A | 1 | 0 | *CFHR5* | non-synonymous SNV |
| 2 | 29497971 | G | A | 1 | 0 | *ALK* | non-synonymous SNV |
| 6 | 39607479 | C | T | 1 | 0 | *KIF6* | synonymous SNV |
| X | 135485440 | G | A | 1 | 0 | *GPR112* | synonymous SNV |
| 1 | 246027125 | - | T | 0 | 1 | *SMYD3* | frameshift insertion |
| 5 | 178408667 | G | A | 0 | 1 | *GRM6* | synonymous SNV |
| 7 | 78636471 | T | G | 0 | 1 | *MAGI2* | non-synonymous SNV |
| X | 76952192 | C | G | 0 | 1 | *ATRX* | non-synonymous SNV |
