# Supplementary material for: Molecular and genetic diversity in the metastatic process of melanoma
Source: J Pathol. 2014 Jan 27;233(1):39–50. doi: 10.1002/path.4318 (PMC4359751; doi:10.1002/path.4318)
Supplement: Supplementary file 14 — Coding sequence (exonic and splice site) mutations in patients 1–28 [file path0233-0039-sd14.zip › TableS17.doc]

**Table S17.** Exonic and splice site mutations in patient 16

| **Chrom** | **Position** | **Reference** | **Alternative** | **M1** | **M2** | **Gene** | **Type** |
| --- | --- | --- | --- | --- | --- | --- | --- |
| 1 | 11580888 | C | T | 1 | 1 | *PTCHD2* | non-synonymous SNV |
| 1 | 33134655 | C | T | 1 | 1 | *RBBP4* | non-synonymous SNV |
| 1 | 157509053 | G | A | 1 | 1 | *FCRL5* | non-synonymous SNV |
| 1 | 196577427 | CC | TT | 1 | 1 | *KCNT2* | non-frameshift substitution |
| 2 | 48915882 | G | A | 1 | 1 | *LHCGR* | non-synonymous SNV |
| 2 | 179436997 | C | T | 1 | 1 | *TTN* | non-synonymous SNV |
| 2 | 179445304 | T | C | 1 | 1 | *TTN* | non-synonymous SNV |
| 2 | 179505295 | C | T | 1 | 1 | *TTN* | non-synonymous SNV |
| 2 | 179587962 | G | A | 1 | 1 | *TTN* | non-synonymous SNV |
| 2 | 179588022 | C | T | 1 | 1 | *TTN* | non-synonymous SNV |
| 2 | 179606100 | G | A | 1 | 1 | *TTN* | non-synonymous SNV |
| 2 | 179615320 | C | T | 1 | 1 | *TTN* | non-synonymous SNV |
| 2 | 238287555 | C | T | 1 | 1 | *COL6A3* | non-synonymous SNV |
| 2 | 238303795 | C | T | 1 | 1 | *COL6A3* | stopgain SNV |
| 3 | 52019887 | C | T | 1 | 1 | *ACY1* | non-synonymous SNV |
| 3 | 97311576 | C | T | 1 | 1 | *EPHA6* | non-synonymous SNV |
| 3 | 124114113 | T | C | 1 | 1 | *KALRN* | synonymous SNV |
| 3 | 185766549 | G | A | 1 | 1 | *ETV5* | non-synonymous SNV |
| 4 | 55133623 | C | T | 1 | 1 | *PDGFRA* | synonymous SNV |
| 5 | 34945901 | C | T | 1 | 1 | *DNAJC21* | non-synonymous SNV |
| 5 | 133644344 | G | T | 1 | 1 | *CDKL3* | non-synonymous SNV |
| 6 | 43325106 | G | A | 1 | 1 | *ZNF318* | stopgain SNV |
| 6 | 70070772 | G | A | 1 | 1 | *BAI3* | non-synonymous SNV |
| 6 | 146719987 | C | G | 1 | 1 | *GRM1* | non-synonymous SNV |
| 7 | 37916582 | C | T | 1 | 1 | *TXNDC3* | stopgain SNV |
| 7 | 47869117 | C | T | 1 | 1 | *PKD1L1* | non-synonymous SNV |
| 7 | 47884605 | G | A | 1 | 1 | *PKD1L1* | non-synonymous SNV |
| 7 | 77256468 | C | T | 1 | 1 | *PTPN12* | non-synonymous SNV |
| 7 | 126746650 | G | A | 1 | 1 | *GRM8* | synonymous SNV |
| 7 | 140453136 | AC | TT | 1 | 1 | *BRAF* | non-frameshift substitution |
| 8 | 57079731 | G | A | 1 | 1 | *PLAG1* | non-synonymous SNV |
| 10 | 37508414 | G | A | 1 | 1 | *ANKRD30A* | synonymous SNV |
| 10 | 89711875 | G | A | 1 | 1 | *PTEN* | non-synonymous SNV |
| 10 | 123847433 | G | A | 1 | 1 | *TACC2* | synonymous SNV |
| 10 | 124380663 | C | T | 1 | 1 | *DMBT1* | non-synonymous SNV |
| 11 | 32413606 | C | T | 1 | 1 | *WT1* | synonymous SNV |
| 11 | 102567435 | CC | TT | 1 | 1 | *MMP27* | non-frameshift substitution |
| 11 | 106558439 | G | A | 1 | 1 | *GUCY1A2* | non-synonymous SNV |
| 11 | 118347560 | C | T | 1 | 1 | *MLL* | non-synonymous SNV |
| 11 | 120980037 | G | A | 1 | 1 | *TECTA* | non-synonymous SNV |
| 12 | 49418424 | G | A | 1 | 1 | *MLL2* | non-synonymous SNV |
| 12 | 80862580 | G | A | 1 | 1 | *PTPRQ* | synonymous SNV |
| 12 | 120128199 | G | A | 1 | 1 | *CIT* | synonymous SNV |
| 12 | 123213940 | G | A | 1 | 1 | *HCAR1* | non-synonymous SNV |
| 13 | 38211707 | C | T | 1 | 1 | *TRPC4* | non-synonymous SNV |
| 13 | 102029117 | C | T | 1 | 1 | *NALCN* | non-synonymous SNV |
| 15 | 50923650 | C | T | 1 | 1 | *TRPM7* | non-synonymous SNV |
| 16 | 1818787 | C | T | 1 | 1 | *MAPK8IP3* | non-synonymous SNV |
| 16 | 24226094 | C | T | 1 | 1 | *PRKCB* | non-synonymous SNV |
| 16 | 57060811 | C | T | 1 | 1 | *NLRC5* | synonymous SNV |
| 17 | 5462591 | C | T | 1 | 1 | *NLRP1* | stopgain SNV |
| 17 | 11593327 | C | T | 1 | 1 | *DNAH9* | synonymous SNV |
| 17 | 11671886 | C | T | 1 | 1 | *DNAH9* | synonymous SNV |
| 17 | 26094815 | G | C | 1 | 1 | *NOS2* | non-synonymous SNV |
| 17 | 48264479 | G | A | 1 | 1 | *COL1A1* | non-synonymous SNV |
| 17 | 72842997 | C | T | 1 | 1 | *GRIN2C* | synonymous SNV |
| 19 | 9048767 | G | A | 1 | 1 | *MUC16* | non-synonymous SNV |
| 19 | 9056817 | G | A | 1 | 1 | *MUC16* | non-synonymous SNV |
| 19 | 9060771 | A | C | 1 | 1 | *MUC16* | non-synonymous SNV |
| 19 | 9066108 | G | A | 1 | 1 | *MUC16* | non-synonymous SNV |
| 19 | 9075567 | G | A | 1 | 1 | *MUC16* | non-synonymous SNV |
| 19 | 10468447 | G | A | 1 | 1 | *TYK2* | non-synonymous SNV |
| 20 | 8608956 | G | A | 1 | 1 | *PLCB1* | non-synonymous SNV |
| 20 | 8665678 | C | T | 1 | 1 | *PLCB1* | non-synonymous SNV |
| 21 | 40190496 | C | T | 1 | 1 | *ETS2* | non-synonymous SNV |
| 4 | 103500167 | C | T | 1 | 0 | *NFKB1* | non-synonymous SNV |
| 2 | 179440762 | A | - | 0 | 1 | *TTN* | frameshift deletion |
