# Supplementary material for: Molecular and genetic diversity in the metastatic process of melanoma
Source: J Pathol. 2014 Jan 27;233(1):39–50. doi: 10.1002/path.4318 (PMC4359751; doi:10.1002/path.4318)
Supplement: Supplementary file 14 — Coding sequence (exonic and splice site) mutations in patients 1–28 [file path0233-0039-sd14.zip › TableS18.doc]

**Table S18.** Exonic and splice site mutations in patient 18

| **Chrom** | **Position** | **Reference** | **Alternative** | **M2** | **M3** | **Gene** | **Type** |
| --- | --- | --- | --- | --- | --- | --- | --- |
| 1 | 27683576 | G | A | 1 | 1 | *MAP3K6* | synonymous SNV |
| 1 | 60521014 | G | A | 1 | 1 | *C1orf87* | synonymous SNV |
| 1 | 103431095 | C | T | 1 | 1 | *COL11A1* | non-synonymous SNV |
| 1 | 115256528 | TTG | CTT | 1 | 1 | *NRAS* | non-frameshift substitution |
| 1 | 157504599 | C | T | 1 | 1 | *FCRL5* | non-synonymous SNV |
| 2 | 131128495 | C | T | 1 | 1 | *PTPN18* | synonymous SNV |
| 2 | 179429522 | C | T | 1 | 1 | *TTN* | non-synonymous SNV |
| 2 | 179585189 | G | A | 1 | 1 | *TTN* | non-synonymous SNV |
| 2 | 212285211 | G | A | 1 | 1 | *ERBB4* | synonymous SNV |
| 2 | 220155319 | C | T | 1 | 1 | *PTPRN* | non-synonymous SNV |
| 4 | 111470941 | G | A | 1 | 1 | *ENPEP* | stopgain SNV |
| 5 | 13719024 | C | T | 1 | 1 | *DNAH5* | non-synonymous SNV |
| 5 | 139917808 | C | T | 1 | 1 | *ANKHD1,ANKHD1-EIF4EBP3* | non-synonymous SNV |
| 5 | 180048602 | C | T | 1 | 1 | *FLT4* | non-synonymous SNV |
| 6 | 55039466 | C | T | 1 | 1 | *HCRTR2* | synonymous SNV |
| 6 | 128643355 | G | A | 1 | 1 | *PTPRK* | synonymous SNV |
| 6 | 129826443 | G | A | 1 | 1 | *LAMA2* | synonymous SNV |
| 6 | 160664704 | C | T | 1 | 1 | *SLC22A2* | non-synonymous SNV |
| 7 | 55211055 | CC | TT | 1 | 1 | *EGFR* | non-frameshift substitution |
| 7 | 116398656 | C | T | 1 | 1 | *MET* | non-synonymous SNV |
| 8 | 17868834 | A | C | 1 | 1 | *PCM1* | non-synonymous SNV |
| 9 | 32633214 | C | T | 1 | 1 | *TAF1L* | synonymous SNV |
| 9 | 120476546 | C | T | 1 | 1 | *TLR4* | non-synonymous SNV |
| 10 | 37430789 | G | A | 1 | 1 | *ANKRD30A* | non-synonymous SNV |
| 11 | 7059851 | G | A | 1 | 1 | *NLRP14* | non-synonymous SNV |
| 11 | 7059931 | G | A | 1 | 1 | *NLRP14* | non-synonymous SNV |
| 11 | 7586876 | C | T | 1 | 1 | *PPFIBP2* | non-synonymous SNV |
| 11 | 74873780 | G | A | 1 | 1 | *SLCO2B1* | non-synonymous SNV |
| 11 | 121429470 | G | A | 1 | 1 | *SORL1* | non-synonymous SNV |
| 12 | 53162625 | C | T | 1 | 1 | *KRT76* | non-synonymous SNV |
| 12 | 70946584 | C | T | 1 | 1 | *PTPRB* | non-synonymous SNV |
| 12 | 71003039 | C | T | 1 | 1 | *PTPRB* | stopgain SNV |
| 14 | 102252464 | C | T | 1 | 1 | *PPP2R5C* | non-synonymous SNV |
| 15 | 85383178 | G | A | 1 | 1 | *ALPK3* | non-synonymous SNV |
| 15 | 96875471 | G | A | 1 | 1 | *NR2F2* | non-synonymous SNV |
| 16 | 85699818 | G | A | 1 | 1 | *KIAA0182* | non-synonymous SNV |
| 17 | 38064439 | G | A | 1 | 1 | *GSDMB* | synonymous SNV |
| 19 | 9063955 | G | A | 1 | 1 | *MUC16* | non-synonymous SNV |
| 19 | 9085017 | G | A | 1 | 1 | *MUC16* | synonymous SNV |
| 19 | 40741834 | G | A | 1 | 1 | *AKT2* | synonymous SNV |
| 20 | 40709519 | C | T | 1 | 1 | *PTPRT* | synonymous SNV |
| X | 78216386 | C | T | 1 | 1 | *P2RY10* | synonymous SNV |
| 9 | 84226723 | G | A | 1 | 1 | *TLE1* | synonymous SNV |
| 3 | 122419967 | G | A | 1 | 0 | *PARP14* | non-synonymous SNV |
| 17 | 11738052 | C | T | 1 | 0 | *DNAH9* | non-synonymous SNV |
| 18 | 50450157 | G | A | 1 | 0 | *DCC* | non-synonymous SNV |
| 18 | 59166450 | C | T | 1 | 0 | *CDH20* | non-synonymous SNV |
| 6 | 109769992 | G | C | 0 | 1 | *MICAL1* | non-synonymous SNV |
| 16 | 68844111 | C | T | 0 | 1 | *CDH1* | synonymous SNV |
