# Supplementary material for: Molecular and genetic diversity in the metastatic process of melanoma
Source: J Pathol. 2014 Jan 27;233(1):39–50. doi: 10.1002/path.4318 (PMC4359751; doi:10.1002/path.4318)
Supplement: Supplementary file 14 — Coding sequence (exonic and splice site) mutations in patients 1–28 [file path0233-0039-sd14.zip › TableS19.doc]

**Table S19.** Exonic and splice site mutations in patient 19

| **Chrom** | **Position** | **Reference** | **Alternative** | **M1** | **M2** | **Gene** | **Type** |
| --- | --- | --- | --- | --- | --- | --- | --- |
| 1 | 115469121 | G | A | 1 | 1 | *SYCP1* | splicing |
| 1 | 196648785 | GG | AA | 1 | 1 | *CFH* | non-frameshift substitution |
| 2 | 26502152 | CC | TT | 1 | 1 | *HADHB* | non-frameshift substitution |
| 2 | 179408030 | C | T | 1 | 1 | *TTN* | non-synonymous SNV |
| 2 | 179614350 | G | A | 1 | 1 | *TTN* | synonymous SNV |
| 3 | 436531 | G | A | 1 | 1 | *CHL1* | non-synonymous SNV |
| 3 | 48606834 | C | T | 1 | 1 | *COL7A1* | non-synonymous SNV |
| 3 | 96963003 | C | T | 1 | 1 | *EPHA6* | non-synonymous SNV |
| 4 | 175898132 | C | T | 1 | 1 | *ADAM29* | non-synonymous SNV |
| 5 | 21842363 | C | T | 1 | 1 | *CDH12* | non-synonymous SNV |
| 6 | 30672858 | G | A | 1 | 1 | *MDC1* | non-synonymous SNV |
| 6 | 133804212 | C | T | 1 | 1 | *EYA4* | non-synonymous SNV |
| 7 | 100410605 | C | T | 1 | 1 | *EPHB4* | non-synonymous SNV |
| 7 | 140453136 | A | T | 1 | 1 | *BRAF* | non-synonymous SNV |
| 8 | 41580657 | GG | AA | 1 | 1 | *ANK1* | immediate stopgain |
| 9 | 8486081 | G | A | 1 | 1 | *PTPRD* | synonymous SNV |
| 9 | 120476865 | C | T | 1 | 1 | *TLR4* | non-synonymous SNV |
| 9 | 133759511 | C | T | 1 | 1 | *ABL1* | non-synonymous SNV |
| 12 | 121766273 | G | A | 1 | 1 | *ANAPC5* | non-synonymous SNV |
| 14 | 50626720 | G | A | 1 | 1 | *SOS2* | synonymous SNV |
| 15 | 31332445 | G | A | 1 | 1 | *TRPM1* | non-synonymous SNV |
| 16 | 15842005 | G | A | 1 | 1 | *MYH11* | synonymous SNV |
| 16 | 23640967 | G | A | 1 | 1 | *PALB2* | synonymous SNV |
| 17 | 10304652 | C | T | 1 | 1 | *MYH8* | synonymous SNV |
| 17 | 37618522 | T | G | 1 | 1 | *CDK12* | synonymous SNV |
| 18 | 19378178 | A | G | 1 | 1 | *MIB1* | non-synonymous SNV |
| 19 | 9080524 | C | T | 1 | 1 | *MUC16* | synonymous SNV |
| 19 | 37904434 | G | A | 1 | 1 | *ZNF569* | non-synonymous SNV |
| 19 | 55445922 | C | T | 1 | 1 | *NLRP7* | synonymous SNV |
| 19 | 56243696 | C | T | 1 | 1 | *NLRP9* | non-synonymous SNV |
| X | 49032529 | G | A | 1 | 1 | *PRICKLE3* | synonymous SNV |
| X | 84362955 | G | A | 1 | 1 | *SATL1* | synonymous SNV |
| 1 | 228404968 | G | A | 1 | 0 | *OBSCN* | non-synonymous SNV |
| 5 | 13807703 | G | A | 1 | 0 | *DNAH5* | synonymous SNV |
| 7 | 91660830 | C | T | 1 | 0 | *AKAP9* | non-synonymous SNV |
| 20 | 3562805 | G | A | 1 | 0 | *ATRN* | stopgain SNV |
| X | 118148196 | C | T | 1 | 0 | *LONRF3* | synonymous SNV |
| 2 | 179422090 | G | A | 0 | 1 | *TTN* | non-synonymous SNV |
| 4 | 155506777 | T | A | 0 | 1 | *FGA* | stopgain SNV |
| 8 | 114111012 | A | C | 0 | 1 | *CSMD3* | non-synonymous SNV |
