# Supplementary material for: Molecular and genetic diversity in the metastatic process of melanoma
Source: J Pathol. 2014 Jan 27;233(1):39–50. doi: 10.1002/path.4318 (PMC4359751; doi:10.1002/path.4318)
Supplement: Supplementary file 14 — Coding sequence (exonic and splice site) mutations in patients 1–28 [file path0233-0039-sd14.zip › TableS20.doc]

**Table S20.** Exonic and splice site mutations in patient 20

| **Chrom** | **Position** | **Reference** | **Alternative** | **M1** | **M2** | **Gene** | **Type** |
| --- | --- | --- | --- | --- | --- | --- | --- |
| 1 | 160328025 | C | T | 1 | 1 | *NCSTN* | synonymous SNV |
| 2 | 47604207 | G | A | 1 | 1 | *EPCAM* | synonymous SNV |
| 3 | 12641687 | C | T | 1 | 1 | *RAF1* | synonymous SNV |
| 3 | 52620665 | C | A | 1 | 1 | *PBRM1* | non-synonymous SNV |
| 4 | 40104206 | T | C | 1 | 1 | *N4BP2* | synonymous SNV |
| 5 | 35871189 | C | T | 1 | 1 | *IL7R* | synonymous SNV |
| 5 | 56168545 | - | T | 1 | 1 | *MAP3K1* | frameshift insertion |
| 5 | 82940473 | G | A | 1 | 1 | *HAPLN1* | non-synonymous SNV |
| 6 | 70639508 | G | A | 1 | 1 | *COL19A1* | synonymous SNV |
| 7 | 18833030 | C | T | 1 | 1 | *HDAC9* | synonymous SNV |
| 8 | 113323291 | G | A | 1 | 1 | *CSMD3* | stopgain SNV |
| 9 | 120475701 | C | T | 1 | 1 | *TLR4* | non-synonymous SNV |
| 9 | 139753694 | GCCCTGGGGCCCATT | - | 1 | 1 | *MAMDC4* | non-frameshift deletion |
| 10 | 37508214 | G | C | 1 | 1 | *ANKRD30A* | non-synonymous SNV |
| 11 | 20676353 | G | A | 1 | 1 | *SLC6A5* | non-synonymous SNV |
| 12 | 25398281 | C | T | 1 | 1 | *KRAS* | non-synonymous SNV |
| 12 | 53609547 | CTC | - | 1 | 1 | *RARG* | non-frameshift deletion |
| 14 | 39716481 | G | A | 1 | 1 | *MIA2* | non-synonymous SNV |
| 16 | 23083502 | G | A | 1 | 1 | *USP31* | synonymous SNV |
| 16 | 68847322 | - | T | 1 | 1 | *CDH1* | frameshift insertion |
| 19 | 11141561 | C | T | 1 | 1 | *SMARCA4* | non-synonymous SNV |
| 19 | 56244747 | C | T | 1 | 1 | *NLRP9* | synonymous SNV |
| 20 | 40743863 | G | A | 1 | 1 | *PTPRT* | synonymous SNV |
| 21 | 40194690 | C | T | 1 | 1 | *ETS2* | synonymous SNV |
| 22 | 29935382 | G | T | 1 | 1 | *THOC5* | non-synonymous SNV |
| X | 54785327 | A | T | 1 | 1 | *ITIH6* | non-synonymous SNV |
| 2 | 215593459 | G | A | 1 | 0 | *BARD1* | non-synonymous SNV |
| 6 | 4032711 | T | C | 1 | 0 | *PRPF4B* | synonymous SNV |
| 8 | 24775458 | G | C | 1 | 0 | *NEFM* | non-synonymous SNV |
| 10 | 124345699 | C | T | 1 | 0 | *DMBT1* | non-synonymous SNV |
| 14 | 79270093 | G | A | 1 | 0 | *NRXN3* | synonymous SNV |
| 16 | 9858258 | C | T | 1 | 0 | *GRIN2A* | non-synonymous SNV |
| 1 | 32198577 | C | T | 0 | 1 | *BAI2* | non-synonymous SNV |
| 1 | 209907730 | GG | AA | 0 | 1 | *HSD11B1* | non-frameshift substitution |
| 2 | 42990912 | G | A | 0 | 1 | *OXER1* | synonymous SNV |
| 2 | 179640729 | T | G | 0 | 1 | *TTN* | synonymous SNV |
| 3 | 41274899 | G | T | 0 | 1 | *CTNNB1* | non-synonymous SNV |
| 3 | 49147765 | G | C | 0 | 1 | *USP19* | non-synonymous SNV |
| 4 | 104079884 | A | C | 0 | 1 | *CENPE* | non-synonymous SNV |
| 4 | 153247223 | C | G | 0 | 1 | *FBXW7* | non-synonymous SNV |
| 5 | 13868072 | T | C | 0 | 1 | *DNAH5* | synonymous SNV |
| 6 | 36336736 | A | G | 0 | 1 | *ETV7* | synonymous SNV |
| 8 | 12946198 | G | A | 0 | 1 | *DLC1* | non-synonymous SNV |
| 8 | 48769776 | A | C | 0 | 1 | *PRKDC* | unknown |
| 8 | 113988187 | G | T | 0 | 1 | *CSMD3* | non-synonymous SNV |
| 17 | 11696829 | T | G | 0 | 1 | *DNAH9* | non-synonymous SNV |
| 19 | 9047474 | T | A | 0 | 1 | *MUC16* | non-synonymous SNV |
| 22 | 24125645 | G | A | 0 | 1 | *MMP11* | non-synonymous SNV |
