# Supplementary material for: Molecular and genetic diversity in the metastatic process of melanoma
Source: J Pathol. 2014 Jan 27;233(1):39–50. doi: 10.1002/path.4318 (PMC4359751; doi:10.1002/path.4318)
Supplement: Supplementary file 14 — Coding sequence (exonic and splice site) mutations in patients 1–28 [file path0233-0039-sd14.zip › TableS21.doc]

**Table S21.** Exonic and splice site mutations in patient 22

| **Chrom** | **Position** | **Reference** | **Alternative** | **M1** | **M2** | **Gene** | **Type** |
| --- | --- | --- | --- | --- | --- | --- | --- |
| 1 | 44019512 | C | T | 1 | 1 | *PTPRF* | synonymous SNV |
| 1 | 46871322 | C | T | 1 | 1 | *FAAH* | non-synonymous SNV |
| 1 | 115456618 | G | A | 1 | 1 | *SYCP1* | non-synonymous SNV |
| 1 | 115537535 | A | C | 1 | 1 | *SYCP1* | synonymous SNV |
| 1 | 183867025 | GG | AA | 1 | 1 | *RGL1* | non-frameshift substitution |
| 1 | 196227375 | C | T | 1 | 1 | *KCNT2* | non-synonymous SNV |
| 1 | 196971616 | A | C | 1 | 1 | *CFHR5* | non-synonymous SNV |
| 1 | 198677285 | G | A | 1 | 1 | *PTPRC* | non-synonymous SNV |
| 1 | 202245483 | G | A | 1 | 1 | *LGR6* | non-synonymous SNV |
| 1 | 228480480 | C | T | 1 | 1 | *OBSCN* | synonymous SNV |
| 2 | 24253981 | G | C | 1 | 1 | *C2orf44* | non-synonymous SNV |
| 2 | 26690092 | T | A | 1 | 1 | *OTOF* | stopgain SNV |
| 2 | 60688882 | G | A | 1 | 1 | *BCL11A* | stopgain SNV |
| 2 | 121747745 | G | A | 1 | 1 | *GLI2* | non-synonymous SNV |
| 2 | 158142560 | C | T | 1 | 1 | *GALNT5* | non-synonymous SNV |
| 2 | 179439339 | C | T | 1 | 1 | *TTN* | synonymous SNV |
| 2 | 179567228 | C | T | 1 | 1 | *TTN* | non-synonymous SNV |
| 2 | 179610479 | C | T | 1 | 1 | *TTN* | non-synonymous SNV |
| 2 | 179614469 | C | T | 1 | 1 | *TTN* | non-synonymous SNV |
| 3 | 41266103 | G | C | 1 | 1 | *CTNNB1* | non-synonymous SNV |
| 3 | 178919185 | G | C | 1 | 1 | *PIK3CA* | non-synonymous SNV |
| 3 | 189526072 | G | A | 1 | 1 | *TP63* | synonymous SNV |
| 4 | 48147247 | A | T | 1 | 1 | *TEC* | non-synonymous SNV |
| 4 | 84348865 | C | T | 1 | 1 | *HELQ* | non-synonymous SNV |
| 4 | 99363232 | C | T | 1 | 1 | *RAP1GDS1* | synonymous SNV |
| 4 | 154625069 | G | A | 1 | 1 | *TLR2* | non-synonymous SNV |
| 4 | 166978381 | G | A | 1 | 1 | *TLL1* | non-synonymous SNV |
| 5 | 129030518 | G | A | 1 | 1 | *ADAMTS19* | non-synonymous SNV |
| 5 | 179200908 | C | T | 1 | 1 | *MAML1* | non-synonymous SNV |
| 6 | 30858771 | C | T | 1 | 1 | *DDR1* | non-synonymous SNV |
| 6 | 70894616 | A | C | 1 | 1 | *COL19A1* | non-synonymous SNV |
| 6 | 70916901 | G | A | 1 | 1 | *COL19A1* | non-synonymous SNV |
| 6 | 139242215 | T | A | 1 | 1 | *REPS1* | non-synonymous SNV |
| 7 | 6196540 | C | T | 1 | 1 | *USP42* | non-synonymous SNV |
| 7 | 43484010 | C | T | 1 | 1 | *HECW1* | synonymous SNV |
| 7 | 116395415 | C | T | 1 | 1 | *MET* | non-synonymous SNV |
| 7 | 140453136 | A | T | 1 | 1 | *BRAF* | non-synonymous SNV |
| 8 | 41575146 | C | T | 1 | 1 | *ANK1* | synonymous SNV |
| 9 | 35697911 | G | A | 1 | 1 | *TLN1* | synonymous SNV |
| 9 | 120475831 | C | T | 1 | 1 | *TLR4* | synonymous SNV |
| 9 | 131378006 | C | T | 1 | 1 | *SPTAN1* | synonymous SNV |
| 9 | 133759537 | C | T | 1 | 1 | *ABL1* | synonymous SNV |
| 9 | 139804437 | C | T | 1 | 1 | *TRAF2* | synonymous SNV |
| 10 | 8115751 | G | A | 1 | 1 | *GATA3* | non-synonymous SNV |
| 10 | 37478474 | G | A | 1 | 1 | *ANKRD30A* | non-synonymous SNV |
| 10 | 70405309 | C | T | 1 | 1 | *TET1* | synonymous SNV |
| 10 | 123298174 | C | T | 1 | 1 | *FGFR2* | non-synonymous SNV |
| 10 | 124380773 | G | A | 1 | 1 | *DMBT1* | non-synonymous SNV |
| 11 | 62592568 | G | A | 1 | 1 | *STX5* | non-synonymous SNV |
| 11 | 113670104 | G | A | 1 | 1 | *USP28* | non-synonymous SNV |
| 11 | 123516315 | C | T | 1 | 1 | *SCN3B* | non-synonymous SNV |
| 12 | 49426450 | C | T | 1 | 1 | *MLL2* | non-synonymous SNV |
| 12 | 121426792 | C | T | 1 | 1 | *HNF1A* | synonymous SNV |
| 13 | 21563077 | GG | AA | 1 | 1 | *LATS2* | non-frameshift substitution |
| 13 | 28578234 | C | T | 1 | 1 | *FLT3* | non-synonymous SNV |
| 13 | 99047680 | T | - | 1 | 1 | *FARP1* | frameshift deletion |
| 14 | 72128143 | C | T | 1 | 1 | *SIPA1L1* | synonymous SNV |
| 16 | 2258326 | C | T | 1 | 1 | *MLST8* | non-synonymous SNV |
| 17 | 10296449 | GG | TA | 1 | 1 | *MYH8* | non-frameshift substitution |
| 17 | 18144967 | C | T | 1 | 1 | *LLGL1* | non-synonymous SNV |
| 17 | 29592357 | G | A | 1 | 1 | *NF1* | non-synonymous SNV |
| 17 | 37676258 | C | T | 1 | 1 | *CDK12* | non-synonymous SNV |
| 17 | 78264373 | C | T | 1 | 1 | *RNF213* | synonymous SNV |
| 18 | 59815518 | T | C | 1 | 1 | *PIGN* | synonymous SNV |
| 19 | 9070975 | C | T | 1 | 1 | *MUC16* | non-synonymous SNV |
| 19 | 39116686 | C | T | 1 | 1 | *EIF3K* | stopgain SNV |
| 20 | 43052848 | C | T | 1 | 1 | *HNF4A* | synonymous SNV |
| 20 | 50782548 | A | T | 1 | 1 | *ZFP64* | non-synonymous SNV |
| 22 | 40417461 | C | T | 1 | 1 | *FAM83F* | non-synonymous SNV |
| X | 12906207 | T | C | 1 | 1 | *TLR7* | synonymous SNV |
| X | 17768126 | CC | TT | 1 | 1 | *SCML1* | non-frameshift substitution |
| X | 37300108 | C | T | 1 | 1 | *PRRG1* | non-synonymous SNV |
| X | 53600802 | G | A | 1 | 1 | *HUWE1* | non-synonymous SNV |
| 2 | 179407013 | G | A | 1 | 0 | *TTN* | synonymous SNV |
| 17 | 10419786 | C | T | 1 | 0 | *MYH1* | synonymous SNV |
| 1 | 44079338 | C | T | 0 | 1 | *PTPRF* | synonymous SNV |
| 2 | 97463346 | C | T | 0 | 1 | *CNNM4* | non-synonymous SNV |
| 2 | 179486277 | C | T | 0 | 1 | *TTN* | non-synonymous SNV |
| 2 | 234398081 | G | T | 0 | 1 | *USP40* | non-synonymous SNV |
| 22 | 37499267 | C | A | 0 | 1 | *TMPRSS6* | non-synonymous SNV |
