# Supplementary material for: Molecular and genetic diversity in the metastatic process of melanoma
Source: J Pathol. 2014 Jan 27;233(1):39–50. doi: 10.1002/path.4318 (PMC4359751; doi:10.1002/path.4318)
Supplement: Supplementary file 14 — Coding sequence (exonic and splice site) mutations in patients 1–28 [file path0233-0039-sd14.zip › TableS22.doc]

**Table S22.** Exonic and splice site mutations in patient 25

| **Chrom** | **Position** | **Reference** | **Alternative** | **M1** | **M2** | **Gene** | **Type** |
| --- | --- | --- | --- | --- | --- | --- | --- |
| 1 | 46501429 | C | T | 1 | 1 | *MAST2* | synonymous SNV |
| 1 | 115256528 | T | A | 1 | 1 | *NRAS* | non-synonymous SNV |
| 1 | 115527390 | A | T | 1 | 1 | *SYCP1* | non-synonymous SNV |
| 1 | 156845395 | G | A | 1 | 1 | *NTRK1* | non-synonymous SNV |
| 1 | 196973850 | C | T | 1 | 1 | *CFHR5* | non-synonymous SNV |
| 1 | 204401370 | G | A | 1 | 1 | *PIK3C2B* | synonymous SNV |
| 1 | 227221049 | G | A | 1 | 1 | *CDC42BPA* | non-synonymous SNV |
| 2 | 48915222 | C | T | 1 | 1 | *LHCGR* | non-synonymous SNV |
| 2 | 60688966 | G | A | 1 | 1 | *BCL11A* | non-synonymous SNV |
| 2 | 60695908 | G | A | 1 | 1 | *BCL11A* | non-synonymous SNV |
| 2 | 179473420 | C | T | 1 | 1 | *TTN* | non-synonymous SNV |
| 2 | 179597342 | T | A | 1 | 1 | *TTN* | non-synonymous SNV |
| 2 | 179616548 | A | T | 1 | 1 | *TTN* | non-synonymous SNV |
| 2 | 179638304 | G | A | 1 | 1 | *TTN* | synonymous SNV |
| 2 | 210559605 | T | A | 1 | 1 | *MAP2* | non-synonymous SNV |
| 2 | 212989600 | G | A | 1 | 1 | *ERBB4* | synonymous SNV |
| 2 | 234429672 | C | T | 1 | 1 | *USP40* | non-synonymous SNV |
| 3 | 1371584 | G | A | 1 | 1 | *CNTN6* | stopgain SNV |
| 3 | 9100075 | G | C | 1 | 1 | *SRGAP3* | non-synonymous SNV |
| 3 | 48613693 | C | T | 1 | 1 | *COL7A1* | non-synonymous SNV |
| 3 | 71008446 | G | A | 1 | 1 | *FOXP1* | synonymous SNV |
| 3 | 96963003 | C | T | 1 | 1 | *EPHA6* | non-synonymous SNV |
| 3 | 121212421 | A | G | 1 | 1 | *POLQ* | non-synonymous SNV |
| 3 | 140406642 | G | A | 1 | 1 | *TRIM42* | non-synonymous SNV |
| 3 | 142238554 | G | A | 1 | 1 | *ATR* | non-synonymous SNV |
| 3 | 160786793 | G | A | 1 | 1 | *PPM1L* | non-synonymous SNV |
| 3 | 186006598 | G | A | 1 | 1 | *DGKG* | non-synonymous SNV |
| 3 | 189526072 | G | A | 1 | 1 | *TP63* | synonymous SNV |
| 3 | 189584509 | G | A | 1 | 1 | *TP63* | non-synonymous SNV |
| 4 | 189060763 | C | T | 1 | 1 | *TRIML1* | synonymous SNV |
| 5 | 13883089 | G | A | 1 | 1 | *DNAH5* | non-synonymous SNV |
| 5 | 13891085 | C | T | 1 | 1 | *DNAH5* | synonymous SNV |
| 5 | 14497965 | C | T | 1 | 1 | *TRIO* | non-synonymous SNV |
| 5 | 112175087 | G | A | 1 | 1 | *APC* | non-synonymous SNV |
| 6 | 27834943 | T | C | 1 | 1 | *HIST1H1B* | non-synonymous SNV |
| 6 | 102266332 | C | T | 1 | 1 | *GRIK2* | non-synonymous SNV |
| 6 | 102337692 | C | T | 1 | 1 | *GRIK2* | synonymous SNV |
| 7 | 14613924 | C | T | 1 | 1 | *DGKB* | stopgain SNV |
| 7 | 18875586 | C | T | 1 | 1 | *HDAC9* | non-synonymous SNV |
| 7 | 37907430 | G | A | 1 | 1 | *TXNDC3* | non-synonymous SNV |
| 7 | 126173561 | G | A | 1 | 1 | *GRM8* | synonymous SNV |
| 7 | 142567618 | G | A | 1 | 1 | *EPHB6* | non-synonymous SNV |
| 7 | 143043254 | C | T | 1 | 1 | *CLCN1* | non-synonymous SNV |
| 7 | 157985130 | G | A | 1 | 1 | *PTPRN2* | synonymous SNV |
| 8 | 41577344 | C | A | 1 | 1 | *ANK1* | non-synonymous SNV |
| 8 | 100880603 | G | A | 1 | 1 | *VPS13B* | non-synonymous SNV |
| 8 | 113277689 | C | T | 1 | 1 | *CSMD3* | synonymous SNV |
| 8 | 113569157 | G | A | 1 | 1 | *CSMD3* | non-synonymous SNV |
| 9 | 21974543 | C | T | 1 | 1 | *CDKN2A* | non-synonymous SNV |
| 9 | 37711367 | C | T | 1 | 1 | *FRMPD1* | non-synonymous SNV |
| 9 | 96062351 | C | T | 1 | 1 | *WNK2* | non-synonymous SNV |
| 9 | 119858377 | C | T | 1 | 1 | *ASTN2* | non-synonymous SNV |
| 9 | 120476417 | G | A | 1 | 1 | *TLR4* | non-synonymous SNV |
| 10 | 75279693 | A | G | 1 | 1 | *USP54* | non-synonymous SNV |
| 10 | 104175860 | G | A | 1 | 1 | *PSD* | non-synonymous SNV |
| 10 | 129868638 | C | T | 1 | 1 | *PTPRE* | non-synonymous SNV |
| 11 | 77633484 | G | C | 1 | 1 | *INTS4* | synonymous SNV |
| 12 | 7947076 | G | A | 1 | 1 | *NANOG* | synonymous SNV |
| 12 | 49431162 | G | A | 1 | 1 | *MLL2* | non-synonymous SNV |
| 12 | 49445529 | G | C | 1 | 1 | *MLL2* | non-synonymous SNV |
| 12 | 53588017 | G | A | 1 | 1 | *ITGB7* | stopgain SNV |
| 12 | 57573191 | C | T | 1 | 1 | *LRP1* | synonymous SNV |
| 12 | 81046543 | G | A | 1 | 1 | *PTPRQ* | synonymous SNV |
| 12 | 108686185 | C | T | 1 | 1 | *CMKLR1* | synonymous SNV |
| 13 | 28578200 | C | T | 1 | 1 | *FLT3* | non-synonymous SNV |
| 13 | 28602354 | C | T | 1 | 1 | *FLT3* | non-synonymous SNV |
| 13 | 28903792 | C | T | 1 | 1 | *FLT1* | synonymous SNV |
| 14 | 96707082 | G | A | 1 | 1 | *BDKRB2* | non-synonymous SNV |
| 15 | 50833434 | C | T | 1 | 1 | *USP50* | non-synonymous SNV |
| 16 | 47727316 | T | A | 1 | 1 | *PHKB* | non-synonymous SNV |
| 17 | 10415478 | C | T | 1 | 1 | *MYH1* | synonymous SNV |
| 17 | 40500429 | G | A | 1 | 1 | *STAT3* | non-synonymous SNV |
| 17 | 45452107 | C | G | 1 | 1 | *C17orf57* | non-synonymous SNV |
| 18 | 59221597 | G | A | 1 | 1 | *CDH20* | non-synonymous SNV |
| 19 | 8998761 | G | A | 1 | 1 | *MUC16* | non-synonymous SNV |
| 19 | 9059142 | G | A | 1 | 1 | *MUC16* | non-synonymous SNV |
| 19 | 9066977 | G | A | 1 | 1 | *MUC16* | synonymous SNV |
| 19 | 9090961 | G | A | 1 | 1 | *MUC16* | non-synonymous SNV |
| 19 | 18273796 | C | T | 1 | 1 | *PIK3R2* | synonymous SNV |
| 19 | 48919280 | A | C | 1 | 1 | *GRIN2D* | non-synonymous SNV |
| 19 | 56459312 | C | T | 1 | 1 | *NLRP8* | non-synonymous SNV |
| 19 | 56499245 | G | A | 1 | 1 | *NLRP8* | non-synonymous SNV |
| 19 | 57642077 | C | T | 1 | 1 | *USP29* | synonymous SNV |
| 20 | 23669483 | C | T | 1 | 1 | *CST4* | non-synonymous SNV |
| 20 | 40730801 | C | T | 1 | 1 | *PTPRT* | non-synonymous SNV |
| 20 | 40770576 | G | A | 1 | 1 | *PTPRT* | stopgain SNV |
| 20 | 43052976 | G | A | 1 | 1 | *HNF4A* | non-synonymous SNV |
| 21 | 32554793 | C | T | 1 | 1 | *TIAM1* | synonymous SNV |
| 21 | 44590729 | G | A | 1 | 1 | *CRYAA* | non-synonymous SNV |
| 21 | 46306783 | G | A | 1 | 1 | *ITGB2* | synonymous SNV |
| X | 123870978 | G | A | 1 | 1 | *ODZ1* | non-synonymous SNV |
| X | 129147805 | C | T | 1 | 1 | *BCORL1* | non-synonymous SNV |
| 2 | 170103316 | G | A | 1 | 0 | *LRP2* | non-synonymous SNV |
| 2 | 179474266 | T | C | 1 | 0 | *TTN* | synonymous SNV |
| 2 | 179605631 | C | T | 1 | 0 | *TTN* | non-synonymous SNV |
| 5 | 24488146 | C | T | 1 | 0 | *CDH10* | non-synonymous SNV |
| 7 | 99769758 | C | T | 1 | 0 | *GPC2* | synonymous SNV |
| 10 | 26310470 | C | T | 1 | 0 | *MYO3A* | synonymous SNV |
| 17 | 10307758 | G | A | 1 | 0 | *MYH8* | synonymous SNV |
| 17 | 10405213 | C | T | 1 | 0 | *MYH1* | non-synonymous SNV |
| 17 | 11660930 | G | A | 1 | 0 | *DNAH9* | non-synonymous SNV |
| 18 | 51690931 | G | A | 1 | 0 | *MBD2* | synonymous SNV |
| 20 | 10644614 | C | G | 1 | 0 | *JAG1* | non-synonymous SNV |
| 1 | 204409432 | G | C | 0 | 1 | *PIK3C2B* | synonymous SNV |
| 2 | 54167105 | T | C | 0 | 1 | *PSME4* | non-synonymous SNV |
| 6 | 129513840 | G | A | 0 | 1 | *LAMA2* | non-synonymous SNV |
| 6 | 152861132 | C | T | 0 | 1 | *SYNE1* | non-synonymous SNV |
| 7 | 33644517 | A | T | 0 | 1 | *BBS9* | non-synonymous SNV |
| 12 | 56478874 | C | G | 0 | 1 | *ERBB3* | synonymous SNV |
| 12 | 80266599 | A | T | 0 | 1 | *PPP1R12A* | synonymous SNV |
| 16 | 2129160 | C | T | 0 | 1 | *TSC2* | stopgain SNV |
| 19 | 8808236 | C | T | 0 | 1 | *ACTL9* | synonymous SNV |
| 19 | 8971717 | C | T | 0 | 1 | *MUC16* | stopgain SNV |
| 19 | 9069205 | C | G | 0 | 1 | *MUC16* | non-synonymous SNV |
| X | 123515059 | T | G | 0 | 1 | *ODZ1* | non-synonymous SNV |
