# Supplementary material for: Molecular and genetic diversity in the metastatic process of melanoma
Source: J Pathol. 2014 Jan 27;233(1):39–50. doi: 10.1002/path.4318 (PMC4359751; doi:10.1002/path.4318)
Supplement: Supplementary file 14 — Coding sequence (exonic and splice site) mutations in patients 1–28 [file path0233-0039-sd14.zip › TableS23.doc]

**Table S23.** Exonic and splice site mutations in patient 26

| **Chrom** | **Position** | **Reference** | **Alternative** | **M1** | **M2** | **Gene** | **Type** |
| --- | --- | --- | --- | --- | --- | --- | --- |
| 1 | 33790594 | GG | AA | 1 | 1 | *PHC2* | non-frameshift substitution |
| 2 | 29420520 | C | T | 1 | 1 | *ALK* | non-synonymous SNV |
| 2 | 97464924 | C | T | 1 | 1 | *CNNM4* | synonymous SNV |
| 2 | 161159914 | G | A | 1 | 1 | *RBMS1* | non-synonymous SNV |
| 2 | 179588989 | G | A | 1 | 1 | *TTN* | non-synonymous SNV |
| 2 | 179616613 | G | A | 1 | 1 | *TTN* | non-synonymous SNV |
| 2 | 179638007 | C | T | 1 | 1 | *TTN* | non-synonymous SNV |
| 2 | 211438032 | CA | AC | 1 | 1 | *CPS1* | non-frameshift substitution |
| 2 | 212295702 | C | T | 1 | 1 | *ERBB4* | non-synonymous SNV |
| 3 | 36779796 | C | T | 1 | 1 | *DCLK3* | non-synonymous SNV |
| 3 | 96962884 | G | A | 1 | 1 | *EPHA6* | synonymous SNV |
| 3 | 108725917 | C | T | 1 | 1 | *MORC1* | non-synonymous SNV |
| 3 | 123987683 | G | A | 1 | 1 | *KALRN* | non-synonymous SNV |
| 3 | 189711991 | C | T | 1 | 1 | *LEPREL1* | non-synonymous SNV |
| 4 | 62903453 | G | A | 1 | 1 | *LPHN3* | non-synonymous SNV |
| 4 | 104640599 | G | A | 1 | 1 | *TACR3* | synonymous SNV |
| 5 | 13793786 | G | A | 1 | 1 | *DNAH5* | synonymous SNV |
| 5 | 13913992 | C | T | 1 | 1 | *DNAH5* | non-synonymous SNV |
| 5 | 178413186 | G | A | 1 | 1 | *GRM6* | non-synonymous SNV |
| 6 | 55128600 | C | T | 1 | 1 | *HCRTR2* | non-synonymous SNV |
| 6 | 73001666 | C | T | 1 | 1 | *RIMS1* | non-synonymous SNV |
| 6 | 79650521 | C | T | 1 | 1 | *PHIP* | synonymous SNV |
| 6 | 146350972 | G | A | 1 | 1 | *GRM1* | non-synonymous SNV |
| 7 | 140453136 | A | T | 1 | 1 | *BRAF* | non-synonymous SNV |
| 7 | 143028368 | C | T | 1 | 1 | *CLCN1* | synonymous SNV |
| 9 | 21971126 | G | C | 1 | 1 | *CDKN2A* | non-synonymous SNV |
| 9 | 32634481 | C | T | 1 | 1 | *TAF1L* | non-synonymous SNV |
| 9 | 86518550 | C | A | 1 | 1 | *KIF27* | non-synonymous SNV |
| 10 | 37482124 | G | A | 1 | 1 | *ANKRD30A* | non-synonymous SNV |
| 10 | 123844338 | C | T | 1 | 1 | *TACC2* | non-synonymous SNV |
| 11 | 7067935 | C | T | 1 | 1 | *NLRP14* | synonymous SNV |
| 11 | 119999125 | CC | TT | 1 | 1 | *TRIM29* | non-frameshift substitution |
| 12 | 4385251 | G | A | 1 | 1 | *CCND2* | synonymous SNV |
| 12 | 52374930 | C | T | 1 | 1 | *ACVR1B* | non-synonymous SNV |
| 12 | 53012094 | C | T | 1 | 1 | *KRT73* | non-synonymous SNV |
| 12 | 101711374 | T | C | 1 | 1 | *UTP20* | synonymous SNV |
| 12 | 133733181 | C | T | 1 | 1 | *ZNF10* | non-synonymous SNV |
| 13 | 61987471 | C | T | 1 | 1 | *PCDH20* | non-synonymous SNV |
| 14 | 64519861 | C | T | 1 | 1 | *SYNE2* | non-synonymous SNV |
| 14 | 102605694 | GG | AA | 1 | 1 | *HSP90AA1* | non-frameshift substitution |
| 16 | 56906280 | C | T | 1 | 1 | *SLC12A3* | synonymous SNV |
| 16 | 75276453 | G | A | 1 | 1 | *BCAR1* | non-synonymous SNV |
| 17 | 5042788 | GG | AA | 1 | 1 | *USP6* | non-frameshift substitution |
| 17 | 5066191 | C | T | 1 | 1 | *USP6* | synonymous SNV |
| 17 | 11520853 | C | T | 1 | 1 | *DNAH9* | synonymous SNV |
| 17 | 39913964 | C | T | 1 | 1 | *JUP* | non-synonymous SNV |
| 17 | 57076785 | C | T | 1 | 1 | *TRIM37* | non-synonymous SNV |
| 17 | 76799739 | C | T | 1 | 1 | *USP36* | synonymous SNV |
| 19 | 9048302 | C | T | 1 | 1 | *MUC16* | non-synonymous SNV |
| 19 | 9070602 | C | T | 1 | 1 | *MUC16* | non-synonymous SNV |
| 19 | 9073469 | G | A | 1 | 1 | *MUC16* | synonymous SNV |
| 19 | 9088666 | C | T | 1 | 1 | *MUC16* | non-synonymous SNV |
| 19 | 9089853 | TG | AT | 1 | 1 | *MUC16* | non-frameshift substitution |
| 19 | 9090411 | G | A | 1 | 1 | *MUC16* | synonymous SNV |
| 19 | 41175810 | G | A | 1 | 1 | *NUMBL* | synonymous SNV |
| 19 | 41736938 | C | A | 1 | 1 | *AXL* | non-synonymous SNV |
| 21 | 10952954 | A | G | 1 | 1 | *TPTE* | synonymous SNV |
| 22 | 28195264 | G | A | 1 | 1 | *MN1* | non-synonymous SNV |
| 22 | 37882053 | C | T | 1 | 1 | *MFNG* | non-synonymous SNV |
| X | 47485507 | A | C | 1 | 1 | *CFP* | non-synonymous SNV |
| X | 48674365 | C | T | 1 | 1 | *HDAC6* | non-synonymous SNV |
| X | 123517667 | C | T | 1 | 1 | *TENM1* | non-synonymous SNV |
| 17 | 54921539 | G | C | 1 | 1 | *DGKE* | synonymous SNV |
