# Supplementary material for: Molecular and genetic diversity in the metastatic process of melanoma
Source: J Pathol. 2014 Jan 27;233(1):39–50. doi: 10.1002/path.4318 (PMC4359751; doi:10.1002/path.4318)
Supplement: Supplementary file 14 — Coding sequence (exonic and splice site) mutations in patients 1–28 [file path0233-0039-sd14.zip › TableS24.doc]

**Table S24.** Exonic and splice site mutations in patient 27

| **Chrom** | **Position** | **Reference** | **Alternative** | **M1** | **M2** | **Gene** | **Type** |
| --- | --- | --- | --- | --- | --- | --- | --- |
| 1 | 19449503 | G | A | 1 | 1 | *UBR4* | synonymous SNV |
| 1 | 19493523 | G | A | 1 | 1 | *UBR4* | non-synonymous SNV |
| 1 | 29642660 | C | T | 1 | 1 | *PTPRU* | synonymous SNV |
| 1 | 38184470 | C | G | 1 | 1 | *EPHA10* | synonymous SNV |
| 1 | 39854191 | C | T | 1 | 1 | *MACF1* | non-synonymous SNV |
| 1 | 45289052 | G | A | 1 | 1 | *PTCH2* | synonymous SNV |
| 1 | 64644006 | C | T | 1 | 1 | *ROR1* | non-synonymous SNV |
| 1 | 82408826 | G | A | 1 | 1 | *LPHN2* | non-synonymous SNV |
| 1 | 114380963 | G | A | 1 | 1 | *PTPN22* | synonymous SNV |
| 1 | 115256529 | T | A | 1 | 1 | *NRAS* | non-synonymous SNV |
| 1 | 153659173 | G | A | 1 | 1 | *NPR1* | non-synonymous SNV |
| 1 | 155257895 | C | T | 1 | 1 | *HCN3* | non-synonymous SNV |
| 1 | 156756844 | C | T | 1 | 1 | *PRCC* | non-synonymous SNV |
| 1 | 157668362 | C | T | 1 | 1 | *FCRL3* | non-synonymous SNV |
| 1 | 179858482 | C | T | 1 | 1 | *TOR1AIP1* | synonymous SNV |
| 1 | 196645170 | G | A | 1 | 1 | *CFH* | stopgain SNV |
| 1 | 205277804 | G | A | 1 | 1 | *NUAK2* | non-synonymous SNV |
| 1 | 228553160 | G | A | 1 | 1 | *OBSCN* | non-synonymous SNV |
| 2 | 31595158 | G | A | 1 | 1 | *XDH* | non-synonymous SNV |
| 2 | 71766320 | C | T | 1 | 1 | *DYSF* | synonymous SNV |
| 2 | 102626143 | C | T | 1 | 1 | *IL1R2* | non-synonymous SNV |
| 2 | 103322324 | G | A | 1 | 1 | *SLC9A2* | non-synonymous SNV |
| 2 | 128036874 | G | A | 1 | 1 | *ERCC3* | synonymous SNV |
| 2 | 169801459 | G | A | 1 | 1 | *ABCB11* | non-synonymous SNV |
| 2 | 179396557 | C | T | 1 | 1 | *TTN* | non-synonymous SNV |
| 2 | 179418009 | C | T | 1 | 1 | *TTN* | stopgain SNV |
| 2 | 179577259 | G | A | 1 | 1 | *TTN* | synonymous SNV |
| 2 | 179593276 | C | T | 1 | 1 | *TTN* | synonymous SNV |
| 2 | 179594138 | C | T | 1 | 1 | *TTN* | non-synonymous SNV |
| 2 | 179595882 | C | T | 1 | 1 | *TTN* | non-synonymous SNV |
| 2 | 179600389 | G | A | 1 | 1 | *TTN* | synonymous SNV |
| 2 | 238280335 | G | A | 1 | 1 | *COL6A3* | non-synonymous SNV |
| 3 | 430998 | G | T | 1 | 1 | *CHL1* | stopgain SNV |
| 3 | 3084769 | C | T | 1 | 1 | *CNTN4* | non-synonymous SNV |
| 3 | 4726863 | G | A | 1 | 1 | *ITPR1* | synonymous SNV |
| 3 | 38138123 | C | T | 1 | 1 | *DLEC1* | synonymous SNV |
| 3 | 47061292 | G | A | 1 | 1 | *SETD2* | synonymous SNV |
| 3 | 59999745 | G | T | 1 | 1 | *FHIT* | synonymous SNV |
| 3 | 89448590 | G | A | 1 | 1 | *EPHA3* | synonymous SNV |
| 3 | 124196175 | C | T | 1 | 1 | *KALRN* | synonymous SNV |
| 3 | 124209704 | G | A | 1 | 1 | *KALRN* | synonymous SNV |
| 3 | 140997195 | C | T | 1 | 1 | *ACPL2* | non-synonymous SNV |
| 3 | 169846774 | G | A | 1 | 1 | *PHC3* | non-synonymous SNV |
| 3 | 187440353 | G | A | 1 | 1 | *BCL6* | stopgain SNV |
| 3 | 196612370 | C | T | 1 | 1 | *SENP5* | synonymous SNV |
| 4 | 46973232 | C | T | 1 | 1 | *GABRA4* | non-synonymous SNV |
| 4 | 87968244 | G | A | 1 | 1 | *AFF1* | non-synonymous SNV |
| 4 | 175897170 | G | A | 1 | 1 | *ADAM29* | non-synonymous SNV |
| 4 | 187525657 | T | C | 1 | 1 | *FAT1* | synonymous SNV |
| 4 | 187540516 | G | A | 1 | 1 | *FAT1* | synonymous SNV |
| 5 | 13700828 | C | T | 1 | 1 | *DNAH5* | synonymous SNV |
| 5 | 13753541 | C | T | 1 | 1 | *DNAH5* | non-synonymous SNV |
| 5 | 13807782 | C | T | 1 | 1 | *DNAH5* | non-synonymous SNV |
| 5 | 13883029 | C | T | 1 | 1 | *DNAH5* | non-synonymous SNV |
| 5 | 13920616 | C | T | 1 | 1 | *DNAH5* | non-synonymous SNV |
| 5 | 13928259 | A | G | 1 | 1 | *DNAH5* | non-synonymous SNV |
| 5 | 37008186 | C | A | 1 | 1 | *NIPBL* | non-synonymous SNV |
| 5 | 149212426 | C | T | 1 | 1 | *PPARGC1B* | non-synonymous SNV |
| 5 | 149216095 | G | T | 1 | 1 | *PPARGC1B* | non-synonymous SNV |
| 6 | 17675188 | C | T | 1 | 1 | *NUP153* | non-synonymous SNV |
| 6 | 31594835 | G | A | 1 | 1 | *PRRC2A* | non-synonymous SNV |
| 6 | 33263940 | G | A | 1 | 1 | *RGL2* | synonymous SNV |
| 6 | 51909843 | G | A | 1 | 1 | *PKHD1* | non-synonymous SNV |
| 6 | 69666549 | G | A | 1 | 1 | *BAI3* | stopgain SNV |
| 6 | 70070902 | C | T | 1 | 1 | *BAI3* | non-synonymous SNV |
| 6 | 70637823 | C | T | 1 | 1 | *COL19A1* | non-synonymous SNV |
| 6 | 70639391 | C | G | 1 | 1 | *COL19A1* | non-synonymous SNV |
| 6 | 70733549 | C | G | 1 | 1 | *COL19A1* | non-synonymous SNV |
| 6 | 84573982 | T | G | 1 | 1 | *CYB5R4* | non-synonymous SNV |
| 6 | 102483310 | G | A | 1 | 1 | *GRIK2* | non-synonymous SNV |
| 6 | 129465116 | C | T | 1 | 1 | *LAMA2* | non-synonymous SNV |
| 6 | 152469257 | G | A | 1 | 1 | *SYNE1* | non-synonymous SNV |
| 6 | 152470705 | C | T | 1 | 1 | *SYNE1* | synonymous SNV |
| 6 | 157527345 | C | - | 1 | 1 | *ARID1B* | frameshift deletion |
| 6 | 157527348 | G | T | 1 | 1 | *ARID1B* | synonymous SNV |
| 7 | 6027117 | G | A | 1 | 1 | *PMS2* | non-synonymous SNV |
| 7 | 18687413 | G | A | 1 | 1 | *HDAC9* | synonymous SNV |
| 7 | 47872702 | G | A | 1 | 1 | *PKD1L1* | non-synonymous SNV |
| 7 | 107871584 | C | T | 1 | 1 | *NRCAM* | stopgain SNV |
| 7 | 126086239 | C | T | 1 | 1 | *GRM8* | non-synonymous SNV |
| 7 | 126173270 | G | A | 1 | 1 | *GRM8* | synonymous SNV |
| 7 | 142563718 | C | T | 1 | 1 | *EPHB6* | non-synonymous SNV |
| 7 | 157449142 | C | T | 1 | 1 | *PTPRN2* | synonymous SNV |
| 8 | 41552743 | G | A | 1 | 1 | *ANK1* | non-synonymous SNV |
| 8 | 71041035 | G | A | 1 | 1 | *NCOA2* | non-synonymous SNV |
| 8 | 92999164 | T | A | 1 | 1 | *RUNX1T1* | non-synonymous SNV |
| 8 | 113988098 | C | T | 1 | 1 | *CSMD3* | non-synonymous SNV |
| 8 | 142441108 | CC | TT | 1 | 1 | *PTP4A3* | non-frameshift substitution |
| 9 | 27183479 | G | A | 1 | 1 | *TEK* | synonymous SNV |
| 9 | 32632328 | C | T | 1 | 1 | *TAF1L* | non-synonymous SNV |
| 9 | 35704360 | G | A | 1 | 1 | *TLN1* | non-synonymous SNV |
| 9 | 37745031 | C | T | 1 | 1 | *FRMPD1* | non-synonymous SNV |
| 9 | 78638706 | C | A | 1 | 1 | *PCSK5* | non-synonymous SNV |
| 9 | 120476125 | C | T | 1 | 1 | *TLR4* | synonymous SNV |
| 9 | 120476203 | G | A | 1 | 1 | *TLR4* | synonymous SNV |
| 9 | 133759661 | C | T | 1 | 1 | *ABL1* | non-synonymous SNV |
| 10 | 13534825 | C | T | 1 | 1 | *BEND7* | non-synonymous SNV |
| 10 | 37431026 | G | A | 1 | 1 | *ANKRD30A* | non-synonymous SNV |
| 10 | 37476934 | C | T | 1 | 1 | *ANKRD30A* | non-synonymous SNV |
| 10 | 81036966 | C | T | 1 | 1 | *ZMIZ1* | synonymous SNV |
| 10 | 84745277 | C | T | 1 | 1 | *NRG3* | synonymous SNV |
| 10 | 104173861 | G | A | 1 | 1 | *PSD* | synonymous SNV |
| 10 | 124008594 | G | A | 1 | 1 | *TACC2* | synonymous SNV |
| 11 | 22396420 | C | T | 1 | 1 | *SLC17A6* | synonymous SNV |
| 11 | 77594932 | G | A | 1 | 1 | *INTS4* | non-synonymous SNV |
| 11 | 77602401 | C | G | 1 | 1 | *INTS4* | non-synonymous SNV |
| 11 | 113269888 | C | T | 1 | 1 | *ANKK1* | synonymous SNV |
| 11 | 120957608 | C | T | 1 | 1 | *TBCEL* | non-synonymous SNV |
| 11 | 121475894 | G | A | 1 | 1 | *SORL1* | stopgain SNV |
| 11 | 121476167 | C | T | 1 | 1 | *SORL1* | non-synonymous SNV |
| 11 | 121477609 | G | A | 1 | 1 | *SORL1* | synonymous SNV |
| 12 | 4554589 | C | T | 1 | 1 | *FGF6* | non-synonymous SNV |
| 12 | 18800840 | G | A | 1 | 1 | *PIK3C2G* | non-synonymous SNV |
| 12 | 41318372 | CC | TT | 1 | 1 | *CNTN1* | non-frameshift substitution |
| 12 | 57588418 | C | T | 1 | 1 | *LRP1* | synonymous SNV |
| 12 | 70932732 | C | T | 1 | 1 | *PTPRB* | synonymous SNV |
| 12 | 70960368 | C | T | 1 | 1 | *PTPRB* | non-synonymous SNV |
| 12 | 70964935 | G | A | 1 | 1 | *PTPRB* | non-synonymous SNV |
| 12 | 70988447 | G | A | 1 | 1 | *PTPRB* | non-synonymous SNV |
| 12 | 112096610 | C | T | 1 | 1 | *BRAP* | non-synonymous SNV |
| 12 | 120152037 | G | A | 1 | 1 | *CIT* | non-synonymous SNV |
| 13 | 27847421 | C | T | 1 | 1 | *RASL11A* | synonymous SNV |
| 13 | 28880836 | G | A | 1 | 1 | *FLT1* | non-synonymous SNV |
| 13 | 38211694 | G | A | 1 | 1 | *TRPC4* | synonymous SNV |
| 14 | 39716274 | T | C | 1 | 1 | *MIA2* | non-synonymous SNV |
| 14 | 39717175 | A | G | 1 | 1 | *MIA2* | non-synonymous SNV |
| 14 | 39815159 | G | T | 1 | 1 | *CTAGE5* | non-synonymous SNV |
| 14 | 50625329 | G | A | 1 | 1 | *SOS2* | non-synonymous SNV |
| 15 | 63579687 | C | T | 1 | 1 | *APH1B* | synonymous SNV |
| 15 | 74336761 | C | T | 1 | 1 | *PML* | synonymous SNV |
| 16 | 19884067 | G | A | 1 | 1 | *GPRC5B* | non-synonymous SNV |
| 16 | 31075492 | G | A | 1 | 1 | *ZNF668* | non-synonymous SNV |
| 16 | 50783766 | C | T | 1 | 1 | *CYLD* | non-synonymous SNV |
| 16 | 61689619 | G | A | 1 | 1 | *CDH8* | non-synonymous SNV |
| 16 | 61859037 | C | T | 1 | 1 | *CDH8* | synonymous SNV |
| 16 | 62055176 | C | T | 1 | 1 | *CDH8* | synonymous SNV |
| 16 | 77398191 | G | A | 1 | 1 | *ADAMTS18* | non-synonymous SNV |
| 17 | 5073859 | G | A | 1 | 1 | *USP6* | synonymous SNV |
| 17 | 7577559 | G | A | 1 | 1 | *TP53* | non-synonymous SNV |
| 17 | 8052945 | G | A | 1 | 1 | *PER1* | stopgain SNV |
| 17 | 11687804 | G | A | 1 | 1 | *DNAH9* | non-synonymous SNV |
| 17 | 29663410 | C | T | 1 | 1 | *NF1* | synonymous SNV |
| 17 | 34195696 | G | A | 1 | 1 | *C17orf66* | synonymous SNV |
| 17 | 76802320 | G | A | 1 | 1 | *USP36* | non-synonymous SNV |
| 18 | 59157987 | C | T | 1 | 1 | *CDH20* | synonymous SNV |
| 19 | 6040050 | C | T | 1 | 1 | *RFX2* | non-synonymous SNV |
| 19 | 8807834 | C | T | 1 | 1 | *ACTL9* | synonymous SNV |
| 19 | 8966650 | C | T | 1 | 1 | *MUC16* | non-synonymous SNV |
| 19 | 9005722 | C | T | 1 | 1 | *MUC16* | synonymous SNV |
| 19 | 9047422 | C | T | 1 | 1 | *MUC16* | non-synonymous SNV |
| 19 | 9060108 | C | T | 1 | 1 | *MUC16* | non-synonymous SNV |
| 19 | 9062188 | C | T | 1 | 1 | *MUC16* | non-synonymous SNV |
| 19 | 9062992 | C | T | 1 | 1 | *MUC16* | non-synonymous SNV |
| 19 | 9069911 | G | A | 1 | 1 | *MUC16* | synonymous SNV |
| 19 | 9071577 | G | A | 1 | 1 | *MUC16* | non-synonymous SNV |
| 19 | 9074964 | G | A | 1 | 1 | *MUC16* | non-synonymous SNV |
| 19 | 19232457 | G | A | 1 | 1 | *TMEM161A* | non-synonymous SNV |
| 19 | 35802921 | G | A | 1 | 1 | *MAG* | splicing |
| 19 | 37905006 | G | A | 1 | 1 | *ZNF569* | non-synonymous SNV |
| 19 | 48908324 | G | A | 1 | 1 | *GRIN2D* | non-synonymous SNV |
| 19 | 55447752 | G | A | 1 | 1 | *NLRP7* | non-synonymous SNV |
| 19 | 56249612 | G | A | 1 | 1 | *NLRP9* | synonymous SNV |
| 20 | 10030446 | C | T | 1 | 1 | *ANKRD5* | non-synonymous SNV |
| 20 | 57876655 | G | A | 1 | 1 | *EDN3* | synonymous SNV |
| 21 | 10906969 | A | T | 1 | 1 | *TPTE* | non-synonymous SNV |
| 21 | 19647579 | C | T | 1 | 1 | *TMPRSS15* | non-synonymous SNV |
| 21 | 32638596 | G | A | 1 | 1 | *TIAM1* | synonymous SNV |
| 21 | 38525282 | C | T | 1 | 1 | *TTC3* | synonymous SNV |
| 21 | 39672098 | C | T | 1 | 1 | *KCNJ15* | synonymous SNV |
| 21 | 46032225 | CC | TT | 1 | 1 | *KRTAP10-8* | non-frameshift substitution |
| 22 | 37902324 | GG | AA | 1 | 1 | *CARD10* | non-frameshift substitution |
| X | 9900764 | G | T | 1 | 1 | *SHROOM2* | synonymous SNV |
| X | 20034397 | G | A | 1 | 1 | *MAP7D2* | non-synonymous SNV |
| X | 49067838 | C | T | 1 | 1 | *CACNA1F* | non-synonymous SNV |
| X | 70320898 | C | T | 1 | 1 | *FOXO4* | non-synonymous SNV |
| X | 108697009 | C | T | 1 | 1 | *GUCY2F* | non-synonymous SNV |
| X | 123654622 | C | T | 1 | 1 | *ODZ1* | non-synonymous SNV |
| X | 150912579 | G | A | 1 | 1 | *CNGA2* | non-synonymous SNV |
| 17 | 27963281 | G | A | 1 | 0 | *SSH2* | non-synonymous SNV |
| 19 | 1619110 | C | A | 1 | 0 | *TCF3* | non-synonymous SNV |
| 19 | 6760861 | G | A | 1 | 0 | *SH2D3A* | synonymous SNV |
| 19 | 6825413 | G | A | 1 | 0 | *VAV1* | non-synonymous SNV |
| 19 | 6854104 | G | A | 1 | 0 | *VAV1* | non-synonymous SNV |
| 19 | 9062196 | C | T | 1 | 0 | *MUC16* | non-synonymous SNV |
| 19 | 9085573 | G | A | 1 | 0 | *MUC16* | non-synonymous SNV |
| 19 | 10472626 | G | A | 1 | 0 | *TYK2* | synonymous SNV |
| 1 | 155252359 | C | A | 0 | 1 | *HCN3* | synonymous SNV |
