# Supplementary material for: Molecular and genetic diversity in the metastatic process of melanoma
Source: J Pathol. 2014 Jan 27;233(1):39–50. doi: 10.1002/path.4318 (PMC4359751; doi:10.1002/path.4318)
Supplement: Supplementary file 14 — Coding sequence (exonic and splice site) mutations in patients 1–28 [file path0233-0039-sd14.zip › TableS25.doc]

**Table S25.** Exonic and splice site mutations in patient 28

| **Chrom** | **Position** | **Reference** | **Alternative** | **M1** | **M2** | **Gene** | **Type** |
| --- | --- | --- | --- | --- | --- | --- | --- |
| 1 | 115256530 | G | T | 1 | 1 | *NRAS* | non-synonymous SNV |
| 2 | 152273151 | A | C | 1 | 1 | *RIF1* | synonymous SNV |
| 2 | 179498554 | C | T | 1 | 1 | *TTN* | synonymous SNV |
| 2 | 179634890 | C | T | 1 | 1 | *TTN* | synonymous SNV |
| 3 | 93598100 | G | A | 1 | 1 | *PROS1* | synonymous SNV |
| 3 | 148601459 | G | A | 1 | 1 | *CPA3* | non-synonymous SNV |
| 4 | 114277384 | C | T | 1 | 1 | *ANK2* | non-synonymous SNV |
| 6 | 31839288 | C | A | 1 | 1 | *SLC44A4* | non-synonymous SNV |
| 6 | 51882232 | G | A | 1 | 1 | *PKHD1* | non-synonymous SNV |
| 7 | 55249104 | A | T | 1 | 1 | *EGFR* | non-synonymous SNV |
| 7 | 142563735 | G | T | 1 | 1 | *EPHB6* | stopgain SNV |
| 8 | 68981348 | G | A | 1 | 1 | *PREX2* | non-synonymous SNV |
| 8 | 126017894 | C | T | 1 | 1 | *SQLE* | synonymous SNV |
| 11 | 22382468 | G | A | 1 | 1 | *SLC17A6* | non-synonymous SNV |
| 11 | 74911310 | C | T | 1 | 1 | *SLCO2B1* | synonymous SNV |
| 16 | 9892315 | C | T | 1 | 1 | *GRIN2A* | synonymous SNV |
| 16 | 69967886 | C | T | 1 | 1 | *WWP2* | non-synonymous SNV |
| 17 | 10301885 | C | T | 1 | 1 | *MYH8* | non-synonymous SNV |
| 17 | 37564136 | G | A | 1 | 1 | *MED1* | synonymous SNV |
| 19 | 10468498 | G | A | 1 | 1 | *TYK2* | non-synonymous SNV |
| 20 | 8862456 | G | A | 1 | 1 | *PLCB1* | non-synonymous SNV |
| 20 | 33345235 | G | A | 1 | 1 | *NCOA6* | non-synonymous SNV |
| 20 | 40790171 | GG | AA | 1 | 1 | *PTPRT* | non-frameshift substitution |
| 20 | 46294644 | G | A | 1 | 1 | *SULF2* | non-synonymous SNV |
| 21 | 46313346 | G | A | 1 | 1 | *ITGB2* | synonymous SNV |
| 22 | 46930151 | G | A | 1 | 1 | *CELSR1* | non-synonymous SNV |
| X | 70597030 | C | T | 1 | 1 | *TAF1* | stopgain SNV |
| 19 | 49259528 | G | A | 1 | 1 | *FGF21* | non-synonymous SNV |
| X | 44870208 | T | G | 1 | 1 | *KDM6A* | non-synonymous SNV |
| 7 | 157414064 | G | C | 1 | 1 | *PTPRN2* | synonymous SNV |
| 1 | 27691338 | G | T | 1 | 0 | *MAP3K6* | non-synonymous SNV |
