# Supplementary material for: Molecular and genetic diversity in the metastatic process of melanoma
Source: J Pathol. 2014 Jan 27;233(1):39–50. doi: 10.1002/path.4318 (PMC4359751; doi:10.1002/path.4318)
Supplement: Supplementary file 14 — Coding sequence (exonic and splice site) mutations in patients 1–28 [file path0233-0039-sd14.zip › TableS4.doc]

**Table S4.** Exonic and splice site mutations in patient 1

| **Chrom** | **Position** | **Reference** | **Alternative** | **M1** | **M2** | **M3** | **Gene** | **Type** |
| --- | --- | --- | --- | --- | --- | --- | --- | --- |
| 1 | 3624319 | C | T | 1 | 1 | 1 | *TP73* | synonymous SNV |
| 1 | 6304613 | G | A | 1 | 1 | 1 | *HES3* | synonymous SNV |
| 1 | 60309173 | C | T | 1 | 1 | 1 | *HOOK1* | stopgain SNV |
| 1 | 196946818 | C | T | 1 | 1 | 1 | *CFHR5* | synonymous SNV |
| 1 | 205272874 | G | A | 1 | 1 | 1 | *NUAK2* | non-synonymous SNV |
| 1 | 228473938 | C | T | 1 | 1 | 1 | *OBSCN* | non-synonymous SNV |
| 1 | 229606429 | G | A | 1 | 1 | 1 | *NUP133* | synonymous SNV |
| 2 | 179395988 | G | A | 1 | 1 | 1 | *TTN* | synonymous SNV |
| 2 | 179481726 | CC | TT | 1 | 1 | 1 | *TTN* | non-frameshift substitution |
| 2 | 220167375 | G | A | 1 | 1 | 1 | *PTPRN* | synonymous SNV |
| 2 | 238277457 | C | T | 1 | 1 | 1 | *COL6A3* | non-synonymous SNV |
| 3 | 37369100 | C | T | 1 | 1 | 1 | *GOLGA4* | non-synonymous SNV |
| 3 | 47098478 | G | A | 1 | 1 | 1 | *SETD2* | stopgain SNV |
| 3 | 62142742 | G | A | 1 | 1 | 1 | *PTPRG* | synonymous SNV |
| 3 | 121413198 | G | A | 1 | 1 | 1 | *GOLGB1* | stopgain SNV |
| 3 | 135741954 | A | C | 1 | 1 | 1 | *PPP2R3A* | synonymous SNV |
| 3 | 184293660 | C | T | 1 | 1 | 1 | *EPHB3* | non-synonymous SNV |
| 3 | 189582105 | G | A | 1 | 1 | 1 | *TP63* | non-synonymous SNV |
| 4 | 111469392 | G | A | 1 | 1 | 1 | *ENPEP* | synonymous SNV |
| 4 | 151773393 | G | A | 1 | 1 | 1 | *LRBA* | stopgain SNV |
| 5 | 1032303 | C | T | 1 | 1 | 1 | *NKD2* | non-synonymous SNV |
| 6 | 617718 | G | A | 1 | 1 | 1 | *EXOC2* | synonymous SNV |
| 6 | 73001677 | G | A | 1 | 1 | 1 | *RIMS1* | non-synonymous SNV |
| 6 | 117746713 | G | A | 1 | 1 | 1 | *ROS1* | non-synonymous SNV |
| 7 | 107566712 | C | T | 1 | 1 | 1 | *LAMB1* | synonymous SNV |
| 7 | 140453136 | A | T | 1 | 1 | 1 | *BRAF* | non-synonymous SNV |
| 7 | 143098631 | CC | TT | 1 | 1 | 1 | *EPHA1* | non-frameshift substitution |
| 8 | 32505607 | C | T | 1 | 1 | 1 | *NRG1* | non-synonymous SNV |
| 8 | 41571743 | G | A | 1 | 1 | 1 | *ANK1* | synonymous SNV |
| 8 | 42868471 | G | A | 1 | 1 | 1 | *HOOK3* | splicing |
| 8 | 114326879 | G | A | 1 | 1 | 1 | *CSMD3* | stopgain SNV |
| 9 | 27190599 | C | T | 1 | 1 | 1 | *TEK* | non-synonymous SNV |
| 10 | 17026250 | G | A | 1 | 1 | 1 | *CUBN* | non-synonymous SNV |
| 10 | 17171157 | T | A | 1 | 1 | 1 | *CUBN* | non-synonymous SNV |
| 10 | 123970197 | C | T | 1 | 1 | 1 | *TACC2* | non-synonymous SNV |
| 12 | 9075331 | C | T | 1 | 1 | 1 | *PHC1* | stopgain SNV |
| 12 | 44166831 | G | A | 1 | 1 | 1 | *IRAK4* | non-synonymous SNV |
| 12 | 81042697 | C | T | 1 | 1 | 1 | *PTPRQ* | synonymous SNV |
| 13 | 28589756 | C | T | 1 | 1 | 1 | *FLT3* | non-synonymous SNV |
| 13 | 48947540 | G | A | 1 | 1 | 1 | *RB1* | splicing |
| 13 | 48955431 | GG | AA | 1 | 1 | 1 | *RB1* | stopgain immediate |
| 14 | 94053106 | G | A | 1 | 1 | 1 | *UNC79* | non-synonymous SNV |
| 14 | 102717307 | C | T | 1 | 1 | 1 | *MOK* | synonymous SNV |
| 16 | 9857436 | C | T | 1 | 1 | 1 | *GRIN2A* | non-synonymous SNV |
| 16 | 23360097 | C | T | 1 | 1 | 1 | *SCNN1B* | synonymous SNV |
| 16 | 56904620 | CC | TT | 1 | 1 | 1 | *SLC12A3* | non-frameshift substitution |
| 17 | 54912174 | G | A | 1 | 1 | 1 | *DGKE* | synonymous SNV |
| 17 | 56688543 | T | C | 1 | 1 | 1 | *TEX14* | non-synonymous SNV |
| 17 | 66518976 | C | T | 1 | 1 | 1 | *PRKAR1A* | non-synonymous SNV |
| 18 | 20768871 | G | A | 1 | 1 | 1 | *CABLES1* | synonymous SNV |
| 18 | 59755998 | G | A | 1 | 1 | 1 | *PIGN* | synonymous SNV |
| 19 | 9075610 | G | A | 1 | 1 | 1 | *MUC16* | non-synonymous SNV |
| 19 | 9080494 | G | A | 1 | 1 | 1 | *MUC16* | synonymous SNV |
| 19 | 9089419 | C | T | 1 | 1 | 1 | *MUC16* | non-synonymous SNV |
| 20 | 10032354 | C | T | 1 | 1 | 1 | *ANKRD5* | non-synonymous SNV |
| 21 | 38309630 | G | A | 1 | 1 | 1 | *HLCS* | stopgain SNV |
| 22 | 36661223 | C | T | 1 | 1 | 1 | *APOL1* | non-synonymous SNV |
| X | 70443944 | G | A | 1 | 1 | 1 | *GJB1* | synonymous SNV |
| X | 76938966 | G | A | 1 | 1 | 1 | *ATRX* | synonymous SNV |
| X | 86888760 | C | T | 1 | 1 | 1 | *KLHL4* | non-synonymous SNV |
| X | 134291361 | G | A | 1 | 1 | 1 | *CXorf48* | non-synonymous SNV |
| X | 135428106 | C | T | 1 | 1 | 1 | *GPR112* | synonymous SNV |
| X | 135428106 | C | T | 1 | 1 | 1 | *GPR112* | synonymous SNV |
| 2 | 3469461 | C | T | 1 | 0 | 0 | *TRAPPC12* | synonymous SNV |
| 12 | 108145919 | A | G | 1 | 0 | 0 | *PRDM4* | synonymous SNV |
| 3 | 178952085 | A | G | 0 | 1 | 1 | *PIK3CA* | non-synonymous SNV |
| 22 | 37893072 | C | A | 0 | 1 | 1 | *CARD10* | non-synonymous SNV |
| 2 | 234429695 | T | - | 0 | 1 | 0 | *USP40* | frameshift deletion |
| 3 | 134670848 | C | T | 0 | 1 | 0 | *EPHB1* | synonymous SNV |
| 5 | 180048787 | C | T | 0 | 1 | 0 | *FLT4* | non-synonymous SNV |
| 6 | 51890078 | G | T | 0 | 1 | 0 | *PKHD1* | synonymous SNV |
| 9 | 35722872 | G | A | 0 | 1 | 0 | *TLN1* | non-synonymous SNV |
| 11 | 866628 | T | C | 0 | 1 | 0 | *TSPAN4* | stoploss SNV |
| 17 | 11837341 | C | T | 0 | 1 | 0 | *DNAH9* | non-synonymous SNV |
| 17 | 40843161 | A | G | 0 | 1 | 0 | *CNTNAP1* | non-synonymous SNV |
| 1 | 36230826 | A | G | 0 | 0 | 1 | *CLSPN* | synonymous SNV |
| 1 | 157512718 | T | A | 0 | 0 | 1 | *FCRL5* | non-synonymous SNV |
| 1 | 203276437 | C | T | 0 | 0 | 1 | *BTG2* | synonymous SNV |
| 2 | 47604208 | A | G | 0 | 0 | 1 | *EPCAM* | non-synonymous SNV |
| 2 | 179396825 | T | C | 0 | 0 | 1 | *TTN* | synonymous SNV |
| 3 | 65349285 | T | C | 0 | 0 | 1 | *MAGI1* | splicing |
| 3 | 121386340 | G | T | 0 | 0 | 1 | *GOLGB1* | synonymous SNV |
| 4 | 88035822 | A | - | 0 | 0 | 1 | *AFF1* | frameshift deletion |
| 4 | 103527815 | A | - | 0 | 0 | 1 | *NFKB1* | frameshift deletion |
| 5 | 131931452 | A | - | 0 | 0 | 1 | *RAD50* | frameshift deletion |
| 6 | 4031881 | A | - | 0 | 0 | 1 | *PRPF4B* | frameshift deletion |
| 6 | 10764770 | T | - | 0 | 0 | 1 | *MAK* | frameshift deletion |
| 6 | 27835188 | C | - | 0 | 0 | 1 | *HIST1H1B* | frameshift deletion |
| 6 | 31132595 | C | T | 0 | 0 | 1 | *POU5F1* | non-synonymous SNV |
| 6 | 39398873 | A | G | 0 | 0 | 1 | *KIF6* | synonymous SNV |
| 6 | 44217268 | A | G | 0 | 0 | 1 | *HSP90AB1* | non-synonymous SNV |
| 8 | 22868063 | T | C | 0 | 0 | 1 | *RHOBTB2* | non-synonymous SNV |
| 8 | 42227508 | T | C | 0 | 0 | 1 | *POLB* | splicing |
| 9 | 96000605 | T | C | 0 | 0 | 1 | *WNK2* | splicing |
| 9 | 132637222 | T | C | 0 | 0 | 1 | *USP20* | non-synonymous SNV |
| 10 | 89712018 | T | C | 0 | 0 | 1 | *PTEN* | splicing |
| 11 | 48157598 | T | C | 0 | 0 | 1 | *PTPRJ* | synonymous SNV |
| 11 | 68206084 | A | G | 0 | 0 | 1 | *LRP5* | non-synonymous SNV |
| 11 | 118347558 | A | G | 0 | 0 | 1 | *MLL* | synonymous SNV |
| 13 | 21555734 | A | G | 0 | 0 | 1 | *LATS2* | non-synonymous SNV |
| 13 | 28877332 | A | G | 0 | 0 | 1 | *FLT1* | non-synonymous SNV |
| 14 | 64589121 | A | G | 0 | 0 | 1 | *SYNE2* | synonymous SNV |
| 16 | 68853184 | T | C | 0 | 0 | 1 | *CDH1* | non-synonymous SNV |
| 17 | 10321964 | G | A | 0 | 0 | 1 | *MYH8* | non-synonymous SNV |
| 17 | 29679292 | A | G | 0 | 0 | 1 | *NF1* | non-synonymous SNV |
| 17 | 40459733 | T | C | 0 | 0 | 1 | *STAT5A* | non-synonymous SNV |
| 17 | 74328400 | A | G | 0 | 0 | 1 | *PRPSAP1* | non-synonymous SNV |
| 18 | 45372043 | T | C | 0 | 0 | 1 | *SMAD2* | non-synonymous SNV |
| 21 | 40190441 | A | G | 0 | 0 | 1 | *ETS2* | non-synonymous SNV |
| X | 12905677 | A | - | 0 | 0 | 1 | *TLR7* | frameshift deletion |
| X | 49074247 | G | C | 0 | 0 | 1 | *CACNA1F* | non-synonymous SNV |
| X | 105881111 | A | G | 0 | 0 | 1 | *CXorf57* | synonymous SNV |
