# Supplementary material for: Molecular and genetic diversity in the metastatic process of melanoma
Source: J Pathol. 2014 Jan 27;233(1):39–50. doi: 10.1002/path.4318 (PMC4359751; doi:10.1002/path.4318)
Supplement: Supplementary file 14 — Coding sequence (exonic and splice site) mutations in patients 1–28 [file path0233-0039-sd14.zip › TableS5.doc]

**Table S5.** Exonic and splice site mutations in patient 2

| **Chrom** | **Position** | **Reference** | **Alternative** | **M1** | **M2** | **Gene** | **Type** |
| --- | --- | --- | --- | --- | --- | --- | --- |
| 1 | 22923821 | C | T | 1 | 1 | *EPHA8* | synonymous SNV |
| 1 | 55118798 | C | T | 1 | 1 | *HEATR8* | non-synonymous SNV |
| 1 | 103488381 | C | T | 1 | 1 | *COL11A1* | non-synonymous SNV |
| 1 | 103544381 | T | C | 1 | 1 | *COL11A1* | synonymous SNV |
| 1 | 109716108 | C | T | 1 | 1 | *KIAA1324* | stopgain SNV |
| 1 | 154570342 | G | A | 1 | 1 | *ADAR* | synonymous SNV |
| 1 | 162745966 | G | A | 1 | 1 | *DDR2* | non-synonymous SNV |
| 2 | 3469460 | C | T | 1 | 1 | *TRAPPC12* | synonymous SNV |
| 2 | 73493732 | G | A | 1 | 1 | *FBXO41* | synonymous SNV |
| 2 | 170127546 | C | T | 1 | 1 | *LRP2* | non-synonymous SNV |
| 2 | 179410191 | C | T | 1 | 1 | *TTN* | synonymous SNV |
| 2 | 179481561 | C | T | 1 | 1 | *TTN* | non-synonymous SNV |
| 2 | 179605061 | C | T | 1 | 1 | *TTN* | non-synonymous SNV |
| 2 | 217315673 | C | T | 1 | 1 | *SMARCAL1* | synonymous SNV |
| 2 | 240055951 | G | A | 1 | 1 | *HDAC4* | synonymous SNV |
| 2 | 240085566 | T | G | 1 | 1 | *HDAC4* | non-synonymous SNV |
| 3 | 9871007 | C | T | 1 | 1 | *ARPC4-TTLL3,TTLL3* | synonymous SNV |
| 3 | 16633556 | C | T | 1 | 1 | *DAZL* | splicing |
| 3 | 38103825 | C | T | 1 | 1 | *DLEC1* | non-synonymous SNV |
| 3 | 41266137 | C | T | 1 | 1 | *CTNNB1* | non-synonymous SNV |
| 3 | 167747612 | C | T | 1 | 1 | *GOLIM4* | synonymous SNV |
| 3 | 189590676 | T | G | 1 | 1 | *TP63* | non-synonymous SNV |
| 4 | 2242576 | C | T | 1 | 1 | *HAUS3* | non-synonymous SNV |
| 4 | 146059607 | CC | TT | 1 | 1 | *OTUD4* | non-frameshift substitution |
| 4 | 187517763 | G | A | 1 | 1 | *FAT1* | non-synonymous SNV |
| 5 | 13792159 | A | C | 1 | 1 | *DNAH5* | non-synonymous SNV |
| 5 | 13841033 | CC | TT | 1 | 1 | *DNAH5* | non-frameshift substitution |
| 5 | 132270205 | G | A | 1 | 1 | *AFF4* | synonymous SNV |
| 5 | 149441067 | C | T | 1 | 1 | *CSF1R* | synonymous SNV |
| 5 | 156608039 | A | C | 1 | 1 | *ITK* | non-synonymous SNV |
| 6 | 50712947 | G | A | 1 | 1 | *TFAP2D* | non-synonymous SNV |
| 6 | 55216194 | C | T | 1 | 1 | *GFRAL* | non-synonymous SNV |
| 6 | 69666080 | G | A | 1 | 1 | *BAI3* | non-synonymous SNV |
| 7 | 27148340 | C | T | 1 | 1 | *HOXA3* | splicing |
| 7 | 140453136 | A | T | 1 | 1 | *BRAF* | non-synonymous SNV |
| 8 | 100403843 | T | G | 1 | 1 | *VPS13B* | non-synonymous SNV |
| 9 | 8341729 | C | T | 1 | 1 | *PTPRD* | synonymous SNV |
| 10 | 129847894 | C | T | 1 | 1 | *PTPRE* | synonymous SNV |
| 11 | 2156604 | G | A | 1 | 1 | *IGF2* | synonymous SNV |
| 11 | 88924546 | G | A | 1 | 1 | *TYR* | non-synonymous SNV |
| 11 | 95825383 | C | T | 1 | 1 | *MAML2* | synonymous SNV |
| 12 | 49425394 | A | C | 1 | 1 | *MLL2* | non-synonymous SNV |
| 12 | 101777002 | G | A | 1 | 1 | *UTP20* | non-synonymous SNV |
| 14 | 21897399 | C | T | 1 | 1 | *CHD8* | synonymous SNV |
| 16 | 2506927 | C | T | 1 | 1 | *CCNF* | non-synonymous SNV |
| 16 | 15841476 | C | T | 1 | 1 | *MYH11* | non-synonymous SNV |
| 16 | 77401601 | C | T | 1 | 1 | *ADAMTS18* | non-synonymous SNV |
| 17 | 17120396 | A | G | 1 | 1 | *FLCN* | non-synonymous SNV |
| 17 | 40451795 | A | G | 1 | 1 | *STAT5A* | non-synonymous SNV |
| 17 | 78261647 | G | C | 1 | 1 | *RNF213* | non-synonymous SNV |
| 18 | 55342102 | CC | TT | 1 | 1 | *ATP8B1* | non-frameshift substitution |
| 19 | 9049527 | A | C | 1 | 1 | *MUC16* | non-synonymous SNV |
| 20 | 30816212 | T | G | 1 | 1 | *POFUT1* | non-synonymous SNV |
| 22 | 36692979 | C | T | 1 | 1 | *MYH9* | non-synonymous SNV |
| 22 | 39626229 | C | T | 1 | 1 | *PDGFB* | non-synonymous SNV |
| X | 132887590 | C | T | 1 | 1 | *GPC3* | synonymous SNV |
| X | 135428953 | G | A | 1 | 1 | *GPR112* | non-synonymous SNV |
| X | 135428957 | T | G | 1 | 1 | *GPR112* | non-synonymous SNV |
| 1 | 228524772 | C | T | 1 | 0 | *OBSCN* | synonymous SNV |
| 5 | 139909232 | CC | TT | 1 | 0 | *ANKHD1,ANKHD1-EIF4EBP3* | non-frameshift substitution |
| 20 | 32878161 | C | T | 1 | 0 | *AHCY* | non-synonymous SNV |
| 21 | 36421194 | C | T | 1 | 0 | *RUNX1* | non-synonymous SNV |
| 10 | 127529455 | T | C | 0 | 1 | *DHX32* | non-synonymous SNV |
| 11 | 69625179 | C | T | 0 | 1 | *FGF3* | non-synonymous SNV |
| 11 | 121489571 | T | G | 0 | 1 | *SORL1* | non-synonymous SNV |
| 19 | 9060388 | T | C | 0 | 1 | *MUC16* | non-synonymous SNV |
