# Supplementary material for: Molecular and genetic diversity in the metastatic process of melanoma
Source: J Pathol. 2014 Jan 27;233(1):39–50. doi: 10.1002/path.4318 (PMC4359751; doi:10.1002/path.4318)
Supplement: Supplementary file 14 — Coding sequence (exonic and splice site) mutations in patients 1–28 [file path0233-0039-sd14.zip › TableS6.doc]

**Table S6.** Exonic and splice site mutations in patient 3

| **Chrom** | **Position** | **Reference** | **Alternative** | **M1** | **M2** | **Gene** | **Type** |
| --- | --- | --- | --- | --- | --- | --- | --- |
| 1 | 17592176 | G | A | 1 | 1 | *PADI3* | synonymous SNV |
| 1 | 22902808 | G | A | 1 | 1 | *EPHA8* | synonymous SNV |
| 1 | 36227602 | G | A | 1 | 1 | *CLSPN* | stopgain SNV |
| 1 | 70502205 | C | T | 1 | 1 | *LRRC7* | non-synonymous SNV |
| 1 | 103412427 | G | A | 1 | 1 | *COL11A1* | non-synonymous SNV |
| 1 | 113254974 | C | T | 1 | 1 | *PPM1J* | non-synonymous SNV |
| 1 | 196706776 | C | T | 1 | 1 | *CFH* | non-synonymous SNV |
| 1 | 200618239 | G | A | 1 | 1 | *DDX59* | non-synonymous SNV |
| 1 | 228444494 | C | T | 1 | 1 | *OBSCN* | synonymous SNV |
| 2 | 71883405 | G | A | 1 | 1 | *DYSF* | synonymous SNV |
| 2 | 97427536 | C | T | 1 | 1 | *CNNM4* | non-synonymous SNV |
| 2 | 103318920 | C | T | 1 | 1 | *SLC9A2* | stopgain SNV |
| 2 | 135779349 | G | A | 1 | 1 | *YSK4* | non-synonymous SNV |
| 2 | 170031804 | C | T | 1 | 1 | *LRP2* | non-synonymous SNV |
| 2 | 179437833 | G | A | 1 | 1 | *TTN* | synonymous SNV |
| 2 | 179588340 | T | - | 1 | 1 | *TTN* | frameshift deletion |
| 2 | 179588856 | G | A | 1 | 1 | *TTN* | non-synonymous SNV |
| 2 | 179650370 | G | A | 1 | 1 | *TTN* | non-synonymous SNV |
| 2 | 236706487 | C | T | 1 | 1 | *AGAP1* | non-synonymous SNV |
| 2 | 239975235 | G | A | 1 | 1 | *HDAC4* | stopgain SNV |
| 3 | 9146456 | GG | AA | 1 | 1 | *SRGAP3* | immediate stopgain |
| 3 | 138426084 | G | A | 1 | 1 | *PIK3CB* | non-synonymous SNV |
| 4 | 62849200 | G | A | 1 | 1 | *LPHN3* | non-synonymous SNV |
| 4 | 87968307 | C | T | 1 | 1 | *AFF1* | non-synonymous SNV |
| 5 | 38952491 | G | A | 1 | 1 | *RICTOR* | synonymous SNV |
| 5 | 151784282 | G | A | 1 | 1 | *NMUR2* | synonymous SNV |
| 6 | 43156407 | G | C | 1 | 1 | *CUL9* | non-synonymous SNV |
| 6 | 73904187 | G | A | 1 | 1 | *KCNQ5* | non-synonymous SNV |
| 6 | 79735296 | G | A | 1 | 1 | *PHIP* | non-synonymous SNV |
| 7 | 83590778 | C | T | 1 | 1 | *SEMA3A* | non-synonymous SNV |
| 7 | 91726195 | C | T | 1 | 1 | *AKAP9* | stopgain SNV |
| 7 | 140453136 | A | T | 1 | 1 | *BRAF* | non-synonymous SNV |
| 8 | 12956881 | C | A | 1 | 1 | *DLC1* | non-synonymous SNV |
| 9 | 86518712 | G | A | 1 | 1 | *KIF27* | non-synonymous SNV |
| 10 | 69682726 | G | A | 1 | 1 | *HERC4* | stopgain SNV |
| 10 | 84745329 | G | A | 1 | 1 | *NRG3* | non-synonymous SNV |
| 10 | 104175794 | G | A | 1 | 1 | *PSD* | non-synonymous SNV |
| 11 | 9597603 | G | A | 1 | 1 | *WEE1* | non-synonymous SNV |
| 11 | 22381033 | G | A | 1 | 1 | *SLC17A6* | non-synonymous SNV |
| 12 | 12022648 | C | T | 1 | 1 | *ETV6* | non-synonymous SNV |
| 12 | 12022666 | C | T | 1 | 1 | *ETV6* | non-synonymous SNV |
| 12 | 18544059 | G | A | 1 | 1 | *PIK3C2G* | non-synonymous SNV |
| 12 | 71029779 | G | A | 1 | 1 | *PTPRB* | synonymous SNV |
| 14 | 56122791 | T | C | 1 | 1 | *KTN1* | synonymous SNV |
| 14 | 68244957 | T | G | 1 | 1 | *ZFYVE26* | non-synonymous SNV |
| 15 | 40595525 | C | T | 1 | 1 | *PLCB2* | synonymous SNV |
| 15 | 84657501 | G | A | 1 | 1 | *ADAMTSL3* | non-synonymous SNV |
| 16 | 9862780 | G | A | 1 | 1 | *GRIN2A* | synonymous SNV |
| 16 | 18881951 | G | A | 1 | 1 | *SMG1* | non-synonymous SNV |
| 16 | 23364356 | G | A | 1 | 1 | *SCNN1B* | synonymous SNV |
| 16 | 67644826 | G | A | 1 | 1 | *CTCF* | non-synonymous SNV |
| 17 | 45367088 | G | A | 1 | 1 | *ITGB3* | synonymous SNV |
| 17 | 49118942 | G | A | 1 | 1 | *SPAG9* | stopgain SNV |
| 17 | 58292135 | C | T | 1 | 1 | *USP32* | non-synonymous SNV |
| 19 | 6755165 | C | T | 1 | 1 | *SH2D3A* | non-synonymous SNV |
| 19 | 8994203 | G | A | 1 | 1 | *MUC16* | non-synonymous SNV |
| 19 | 9049663 | G | A | 1 | 1 | *MUC16* | synonymous SNV |
| 19 | 9060167 | G | A | 1 | 1 | *MUC16* | synonymous SNV |
| 19 | 9060524 | G | A | 1 | 1 | *MUC16* | synonymous SNV |
| 19 | 9060718 | G | A | 1 | 1 | *MUC16* | non-synonymous SNV |
| 19 | 9062951 | G | A | 1 | 1 | *MUC16* | synonymous SNV |
| 19 | 9065610 | G | A | 1 | 1 | *MUC16* | non-synonymous SNV |
| 19 | 55449566 | G | A | 1 | 1 | *NLRP7* | non-synonymous SNV |
| 20 | 57429312 | C | T | 1 | 1 | *GNAS* | non-synonymous SNV |
| 21 | 39795419 | C | T | 1 | 1 | *ERG* | non-synonymous SNV |
| 21 | 42840464 | C | T | 1 | 1 | *TMPRSS2* | synonymous SNV |
| X | 49083399 | G | A | 1 | 1 | *CACNA1F* | non-synonymous SNV |
| X | 135428763 | G | A | 1 | 1 | *GPR112* | synonymous SNV |
