# Supplementary material for: Molecular and genetic diversity in the metastatic process of melanoma
Source: J Pathol. 2014 Jan 27;233(1):39–50. doi: 10.1002/path.4318 (PMC4359751; doi:10.1002/path.4318)
Supplement: Supplementary file 14 — Coding sequence (exonic and splice site) mutations in patients 1–28 [file path0233-0039-sd14.zip › TableS7.doc]

**Table S7.** Exonic and splice site mutations in patient 4

| **Chrom** | **Position** | **Reference** | **Alternative** | **M1** | **M2** | **M3** | **Gene** | **Type** |
| --- | --- | --- | --- | --- | --- | --- | --- | --- |
| 1 | 45110422 | C | T | 1 | 1 | 1 | *RNF220* | synonymous SNV |
| 1 | 155170719 | T | G | 1 | 1 | 1 | *THBS3* | non-synonymous SNV |
| 2 | 170059408 | C | T | 1 | 1 | 1 | *LRP2* | synonymous SNV |
| 3 | 47161717 | G | A | 1 | 1 | 1 | *SETD2* | non-synonymous SNV |
| 5 | 13717526 | G | A | 1 | 1 | 1 | *DNAH5* | synonymous SNV |
| 5 | 14291249 | G | A | 1 | 1 | 1 | *TRIO* | non-synonymous SNV |
| 5 | 149439423 | G | A | 1 | 1 | 1 | *CSF1R* | non-synonymous SNV |
| 8 | 27516370 | G | A | 1 | 1 | 1 | *SCARA3* | non-synonymous SNV |
| 11 | 48145314 | C | T | 1 | 1 | 1 | *PTPRJ* | stopgain SNV |
| 13 | 28599030 | G | A | 1 | 1 | 1 | *FLT3* | non-synonymous SNV |
| 14 | 52520650 | G | A | 1 | 1 | 1 | *NID2* | non-synonymous SNV |
| 16 | 46638149 | G | A | 1 | 1 | 1 | *SHCBP1* | non-synonymous SNV |
| 19 | 57642499 | C | T | 1 | 1 | 1 | *USP29* | non-synonymous SNV |
| X | 123518420 | C | T | 1 | 1 | 1 | *TENM1* | non-synonymous SNV |
| 8 | 113933980 | G | A | 1 | 0 | 0 | *CSMD3* | synonymous SNV |
| 14 | 75514722 | C | A | 0 | 1 | 1 | *MLH3* | non-synonymous SNV |
| 15 | 63986720 | TG | - | 0 | 0 | 1 | *HERC1* | frameshift deletion |
| 19 | 10461737 | AC | - | 0 | 0 | 1 | *TYK2* | frameshift deletion |
| 20 | 40735551 | C | A | 0 | 0 | 1 | *PTPRT* | non-synonymous SNV |
