# Supplementary material for: Molecular and genetic diversity in the metastatic process of melanoma
Source: J Pathol. 2014 Jan 27;233(1):39–50. doi: 10.1002/path.4318 (PMC4359751; doi:10.1002/path.4318)
Supplement: Supplementary file 14 — Coding sequence (exonic and splice site) mutations in patients 1–28 [file path0233-0039-sd14.zip › TableS8.doc]

**Table S8.** Exonic and splice site mutations in patient 5

| **Chrom** | **Position** | **Reference** | **Alternative** | **M1** | **M2** | **Gene** | **Type** |
| --- | --- | --- | --- | --- | --- | --- | --- |
| **1** | 82456533 | C | T | 1 | 1 | *LPHN2* | non-synonymous SNV |
| **1** | 110167970 | CC | TT | 1 | 1 | *AMPD2* | non-frameshift substitution |
| **1** | 115256529 | T | C | 1 | 1 | *NRAS* | non-synonymous SNV |
| **1** | 228526029 | C | T | 1 | 1 | *OBSCN* | synonymous SNV |
| **2** | 26486326 | C | T | 1 | 1 | *HADHB* | non-synonymous SNV |
| **2** | 29551325 | C | T | 1 | 1 | *ALK* | non-synonymous SNV |
| **2** | 32678984 | G | A | 1 | 1 | *BIRC6* | non-synonymous SNV |
| **2** | 71762413 | G | A | 1 | 1 | *DYSF* | non-synonymous SNV |
| **2** | 179438371 | C | A | 1 | 1 | *TTN* | non-synonymous SNV |
| **2** | 179455345 | G | A | 1 | 1 | *TTN* | synonymous SNV |
| **2** | 179633479 | C | T | 1 | 1 | *TTN* | synonymous SNV |
| **2** | 179638267 | G | C | 1 | 1 | *TTN* | non-synonymous SNV |
| **2** | 209355369 | C | T | 1 | 1 | *PTH2R* | synonymous SNV |
| **2** | 238667426 | C | T | 1 | 1 | *LRRFIP1* | non-synonymous SNV |
| **3** | 2613222 | C | T | 1 | 1 | *CNTN4* | non-synonymous SNV |
| **3** | 58109341 | C | T | 1 | 1 | *FLNB* | synonymous SNV |
| **3** | 108833269 | G | A | 1 | 1 | *MORC1* | synonymous SNV |
| **3** | 148601565 | G | A | 1 | 1 | *CPA3* | non-synonymous SNV |
| **3** | 172098834 | T | G | 1 | 1 | *FNDC3B* | non-synonymous SNV |
| **4** | 62599143 | CC | TT | 1 | 1 | *LPHN3* | non-frameshift substitution |
| **4** | 175898305 | G | A | 1 | 1 | *ADAM29* | synonymous SNV |
| **5** | 1441601 | G | A | 1 | 1 | *SLC6A3* | synonymous SNV |
| **5** | 13830199 | G | A | 1 | 1 | *DNAH5* | non-synonymous SNV |
| **5** | 13883054 | G | A | 1 | 1 | *DNAH5* | non-synonymous SNV |
| **5** | 24488197 | C | T | 1 | 1 | *CDH10* | non-synonymous SNV |
| **5** | 24491937 | C | T | 1 | 1 | *CDH10* | splicing |
| **5** | 38950648 | G | A | 1 | 1 | *RICTOR* | non-synonymous SNV |
| **5** | 114860058 | A | G | 1 | 1 | *FEM1C* | non-synonymous SNV |
| **5** | 132403126 | G | A | 1 | 1 | *HSPA4* | synonymous SNV |
| **6** | 39398926 | C | T | 1 | 1 | *KIF6* | non-synonymous SNV |
| **6** | 43307196 | G | A | 1 | 1 | *ZNF318* | non-synonymous SNV |
| **6** | 51908440 | G | A | 1 | 1 | *PKHD1* | non-synonymous SNV |
| **6** | 70894800 | G | A | 1 | 1 | *COL19A1* | non-synonymous SNV |
| **6** | 83838884 | G | T | 1 | 1 | *DOPEY1* | non-synonymous SNV |
| **7** | 43506048 | C | T | 1 | 1 | *HECW1* | synonymous SNV |
| **7** | 91621522 | A | G | 1 | 1 | *AKAP9* | synonymous SNV |
| **7** | 148511122 | C | T | 1 | 1 | *EZH2* | non-synonymous SNV |
| **8** | 32463188 | C | T | 1 | 1 | *NRG1* | synonymous SNV |
| **8** | 68993058 | G | A | 1 | 1 | *PREX2* | synonymous SNV |
| **8** | 103359208 | G | A | 1 | 1 | *UBR5* | stopgain SNV |
| **8** | 113697928 | C | T | 1 | 1 | *CSMD3* | non-synonymous SNV |
| **9** | 84226788 | GCTC | - | 1 | 1 | *TLE1* | frameshift deletion |
| **10** | 84498400 | G | A | 1 | 1 | *NRG3* | non-synonymous SNV |
| **10** | 84745020 | G | A | 1 | 1 | *NRG3* | non-synonymous SNV |
| **11** | 11986048 | G | A | 1 | 1 | *DKK3* | non-synonymous SNV |
| **11** | 62591993 | C | A | 1 | 1 | *STX5* | non-synonymous SNV |
| **11** | 69625426 | C | T | 1 | 1 | *FGF3* | non-synonymous SNV |
| **11** | 100999588 | C | T | 1 | 1 | *PGR* | non-synonymous SNV |
| **11** | 121448065 | G | A | 1 | 1 | *SORL1* | non-synonymous SNV |
| **12** | 12022410 | C | T | 1 | 1 | *ETV6* | synonymous SNV |
| **12** | 57573659 | C | T | 1 | 1 | *LRP1* | synonymous SNV |
| **12** | 57850248 | C | T | 1 | 1 | *INHBE* | stopgain SNV |
| **12** | 80899871 | C | T | 1 | 1 | *PTPRQ* | non-synonymous SNV |
| **12** | 123814980 | G | A | 1 | 1 | *SBNO1* | non-synonymous SNV |
| **14** | 21485857 | G | A | 1 | 1 | *NDRG2* | synonymous SNV |
| **14** | 23242820 | T | A | 1 | 1 | *SLC7A7* | stoploss SNV |
| **14** | 103410705 | GG | AA | 1 | 1 | *CDC42BPB* | non-frameshift substitution |
| **15** | 28228537 | T | C | 1 | 1 | *OCA2* | non-synonymous SNV |
| **16** | 2052221 | G | A | 1 | 1 | *ZNF598* | non-synonymous SNV |
| **16** | 2115547 | C | T | 1 | 1 | *TSC2* | non-synonymous SNV |
| **16** | 22295257 | C | T | 1 | 1 | *EEF2K* | synonymous SNV |
| **16** | 58079075 | C | T | 1 | 1 | *MMP15* | non-synonymous SNV |
| **16** | 61687532 | C | T | 1 | 1 | *CDH8* | non-synonymous SNV |
| **17** | 8108241 | C | T | 1 | 1 | *AURKB* | non-synonymous SNV |
| **17** | 11778359 | G | A | 1 | 1 | *DNAH9* | non-synonymous SNV |
| **17** | 57126645 | G | A | 1 | 1 | *TRIM37* | non-synonymous SNV |
| **17** | 78321400 | C | T | 1 | 1 | *RNF213* | non-synonymous SNV |
| **19** | 9049163 | G | A | 1 | 1 | *MUC16* | non-synonymous SNV |
| **19** | 9068506 | C | T | 1 | 1 | *MUC16* | non-synonymous SNV |
| **19** | 56499195 | G | A | 1 | 1 | *NLRP8* | stopgain SNV |
| **19** | 57642485 | G | A | 1 | 1 | *USP29* | synonymous SNV |
| **21** | 19715884 | C | T | 1 | 1 | *TMPRSS15* | non-synonymous SNV |
| **21** | 43796780 | G | A | 1 | 1 | *TMPRSS3* | non-synonymous SNV |
| **22** | 46652620 | C | A | 1 | 1 | *PKDREJ* | synonymous SNV |
| **X** | 49076943 | C | T | 1 | 1 | *CACNA1F* | non-synonymous SNV |
| **X** | 135480045 | A | T | 1 | 1 | *GPR112* | non-synonymous SNV |
| **X** | 151092502 | C | T | 1 | 1 | *MAGEA4* | synonymous SNV |
