# Supplementary material for: Molecular and genetic diversity in the metastatic process of melanoma
Source: J Pathol. 2014 Jan 27;233(1):39–50. doi: 10.1002/path.4318 (PMC4359751; doi:10.1002/path.4318)
Supplement: Supplementary file 14 — Coding sequence (exonic and splice site) mutations in patients 1–28 [file path0233-0039-sd14.zip › TableS9.doc]

**Table S9.** Exonic and splice site mutations in patient 6

| **Chrom** | **Position** | **Reference** | **Alternative** | **M1** | **M2** | **Gene** | **Type** |
| --- | --- | --- | --- | --- | --- | --- | --- |
| 1 | 11189842 | G | A | 1 | 1 | *MTOR* | synonymous SNV |
| 1 | 12010487 | - | T | 1 | 1 | *PLOD1* | frameshift insertion |
| 1 | 19439348 | G | A | 1 | 1 | *UBR4* | non-synonymous SNV |
| 1 | 22987606 | G | A | 1 | 1 | *C1QB* | synonymous SNV |
| 1 | 27057674 | G | A | 1 | 1 | *ARID1A* | non-synonymous SNV |
| 1 | 115399260 | G | T | 1 | 1 | *SYCP1* | non-synonymous SNV |
| 1 | 228473889 | T | C | 1 | 1 | *OBSCN* | non-synonymous SNV |
| 3 | 58080649 | T | A | 1 | 1 | *FLNB* | non-synonymous SNV |
| 5 | 33964027 | G | A | 1 | 1 | *SLC45A2* | synonymous SNV |
| 5 | 82948551 | G | A | 1 | 1 | *HAPLN1* | stopgain SNV |
| 6 | 55216070 | C | T | 1 | 1 | *GFRAL* | synonymous SNV |
| 7 | 140453136 | A | T | 1 | 1 | *BRAF* | non-synonymous SNV |
| 8 | 80677450 | A | T | 1 | 1 | *HEY1* | synonymous SNV |
| 9 | 94519808 | T | C | 1 | 1 | *ROR2* | non-synonymous SNV |
| 9 | 96030149 | C | T | 1 | 1 | *WNK2* | non-synonymous SNV |
| 10 | 17061930 | G | - | 1 | 1 | *CUBN* | frameshift deletion |
| 12 | 15704490 | G | A | 1 | 1 | *PTPRO* | non-synonymous SNV |
| 12 | 49440175 | G | A | 1 | 1 | *MLL2* | non-synonymous SNV |
| 13 | 101944595 | A | T | 1 | 1 | *NALCN* | non-synonymous SNV |
| 17 | 7755510 | G | A | 1 | 1 | *KDM6B* | non-synonymous SNV |
| 17 | 11597252 | C | T | 1 | 1 | *DNAH9* | non-synonymous SNV |
| 17 | 57759127 | A | G | 1 | 1 | *CLTC* | synonymous SNV |
| 19 | 10270406 | G | A | 1 | 1 | *DNMT1* | non-synonymous SNV |
| 22 | 19752582 | C | T | 1 | 1 | *TBX1* | synonymous SNV |
| X | 35821228 | C | T | 1 | 1 | *MAGEB16* | synonymous SNV |
| X | 105855318 | G | T | 1 | 1 | *CXorf57* | non-synonymous SNV |
| X | 153073876 | C | T | 1 | 1 | *PDZD4* | non-synonymous SNV |
